# Deployable 3D architectures from wafer-fabricated precursors

**DOI:** 10.1038/s41467-026-76052-y

**Published:** 2026-07-25

**Authors:** Yue Wang, Kelvin Shum, Yuyang Song, Tian Chen

**Affiliations:** 1 Department of Mechanical & Aerospace Engineering, University of Houston, Houston, TX USA; 2 Toyota Research Institute of North America, Ann Arbor, MI USA; 3 Department of Aeronautics, Imperial College London, London, UK

**Keywords:** Mechanical engineering, Soft materials

## Abstract

We demonstrate that standard wafer fabrication can produce free-standing, mechanically stable, doubly-curved 3D structures with prescribed Gaussian curvature — a capability not previously achieved through semiconductor manufacturing. The stability of the deployed structures is realized through the bistable nature of their constituent auxetic unit cells. To impose prescribed Gaussian curvatures through deployment, we conformally flatten the target 3D mesh onto the 2D plane and locally tune the microstructure of each unit cell such that its second stable equilibrium occurs at the required isotropic expansion. The resulting precursor features a spatially heterogeneous tessellation. This generative method is validated on a spherical cap deployed from a flat disk through indentation, with deployment accuracy and structural stability confirmed numerically and experimentally. The method is further applied to a range of complex 3D shapes with both positive and negative curvatures. As a functional demonstration, paraboloidal reflectors with tunable focal lengths are fabricated and deployed, with reflected patterns agreeing with geometric optics predictions. This work establishes a route from flexible electronics towards deployable electronics, broadening the spectrum of realizable 3D semiconductor devices.

## Abstract (web summary)

Researchers show that standard chip-making techniques can transform flat patterns into stable, precisely curved 3D structures, enabling a new generation of functional 3D devices.

## Introduction

Three-dimensional mesoscale structures with doubly-curved geometries promise to transform medical engineering, curved electronics, and wireless communication^1–3^. Current efforts to realize three-dimensional electronic and photonic systems follow two distinct fabrication paradigms: direct three-dimensional microfabrication and planar wafer fabrication followed by transformation or assembly into three-dimensional forms^4–6^. Recent advances in multiphoton lithography (MPL), including two-photon polymerization, have significantly expanded the capabilities of the former, enabling freeform 3D architectures, writing on non-planar substrates, and a range of hybrid and transfer-based integration strategies^7–10^.

With the shared long-term goal of digital electronics in 3D, MPL advances this objective by offering geometric freedom and localized material patterning through serial, voxel-by-voxel writing^11^. This serial nature, together with the reliance on material-specific photoresists and sequential processing steps, represents a fundamental distinction from wafer-scale fabrication and can pose challenges for large-area scalability, throughput, and heterogeneous integration within established semiconductor workflows^12,13^.

Here we ask: can standard planar wafer fabrication be used to manufacture precursors that deploy uniquely, stably, and accurately to prescribed doubly-curved 3D geometries without a supporting substrate? We address this question by combining conformal mapping, bistable auxetic microstructure design, and multi-step lithography into a unified fabrication and deployment pipeline. This approach leverages existing planar wafer fabrication, where parallel processing, yield, and heterogeneous material integration are already mature, and combines it with mechanically programmed, bistable deployment to achieve prescribed three-dimensional architectures. We treat the 2D-to-3D deployment as an integral step of the manufacturing process: the planar precursor fabricated on the wafer is an intermediate product, and the deployed 3D structure is the final manufactured device^14–16^.

To achieve this, we address three fundamental challenges related to 1) accommodating the necessary mechanical stretch, 2) targeting specific 3D shapes, and 3) ensuring structural stability. First, Gauss’s Remarkable Theorem dictates that in-plane stretch and/or contraction is required to alter the Gaussian curvature of a surface^17^. To achieve significant curvature change, the necessary stretch often exceeds the rupture limits of thin film substrates^18,19^. To overcome this, one may constrain the designs to isometric deformations or bending^5,14,20,21^. Alternatively, a microstructural approach such as Kirigami, serpentine, or origami patterns may be adopted to accommodate a larger effective stretch of the overall material^22–27^.

Second, current techniques generally do not allow target geometries to be prescribed a priori. By virtue of periodic patterning, most 2D precursor designs are inherently 3D-shape-agnostic, i.e., they may equally conform to many different substrate geometries. As a corollary, it is also unknown, a priori, whether the required stretch of a specific contour exceeds the allowable stretch of the precursor, thereby causing tear and/or wrinkling^5,14,28^. Instead, we construct a conformal map of the target 3D design and generate a spatially varying 2D pattern accordingly. This 2D pattern can only deploy to that specific 3D shape, up to isometry.

Third, the mechanical stability of flexible electronics in the absence of a substrate remains undefined^16,29–32^, e.g., when the substrate is removed, most flexible electronics will collapse back to 2D. Mechanical bistability may address this challenge^33–35^, yet this has been rarely adopted on a meso- or micro-scale. Here, by engineering a second stable state in the target 3D design, we can imbue mechanical stability to 3D mesoscale structures after shape deployment^34–36^.

While target shape morphing through conformal mapping has been utilized in the literature^37,38^, including by the authors^34,39^, the central result of this work is that free-standing, mechanically stable, doubly-curved 3D structures with prescribed Gaussian curvature can be produced from standard wafer fabrication. We show 1) that masked lithography can fabricate bistable auxetic precursor structures with spatially heterogeneous aperiodic patterns algorithmically designed to target specific 3D shapes, 2) that these wafer-fabricated precursors can be released from the substrate and mechanically deployed uniquely and stably to the target 3D geometry without a supporting substrate, and 3) that the resulting free-standing 3D structures are mechanically stable and geometrically accurate, as confirmed through numerical simulation and experiment. This integration of target shape deployment and generalizable semiconductor processing enables a new class of devices including miniature antennas, conformal sensor arrays, micro-optical components, and human-interfacing medical implants^30,31,40^.

## Results

### Design & fabrication pipeline

**Fig. 1:**
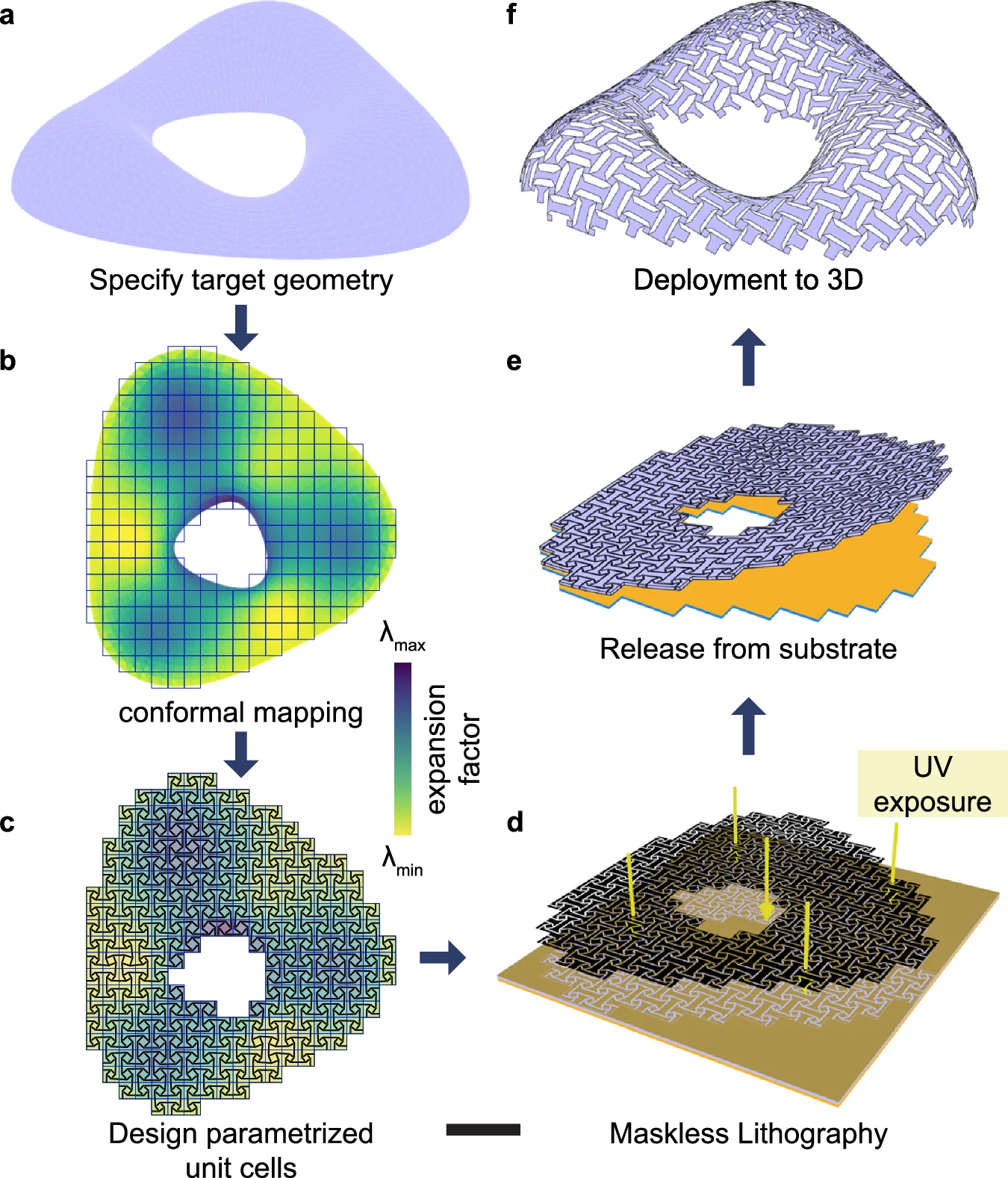
Pipeline for the manufacturing of deployable 3D mesoscale structures. All panels are schematic renderings of a single example design – an annulus with both positive and negative Gaussian curvature. **a** Target 3D surface mesh. **b** Conformally flattened 2D mesh, with the color spectrum indicating the local isotropic expansion factor *λ* per mesh face; a regular square grid is superimposed. **c** Spatially heterogeneous 2D precursor pattern obtained by tuning the geometric parameters of each unit cell so that its second stable equilibrium matches the local *λ*. **d** Maskless photolithography of the precursor pattern onto a polyimide-coated wafer; yellow arrows denote UV exposure from the maskless lithography system. The pattern is subsequently transferred into the polyimide film by reactive-ion etching. **e** Mechanical release of the patterned film from the wafer substrate. **f** Mechanical deployment of the free-standing precursor into the prescribed 3D geometry, stabilized by the bistability of the constituent unit cells. The shown scale bar is shared across all panels, and represents 500 μm.

We demonstrate a protocol that utilizes wafer fabrication to manufacture a 2D precursor, which is then deployed into a free-standing doubly curved 3D structure. Using an annulus geometry with both positive and negative Gaussian curvature as an example (Fig. 1a), the protocol first specifies a 3D surface mesh of the target geometry. A discrete conformal map is then computed^41^ to flatten this mesh onto the 2D plane, resulting in a 2D mesh with identical connectivity (Fig. 1b). The vertices are optimized to minimize angular distortion of the triangular mesh faces; this necessarily requires the area of each triangle to isotropically scale, as indicated by the expansion factor *λ*. A grid of squares of edge length 2*L* is superimposed onto the flattened mesh, and each square is assigned the local *λ* value from the mesh faces underneath. This areal scaling is mechanically enabled using a parametric unit cell that exhibits auxeticity and bistability with a tunable second equilibrium strain, *ε*_equil_. By setting *ε*_equil_ = *λ* − 1 per unit cell, the geometric parameters of each unit cell are determined, yielding a spatially heterogeneous 2D precursor pattern (Fig. 1c). The precursor is fabricated through a multi-step lithography process using polyimide PI-2611 (HD Microsystems Inc.) (Fig. 1d), released from the wafer substrate (Fig. 1e), and mechanically deployed into the prescribed 3D geometry, where it is stabilized by the bistable nature of its constituent unit cells (Fig. 1f).

### Mechanics of unit cell microstructure

**Fig. 2:**
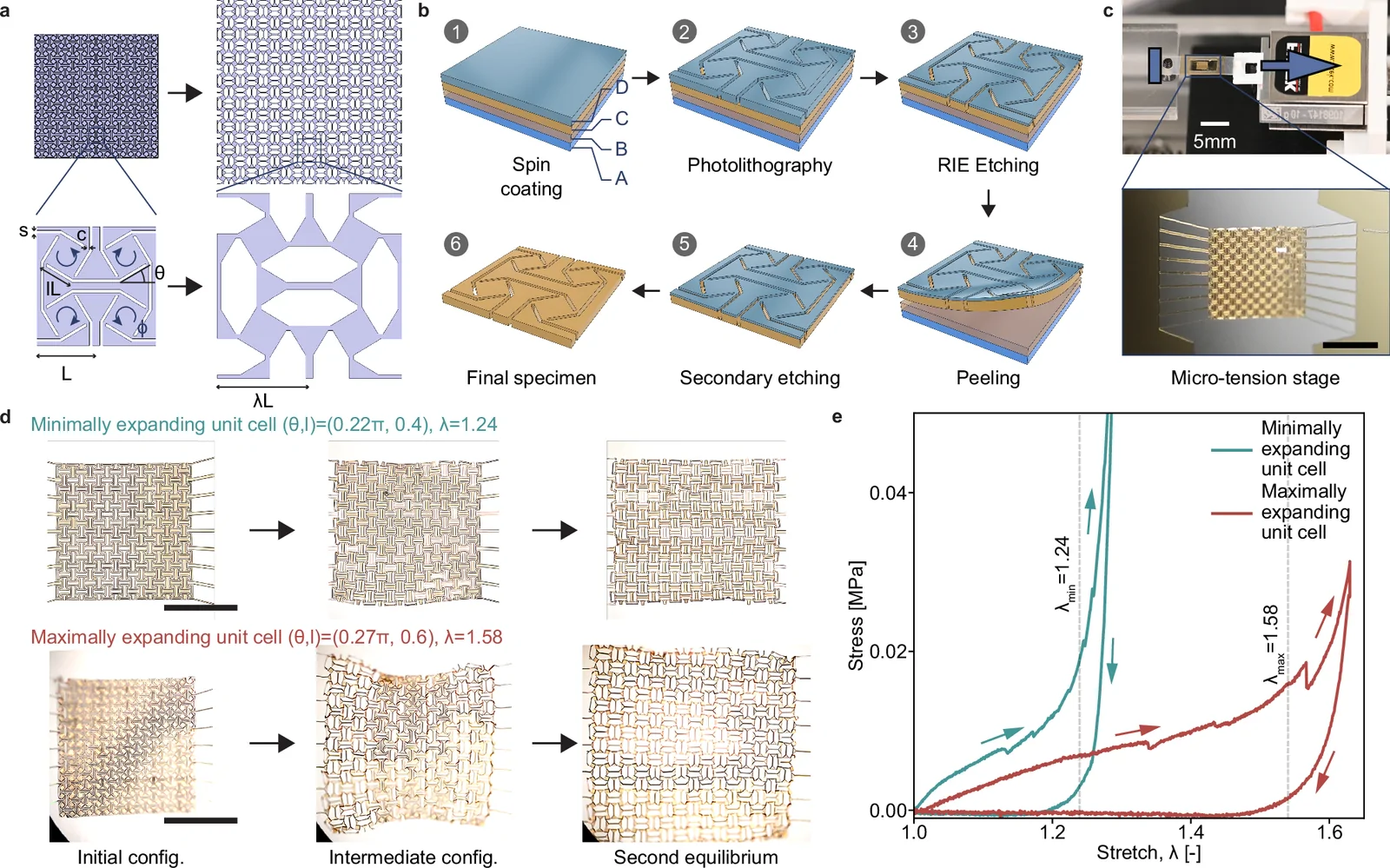
Mechanics of bistable auxetic unit cells. **a** Each 2D pattern is tessellated using a grid of unit cells. The microstructure of each unit cell features a number of slits. As the unit cells stretch, the inner squares counter-rotate. By changing internal geometric parameters *l* and *θ*, the stretch at the second equilibrium *λ* can be tuned. **b** Wafer fabrication protocol used by all specimens in this work featuring multiple masking and etching steps. The structure is physically peeled from the substrate prior to deployment. **c** Micro-tensile experimental setup featuring an 8 × 8 tessellation. **d** From all combinations of *l* and *θ*, two extremes of the *λ* spectrum are tension tested. The microscopic image sequence of the deformations of these are shown. **e** The stress-stretch curves of the two unit cells respectively. The vertical dashed lines indicate the stretch values at the second equilibria. **a**, **b** are not to scale. Unlabeled scale-bars represent 1 mm. Source data are provided as a Source Data file.

First, we analyze the kinematics and mechanics of the unit cell microstructure that enables auxeticity and bistability. All deployable surfaces in this work are tessellated with unit cells of the same initial edge length 2*L* = 220 μm (Fig. 2a). The microstructure of this rotating square auxetic pattern consists of a series of slits. These are defined by the inner square edge length of *l* and a tilting angle *θ*. The slits have a width of *s*, and they form four inner squares connected to the outer geometries with hinges of width *c*. The detailed geometric definition is found in Supplementary Note 2.1. This design is chosen because it simultaneously satisfies three properties required by the conformal deployment framework: (1) a Poisson’s ratio of *ν* = −1 between the rest and second equilibrium state, (2) mechanical bistability with a tunable second equilibrium stretch *λ* through the rotating square inclination *θ* and edge length *l*, and (3) isotropic expansion over the entire stretch range^26,35,42,43^. Isotropic expansion is critical because conformal maps prescribe a purely isotropic areal scaling *λ* at each point; a unit cell that expands anisotropically would introduce shear distortion incompatible with this framework.

When this unit cell undergoes uniaxial tension, the squares counter-rotate by an angle *ϕ* from 0 to 2*θ* to accommodate the stretch. Kinematically, this results in an isotropic expansion of the unit cell from the initial edge length 2*L* to 2*λL*^34^ where ${\lambda }_{{{{\rm{kinematic}}}}}=1+l\sin \theta$ assuming *c*, *s* = 0. Note that at all intermediate values of *ϕ*, kinematic incompatibility must be resolved through deformation. As a result, two stable equilibria exist at *ϕ* = 0 and 2*θ*.

We leverage wafer fabrication to manufacture all specimens in this manuscript. To facilitate experimental characterization of unit cells at the micron-scale, we fabricate 8 × 8 periodic tessellations of the different parametric variations. As shown in Fig. 2b, first, (i) the wafer is coated with (A) a silane coupling agent (3-Aminopropyl)triethoxysilane (APTES), (B,C) two layers of polyimide (PI-2611, 6 μm), and (D) a photoresist (AZ 12XT-20PL, 12 μm), (ii) then maskless photolithography is used to pattern the microstructure onto the photoresist, (iii) the sample is then uniformly etched until the depth of the slit on the top layer of PI is reduced by 75% using Reactive-ion etching (RIE). It is not etched through to minimize stress concentration during the peeling process that follows. In (iv), the upper layer of PI is mechanically peeled from the bottom, and (v) subjected to secondary etching until the slits are fully formed. (vi) Lastly, the sample is immersed in acetone to dissolve the remaining photoresist. Compared to widely used microfabrication approaches, such as employing sacrificial layers^32,44^, our protocol leverages the low surface adhesion between two polyimide layers to detach sample from substrate while minimizing stress concentrations. To evaluate the repeatability of the fabrication method, we measure the thickness of the polyimide film (6.08 ± 0.66 μm, *n* = 3) and of the photoresist (12.41 ± 0.16 μm, *n* = 3) in the thickness direction. In-plane, we compare the designed versus fabricated feature sizes of the critical slit and hinge dimensions. See Supplementary Note 4.1 for details.

A micro-mechanical stage is constructed to apply tension to the 8 × 8 periodic tessellations, which is designed to expand to a prescribed stretch in-plane (See Supplementary Note 5). The experiment is conducted under an optical microscope to measure the effective stretch. The boundary cells are connected with angled beams (Fig. 2c) which are designed to allow free expansion in the orthogonal direction, i.e., at the second equilibrium, the beams become parallel. From all combinations of *l* and *θ*, two extremes of the *λ* spectrum are experimentally tensioned to 1.2 times their respective second equilibria and relaxed. The resulting effective-stress and stretch curves show an initial stiffening followed by softening until the stretch equals to *λ* where the behavior rapidly stiffens again (Fig. 2d). The initial behavior is attributed to the partial opening of all unit cells as expected from linear elasticity. The softening is a result of the column by column triggering of bistability (Fig. 2e, see Supplementary Movie 1). Due to the imperfections in fabrication and the boundary conditions, the triggering force per column differ minutely. This causes some columns of unit cells to snap prior to others. At the expected *λ*, all unit cells are in their second equilibria, and further tensioning results in a significantly stiffer response. During unloading at the second equilibria, the reaction forces reach zero as indicative of static equilibrium. Further unloading results in out-of-plane buckling with negligible reaction forces.

Next, we quantify the mechanical response of all combinations of the two geometric parameters *θ* and *l* using Finite Element (FE) analyses. We identify a range of feasible *θ* from 0.1*π* to 0.28*π*, *l* from 0.2 to $0.8/(\sin \theta+\cos \theta )$, and set the increments at 0.01*π* and 0.02 respectively. Each unit cell microstructure is meshed using quadratic shell elements. Periodic boundary conditions are imposed on the four edges, and a stretch is prescribed from 1 to 1.2*λ*_kinematic_. The material behavior of polyimide is detailed in Supplementary Note 1. The detailed FE protocol is outlined in Methods and Supplementary Note 3.1. The resulting stress-stretch relationships inform the bistability of the different unit cells as well as the stretch at their second equilibria *λ* (if any). Of the parametric combinations of *θ* and *l* that result in a bistable microstructure as defined by the presence of a second local minimum in the strain energy, we identify the permissible spectrum of *λ* as between [1.2, 1.6].

With the parametric sweep, we construct a library of unit cells that feature the correspondence between the stretch at the second equilibrium *λ* and the input geometric parameters *θ* and *l*. Further, we identify the range of *λ* values that will result physically realizable microstructures that exhibit bistability. By examining the internal stresses, we validate that the hinges experience negligible plasticity. See Supplementary Note 3.2 for the results of all parametric combinations.

### Generative algorithm

Now, we discuss an algorithmic approach to design a spatially heterogeneous 2D tessellation that deploys to a prescribed 3D shape when all of its unit cells reach their respective second equilibrium. We observe that all unit cells in the design space expand isotropically up to their respective second equilibrium with a stretch equaling to *λ*. This leads us to use conformal mapping to calculate the necessary scalar field of *λ* as a function of the prescribed 3D shape. Once *λ* is calculated per coordinate on the 2D mesh, the values are then further translated to the unit cell geometric parameters *θ* and *l* using the unit cell library computed previously.

A conformal map is a function that locally preserves angles but not areas. Mechanically, this implies each material point may expand or contract but not distort in shape, which our unit cells satisfy. Thereby, if we abstract the prescribed 3D design by its Gaussian curvature *K*, we can simply relate *K* directly to the Laplacian of the scalar field *λ* (Eq. (1)),

1 $$K={\Delta }_{f}\log \lambda,$$

where Δ_*f*_ is the Laplace–Beltrami operator^28,45^.

**Fig. 3:**
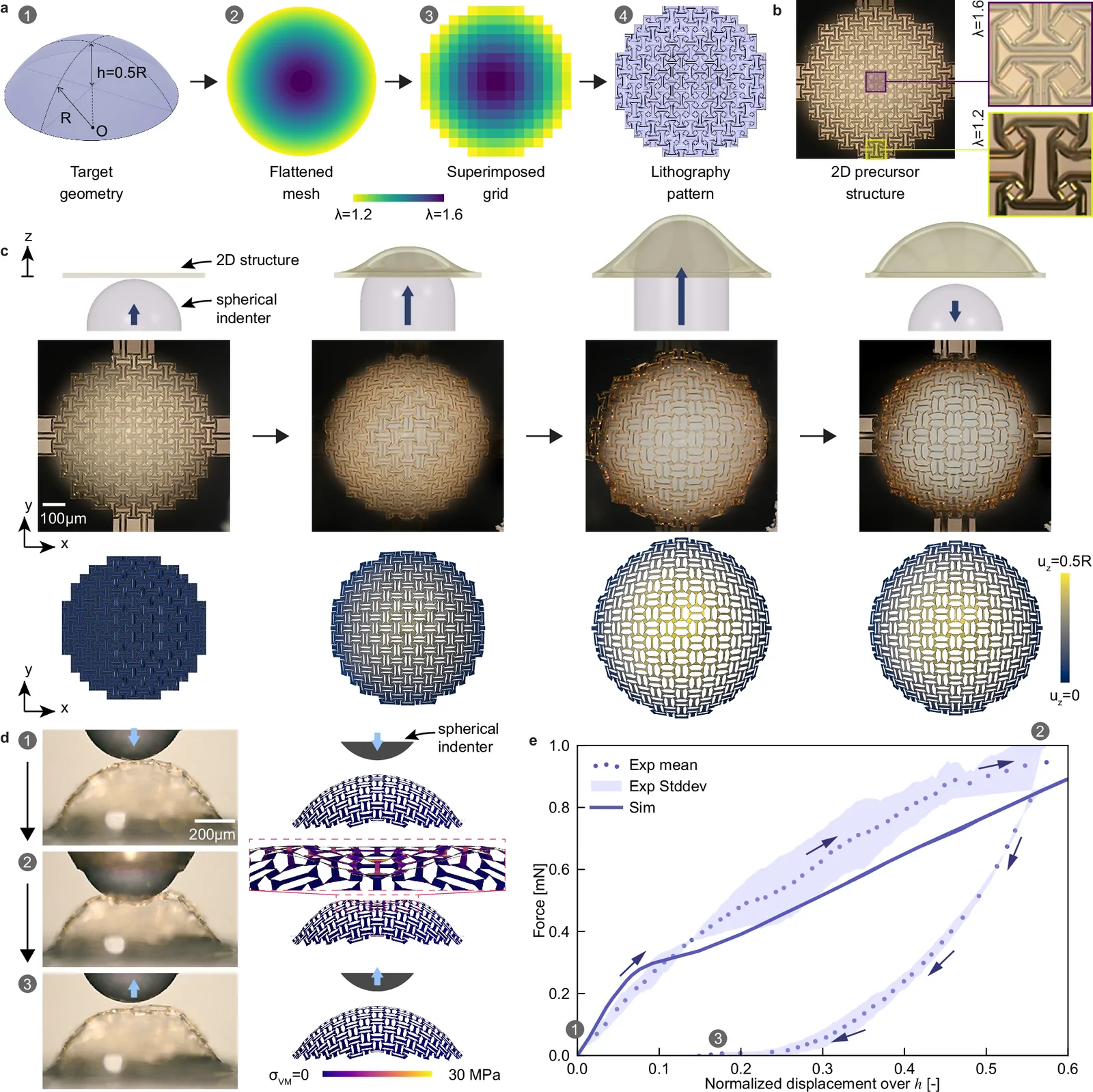
Design, fabrication and mechanical behavior of a deployable 3D spherical dome. **a** Schematic of the digital design pipeline converting a target 3D mesh into a lithography pattern: the dome mesh (1) is conformally flattened to the 2D plane (2) and grid-sampled to obtain the local scale factor *λ* (3), from which the geometric parameters *θ* and *l* of each unit cell are selected to produce the lithography pattern (4). **b** Microscope image of the wafer-fabricated 2D precursor; unit cells of differing *λ* are visible as a spatially varying microstructure. **c** 2D-to-3D deployment by a mechanical indenter, the final manufacturing step; the free-standing dome retains its shape after the indenter is withdrawn. Schematic, microscopy images, and FE snapshots are shown on the top, middle and bottom rows respectively. **d** Mechanical test of the deployed dome, with the indenter pressing downward against the dome resting on a flat surface. Rest, maximal indentation, and recovery are labeled 1, 2, 3; left and right columns are experimental photographs and FE snapshots, respectively. **e** Force-displacement response under indentation, comparing experiment (solid) and FE simulation (dashed); displacement is normalized by dome height. See Supplementary Movie 2. Source data are provided as a Source Data file.

Mechanistically speaking, the spatially varying degree of stretch on a surface will cause geometric frustration that “pop” the surface out into 3D^46^. This occurs when out-of-plane bending becomes more energetically favorable as compared to in-plane compression of the constituent material. Indeed, shape morphing that explicitly leverage this principle have been demonstrated^47–49^. To outline the algorithm, we target the deployment from a disk to a quarter-spherical dome, *h* = 0.5*R*, *R* = 1.35 mm (Fig. 3a,i). First, we algorithmically generate the 2D precursor, which features a spatially heterogeneous unit cell tessellation. While stereographic projection may be used to calculate a continuous *λ* field for such a canonical geometry^50^ (see Supplementary Note 2.2), we use Boundary First Flattening (BFF)^41^ for generality in processing arbitrary discrete geometries (see Supplementary Note 2.3). BFF computes conformal maps of input meshes by minimizing angular distortion while flattening from 3D to 2D. The local scale factor *λ* at each mesh face is computed as the areal change of each pair of corresponding mesh faces, i.e., $\lambda=\sqrt{{A}_{{{{\rm{3D}}}}}/{A}_{{{{\rm{2D}}}}}}$, where *A*_3D_ and *A*_2D_ are the areas of the corresponding mesh faces in 3D and 2D respectively (Fig. 3a,ii).

Next, a grid of squares of edge length 2*L* is overlaid on the conformally flattened 2D mesh. By inferring the *λ* of each square from the mesh faces underneath, we calculate the necessary *θ* and *l* of the unit cell microstructure to slot in the square (Fig. 3a,iii,iv). Should the *λ* resulting from BFF exceed the bounds of the permissible *λ* spectrum from the unit cell analysis, then no combinations of *θ* and *l* can satisfy the required scaling, and the target geometry cannot be deployed to with this particular microstructure without injecting singularities^51^.

The wafer fabrication protocol outlined in Fig. 2b is used to fabricate all 2D precursor patterns (Fig. 3b). For deployment, a spherical indenter (*r*_indenter_ = 0.4 mm) is used. The 2D pattern is fixed at the midpoint of the four edges. The indenter connected to a linear translation stage pushes up against the pattern until the vertical displacement reaches *d* = 1.2*h*. This is larger than the height of the dome to ensure each unit cell is stretched beyond their respective second equilibrium (Fig. 3c). The indenter is then retracted to show that the dome similarly retracts briefly until the intended height is reached, then detaches from the indenter.

To assess the repeatability of the deployment process, three dome structures are fabricated and deployed following the same procedure. The measured dome heights are 0.671 ± 0.018 mm and lateral spans are 2.29 ± 0.13 mm (mean ± s.d., *n* = 3 samples) respectively. This confirms consistent and repeatable deployment behavior across samples. While the dome height is achieved accurately, the systematic underestimation of the span (target: 2.70 mm, underestimation of 15%) is attributed to the boundary conditions of the physical structure, which constrain free expansion at the edge points. To quantify geometric accuracy, a 3D surface reconstruction of a representative deployed dome is generated using focal-plane stacking (see Supplementary Note 6). The pointwise geometric deviation between the reconstructed surface and the target spherical cap is mapped in Supplementary Fig. 12, confirming that deviations are concentrated at the rim (*δ* ≤0.14*R*), with the central region closely matching the target geometry. For further validation, to show that the 3D structure is not shaped as a result of indentation, we fabricate a periodic tessellation of unit cells following the same 2D outline. Following the identical deployment process, the structure becomes wrinkled and does not approximate a spherical cap (see Supplementary Note 6).

Using shell elements and static analysis, the FE simulation of deployment artificially expands the distance between every pair of neighboring unit cells from 2*L* to their respective 2*λL* where the *λ* of each pair is averaged. The total strain energy shows two local minima during the simulation, demonstrating the stability of the deployed 3D structure (see Supplementary Note 3.3). This method of simulated deployment can be universally applied as the target 3D design is not supplied a priori. By not applying displacements at the boundary nodes, this method circumvents the instabilities inherent in each unit cell. We find the resulting deployed 3D structure matches closely to the experimental counterpart with the simulated dome height being 0.71 mm. See Methods for details of the FE setup.

Lastly, to demonstrate structural stability, the indenter is flipped to press downward onto the deployed dome as it rests against a flat surface. The displacement of the indenter is increased until *d* = 0.6*h* upon which it reverses. Microscopic imaging of the deformation shows a behavior similar to those of continuum shells where a localized indent forms^52^. We note that the dome does not fully recover its original shape after significant indentation (Fig. 3d). This partial irreversibility is attributed to localized damage in the hinge regions of the unit-cell microstructure under off-path loading conditions. Specifically, indentation introduces localized bending and compressive stresses that are absent during deployment, leading to plastic yielding and buckling of hinge elements. Importantly, this behavior does not originate from the deployment process itself, which follows a prescribed deformation pathway to the second stable equilibrium.

The FE counterpart replicates the experimental setup and allows access to the internal stress state of the dome. The force-displacement behavior of the simulation matches well with that of the experiment for the loading portion (Fig. 3e). As the simulation model does not account for frictional dissipation (of the dome against the flat surface), the unloading is not predicted.

### Deployment of complex designs

**Fig. 4:**
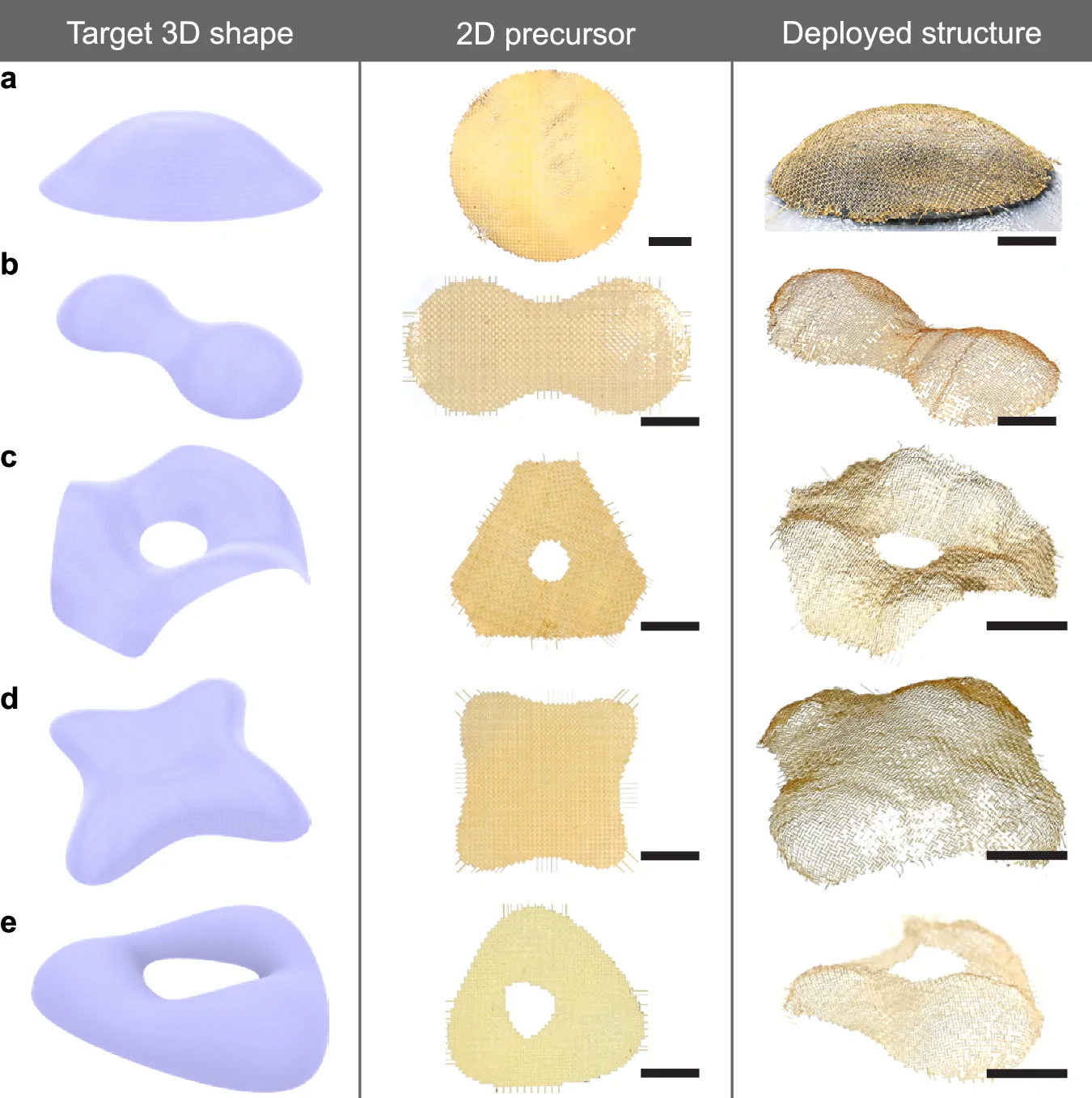
Experimental demonstration of complex target 3D designs. Five target shapes spanning different topologies and curvature distributions are supplied to the design algorithm, which generates the corresponding spatially heterogeneous 2D precursor patterns. These are wafer-fabricated and mechanically deployed into free-standing 3D structures. From top to bottom: **a** a cornea geometry (detailed in Supplementary Note 7); **b** a double dome separated by a region of negative Gaussian curvature; **c** a threefold-symmetric shape with elevated open boundaries; **d** four domes connected with center depression; and **e** the annulus of Fig. 1. Shapes (**c**) and (**e**) have Euler characteristic 0; the remainder have Euler characteristic 1. For each shape, the target 3D mesh, the 2D precursor and the deployed physical structure are shown on the left, center, and right columns, respectively. Scale bars, 2 mm.

To demonstrate the versatility of the 2D to 3D deployment methodology, we specify a number of target shapes with different topology and geometry (Fig. 4). These include: the cornea geometry shown in detail in Supplementary Note 7, a double dome with a negative curvature region in between^34^, a threefold rotational symmetric shape with open boundaries that are elevated^34^, four domes with a central depression^53^, and an annulus shown in Fig. 1^54^. The Euler characteristic of shapes shown in Fig. 4c, e are 0, whereas the others are 1^55^. In each case, the 2D precursors are fabricated in the 2D state with some boundary units connected to the substrate. The five shapes are designed using 1126, 667, 501, 590, 515 unit cells, respectively. Upon mechanical deployment through indentation, the 3D structures faithfully reproduce the target input geometries (see Supplementary Movie 3). As with the spherical dome (Fig. 3), deviations are localized at free and high-curvature boundaries, indicating that this systematic, boundary-driven deviation is geometric in origin rather than shape-specific. Note that not only is the global shape predictable, the spatial coordinates and connectivity of each unit cell can be inherently inferred. This allows an array of sensors to be precisely placed.

### Paraboloidal reflectors

We use paraboloidal reflectors as a representative geometry to demonstrate precise control over the curvature of the deployed 3D structures. Paraboloidal geometries provide a well-defined benchmark: their optical response is highly sensitive to surface curvature deviations, and the reflection pattern under collimated illumination can be analytically predicted. Furthermore, conventional fabrication of three-dimensional reflective surfaces often relies on high-precision machining, molding, or micro-scale 3D printing^56^. In contrast, our planar wafer-based fabrication strategy offers practical advantages for device integration, since wafer fabrication readily supports multilayer processing and the incorporation of additional functional materials such as sensing or electronic components.

**Fig. 5:**
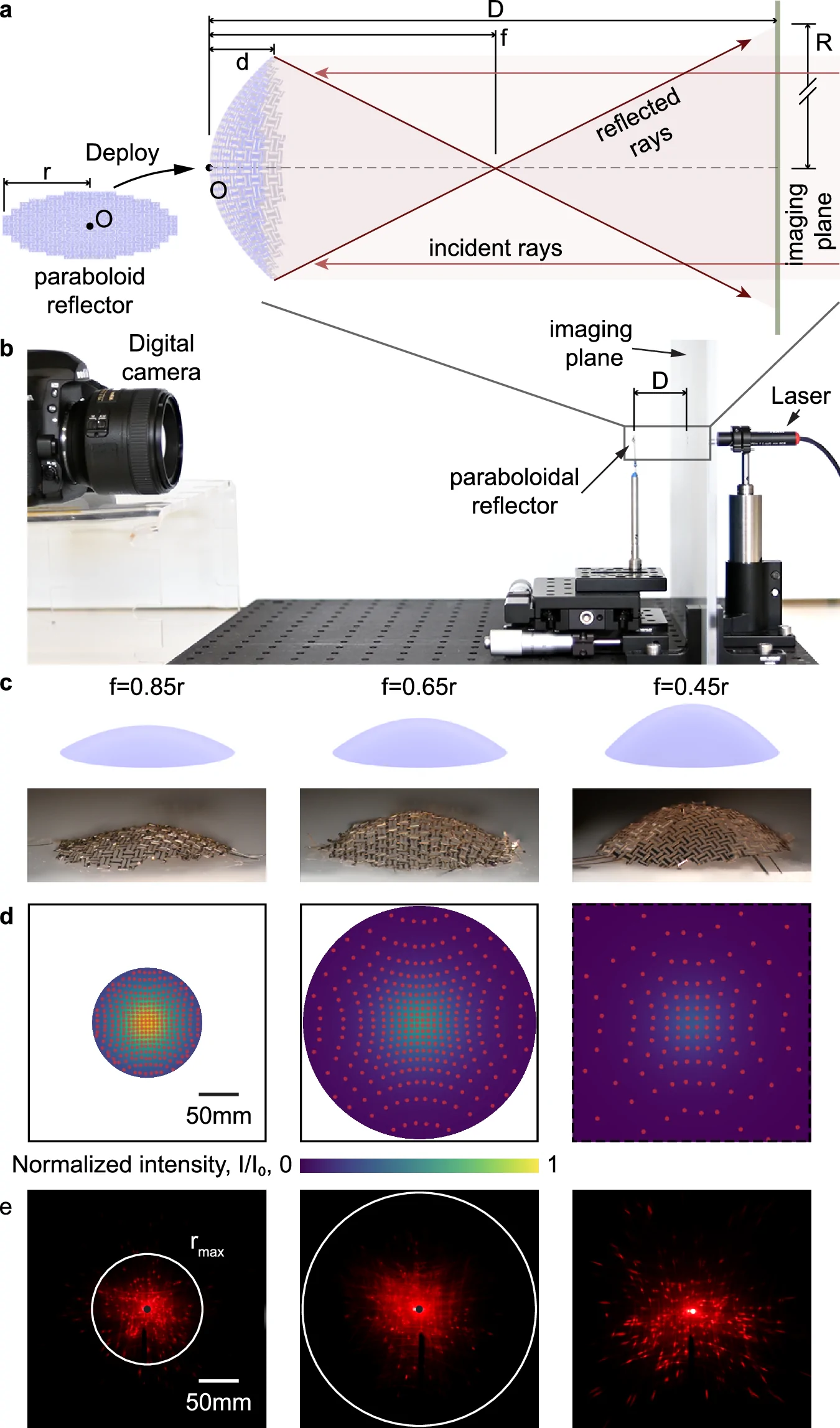
Doubly-curved paraboloidal reflectors with tunable focal distances. **a** The schematic diagram of experimental setup of the paraboloidal reflector shows the incident rays reflecting off the deployed reflector, converging at a focal point before diverging towards the imaging plane. **b** Photograph of the physical setup. **c** Target 3D design, and the fabricated, gold-coated, deployed reflectors (bottom) for focal lengths ranging from *f* = 0.85*r*, 0.65*r* to 0.45*r*. **d** Predicted reflected patterns at *D* = 40 mm from the continuous geometric-optics intensity field and discrete ray tracing in which one ray is traced per rotating-square mirror (overlaid dots, each marking a ray-plane intersection). The *f* = 0.45*r* pattern exceeds the plotted bounding box. **e** Measured reflected patterns with the analytically calculated maximum radii labeled. Source data are provided as a Source Data file.

To demonstrate this capability, we design three deployable reflectors, each targeting a different focal length, i.e., *f* = 0.85*r*, 0.65*r*, 0.45*r*, but all sharing the same diameter *r* = 3 mm. All three reflector designs employ the same number of bistable unit cells (*n* = 79) and the same edge length 2*L* = 220 μm. An optical characterization experiment is constructed to demonstrate accurate reflective behavior. The planar precursor is first deployed and then positioned at a distance *D* = 40 mm from an imaging plane. A collimated visible laser beam of 3 mm in diameter (Thorlabs, PL202, 635 nm, 1 mW) uniformly illuminates the entire surface of the reflector. Reflected rays propagate toward and are recorded on the opposing imaging plane (Fig. 5a). The pattern on the imaging plane is captured using a digital camera placed behind (Fig. 5b).

The 2D precursors are designed and fabricated using the wafer-scale lithography protocol described above. To enhance optical reflectivity, a thin gold layer is deposited onto the polyimide precursor as a final fabrication step. After mechanical deployment, the resulting 3D structures closely approximate the target paraboloidal geometries (Fig. 5c). To predict the reflected patterns, we employ a ray-based geometric optics model using the known parameters *f*, *r* and *D* (Fig. 5d). First, we consider the reflector as a smooth paraboloidal surface, and analytically derive the intensity profile as a function of the radial distance from the center. This continuous analysis confirms that the reflected intensity decays rapidly away from the center, explaining why the experimentally measured spatial extent of the reflected light appears smaller than the predicted geometric boundary. Recognizing that the deployed structure contains many voids, we additionally model the reflections from only the rotating squares within each bistable unit cell. Each square is assumed to be locally flat, and one ray per square is traced according to the local surface normal and projected onto the imaging plane. Each red dot in Fig. 5d therefore represents the intersection of a single reflected ray with the imaging plane. This discrete ray tracing captures the characteristic shape and spatial distribution of the reflected pattern, including the diagonal features arising from the square unit-cell architecture. As expected for paraboloidal geometries, reflected rays converge at the designed focal length before diverging toward the imaging plane (see Supplementary Note 8 for details).

In both continuous and discrete numerical approximations, the predicted ray distributions show a strong accumulation of reflected rays near the center for all three focal lengths, with the intensity decaying rapidly outward. From the discrete ray tracing, the predicted boundary radii of the illuminated regions on the imaging plane are *R* = 69.5 mm, 147 mm, and 381 mm respectively. Experimentally, reflected patterns are recorded under identical illumination conditions for all samples (Fig. 5e). The measured spatial extent of the reflected light is smaller than these predicted boundaries, consistent with the rapid intensity decay confirmed by the continuous model—little measurable light reaches the geometric boundary of the pattern. For the shortest focal length case (*f* = 0.45*r*), the illuminated region exceeds the size of the imaging plane.

Differences between the calculated and experimental patterns arise from effects not captured in the geometric optics model, including discretization by the square unit-cell architecture, light scattering, finite surface roughness and non-uniform gold coating, partial shadowing between neighboring unit cells, and deviations of the deployed geometry from the ideal paraboloidal shape. We further note that a direct quantitative comparison of reflected intensity distributions is complicated by the fact that the discrete model accounts only for reflections from the rotating squares, whereas the fabricated reflector includes additional reflective surfaces between unit cells. The comparison is therefore intended to validate curvature control and focal behavior rather than exact point-wise intensity agreement.

## Discussion

This work demonstrates that standard wafer fabrication can produce free-standing, mechanically stable, doubly-curved 3D structures with prescribed Gaussian curvature. By combining conformal mapping, bistable auxetic microstructure design, and multi-step lithography into a unified pipeline in which mechanical deployment serves as the final manufacturing step, we show that spatially heterogeneous 2D precursors can be algorithmically designed, lithographically fabricated, and mechanically deployed to target 3D geometries with accuracy confirmed both numerically and experimentally. The generality of the approach is demonstrated across a range of topologies and curvature distributions, and its geometric precision is validated through paraboloidal reflectors whose optical behavior agrees with analytical predictions. We note that the achievable curvature within a single continuous structure is bounded by the permissible range of *λ* of the particular microstructure design, which limits the sharpness of curvature gradients. This constraint can be addressed by stitching separately fabricated sections after deployment. Going beyond mechanical deployment, future research may investigate actuation mechanisms including magnetism^57^ or pneumatic inflation^54^ to enable autonomous deployment. By establishing a process-compatible pathway from planar wafer fabrication to prescribed 3D architectures, this work extends flexible electronics towards deployable electronics, broadening the spectrum of realizable 3D semiconductor devices^2^.

## Methods

### Material characterization

The mechanical response of the structural polyimide (PI-2611, HD Microsystems Inc.) is characterized by uniaxial tension following ASTM D882 for thin polymer films. Specimens are prepared using the same spin-coating and curing process as the device films (see Fabrication protocol): two 6 μm polyimide layers are spin-coated onto a silicon wafer, and the top layer is peeled off as a free-standing film, with the bottom layer serving as a release layer. The peeled film is cut into strips of 5 mm width and 80 mm length. Tensile tests are performed on a universal testing machine (Instron 68SC-2) under ambient conditions at a displacement rate of 5mm s^−1^ (*n* = 3 independent specimens). From the initial linear region of the stress-strain response, the Young’s modulus is 8.2 GPa; this value is used as the material input for all Finite Element simulations. The full stress-strain curve is given in Supplementary Note 1.

### Fabrication protocol

All fabrication is carried out in a cleanroom. Silicon wafers (100 mm diameter, P-type, (100) orientation, 1–100 *Ω* cm resistivity, thickness 525 ± 25 μm; WAFERPRO LLC) are used as substrates. The wafer is first treated with UV ozone for 15 min to grow a 500 nm silicon dioxide (SiO_2_) layer. Silanization is then performed by spin-coating a 0.01% volume-fraction solution of (3-Aminopropyl)triethoxysilane (APTES) at 4000 rpm to improve adhesion between the wafer and the polyimide.

A 6 μm polyimide layer (PI-2611, HD Microsystems Inc.) is deposited by spin-coating at 2000 rpm for 60 s, followed by a soft- and hard-bake protocol: 90° for 90 s, 150° for 90 s, then a ramp to 300° at ${5}^{\circ }\,{\min }^{-1}$, held at 300° for 30 min, and cooled to room temperature at the same rate. A second polyimide layer is deposited identically, giving a bilayer film. A 12 μm sacrificial layer of AZ 12XT-20PL positive photoresist is then spin-coated using a two-step profile (500 rpm for 30 s, then 2000 rpm for 60 s) and baked at 100° for 3 min.

The microstructure is patterned by maskless photolithography (Bruker SF-100 Lightning) using digital masks exported as DXF files. After exposure, the wafer is baked at 90° for 1 min and developed in AZ 300MIF for 3 min. The polyimide is selectively etched with an oxygen and SF_6_ plasma in a reactive-ion etching system (RIE-180) for 40 min, removing ~75% of the top polyimide layer thickness. Before the top layer is etched through, it is gently peeled from the bottom layer at the edge to avoid stress accumulation at the unit-cell hinges. The peeled film is placed on a spare wafer with the same side facing up, subjected to a further 15 min of RIE, then flipped and etched for an additional 3 min to fully open the slits without mechanically damaging the structures. Finally, the sample is immersed in acetone for 5 min to dissolve the remaining photoresist. The full protocol takes approximately two days and uses no strong acids or hazardous chemical baths. Dimensional accuracy of the fabricated features is evaluated in Supplementary Note 4.1.

### Experimental setup

Mechanical testing of the periodic tessellations and deployed structures uses a custom-built mesoscale system in which the testing and imaging modules are separated, allowing the assembly to be transported and placed under different optical systems. A 10g-capacity S-beam load cell (LSB200, Futek) is mounted on a motorized linear stage (Zaber) via a 3D-printed adapter, and a fixed boundary is provided by a position-adjustable right-angle bracket (AB90H, Thorlabs). Motion control and load-cell acquisition are integrated through a custom interface providing overload protection, displacement and rate control, and synchronized data logging. Deformation is imaged on an inverted optical microscope (Nikon Eclipse MA200) with a DS-Fi3 camera.

For tensile testing of the 8 × 8 periodic tessellations, slanted arms connect the leftmost and rightmost columns to the surrounding substrate to approximate periodic boundary conditions while allowing free transverse expansion; sacrificial bridges along the top and bottom edges prevent pre-stretching during handling and are cut immediately before testing. Specimens are loaded at a constant displacement rate of 5 μm s^−1^ under ambient conditions.

For dome deployment, the planar precursor is mounted flat on the observation stage and indented at its center by a 0.8 mm-diameter spherical head mounted on a linear translation stage oriented horizontally toward the microscope objective; images are captured at 0.1 mm increments of indenter travel. After deployment, the free-standing dome is transferred to the load cell and indented with a 3D-printed cylindrical shaft with a hemispherical tip through a single loading–unloading cycle at 0.01 mm s^−1^. Full descriptions of both setups are given in Supplementary Notes 5 and 6.

### Numerical simulations

All Finite Element (FE) simulations are performed in Abaqus/Standard (Static, General) with geometric nonlinearity enabled. All geometries are meshed with quadratic shell elements (S8R); a uniform seed is used over most of the geometry, with locally refined seeding at the hinge features. The polyimide is modeled as a linear elastic material with Young’s modulus *E* = 8200 MPa, Poisson’s ratio *ν* = 0.34, and density 1.54 × 10^−9^ t mm^−3^, assigned to a homogeneous shell section of thickness *t* = 6 μm. To reflect the as-fabricated geometry, the models use the measured feature dimensions rather than the nominal CAD values (Supplementary Note 4.1).

For unit-cell characterization, a quarter unit cell is generated parametrically and completed by three rotational mirror operations. Periodic boundary conditions are imposed by pairing nodes on opposite edges through equation constraints, with relative motion controlled by four auxiliary reference points outside the cell; a central node is pinned to remove rigid-body motion. Uniaxial loading is applied by prescribing displacement at the reference points along the *y* direction, up to 1.2 times the kinematically predicted second-equilibrium stretch.

For structural deployment, rather than displacing boundary nodes (which would trigger the per-cell instability), a local expansive displacement is prescribed between every pair of neighboring unit cells, extending their separation from 2*L* to 2*λ*_*i*_*L*, where *λ*_*i*_ is the average stretch of the corresponding pair (each interior cell has four neighbors). This displacement is imposed through axial connector elements. A small out-of-plane perturbation is applied as the first step to break *x*-*y* symmetry; for geometries with both convex and concave regions, the perturbation direction follows the sign of the mean curvature. The four edges connected to the surrounding membrane are constrained in *z*, and the center point is fixed in *x* and *y* by symmetry. The indentation simulation continues from the deployed state: a rigid hemispherical indenter (*E* = 200 GPa) is advanced downward against the dome to 60% of its height, using surface-to-surface, non-penetrating contact. Full details and the resulting energy and stress fields are given in Supplementary Note 3.

### Computational generation of lithography patterns

The lithography patterns are generated computationally in Python, and executed within Rhinoceros3D and its Grasshopper plug-in. A target 3D surface mesh is conformally flattened to the plane using Boundary First Flattening^41^ to obtain the per-face scale factor *λ*; a square grid is superimposed, each cell is assigned the unit-cell parameters *θ* and *l* from the precomputed finite-element library, and the resulting spatially heterogeneous pattern is assembled and exported as a DXF file for maskless lithography. NumPy^59^; SciPy^60^; pandas^61^; and Matplotlib^62^ are used within the Python algorithm. All custom scripts are deposited in the Zenodo repository^58^.

## Supplementary information

Supplementary Information

Peer Review file

Description of Additional Supplementary Files

Supplementary Movie 1

Supplementary Movie 2

Supplementary Movie 3

## Source data

Source Data

## Data Availability

The source data underlying all figures presented in this study are provided with this paper as a Source Data file. The complete dataset has been deposited in the Zenodo public repository and is available at 10.5281/zenodo.20393900^58^. No datasets with restricted access are associated with this study. Source data are provided with this paper.

## Code availability

All scripts and code for generating the lithography patterns and for Finite Element simulations are deposited in the same Zenodo repository (10.5281/zenodo.20393900)^58^, which includes a README documenting the dependencies and the steps required. Scripts are developed and tested with Python 3.10 and rely on NumPy^59^, pandas^61^, SciPy^60^, and Matplotlib^62^. The code is released under the MIT license, and no restrictions apply to its access or reuse.
