# Supplementary material for: Deployable 3D architectures from wafer-fabricated precursors
Source: Nat Commun. 2026 Jul 25;17:9047. doi: 10.1038/s41467-026-76052-y (PMC13507144; doi:10.1038/s41467-026-76052-y)
Supplement: Supplementary file 1 [file 41467_2026_76052_MOESM1_ESM.pdf]

Supplementary Information:
Deployable 3D architectures from wafer-fabricated precursors

Yue Wang[1], Kelvin Shum[1], Yuyang Song[2], and Tian Chen[1,3*]

[1]Department of Mechanical & Aerospace Engineering, University of Houston
[2]Toyota Research Institute of North America
[3]Department of Aeronautics, Imperial College London
[*]Corresponding author: tian.chen@imperial.ac.uk

Contents

1 Material Characterization of polyimide **2**

2 Geometric design **2**
2.1 Unit cell microstructure 2
2.2 Conformal map and stereographic projection 3
2.3 Discrete conformal mapping for arbitrary meshes 6
2.4 Lithography pattern generation algorithm 6

3 Finite Element Modeling **7**
3.1 FEM of the unit cell mechanics 7
3.2 Parametric sweep through unit cell microstructures 8
3.3 Simulation of deployment and indentation of the dome 8

4 Fabrication protocol **9**
4.1 Fabrication Evaluation 10

5 Tension of the periodic structures **11**

6 Spherical cap deployment and indentation **13**

7 Design of a cornea device **14**

8 Reflector design **15**
8.1 Intensity calculation 15

9 Scaling of bending stiffness **17**

Supplementary Note 1: Material Characterization of polyimide

Polyimide PI-2611 (HD Microsystems Inc.) is mechanically characterized with a tensile test. The material preparation process is identical to the one described in the Supplementary Note 4-Fabrication protocol below. First a 6 µm thick polyimide is spin-coated onto a silicon wafer twice. The wafer is then broken in half to gently peel off the top polyimide layer. To prepare specimens for tensile testing, the peeled polyimide film is cut into strips measuring 5 mm in width and 80 mm in length.

The mechanical properties of the polyimide are characterized according to the ASTM D882 standard for thin polymer films. Uniaxial tensile tests are conducted using a universal testing machine (Instron 68SC-2) under ambient conditions at a loading rate of $5\,\mathrm{mm\,s^{-1}}$.

The stress-strain response is recorded and shown in Fig. 1. From the initial linear portion of the curve, the Young's modulus is approximately 8.2 GPa. This value agrees well with the literature for spin-coated polyimide films [1], confirming the reliability of our fabrication and testing procedures. The measured modulus is used as the material input for subsequent Finite Element simulations.

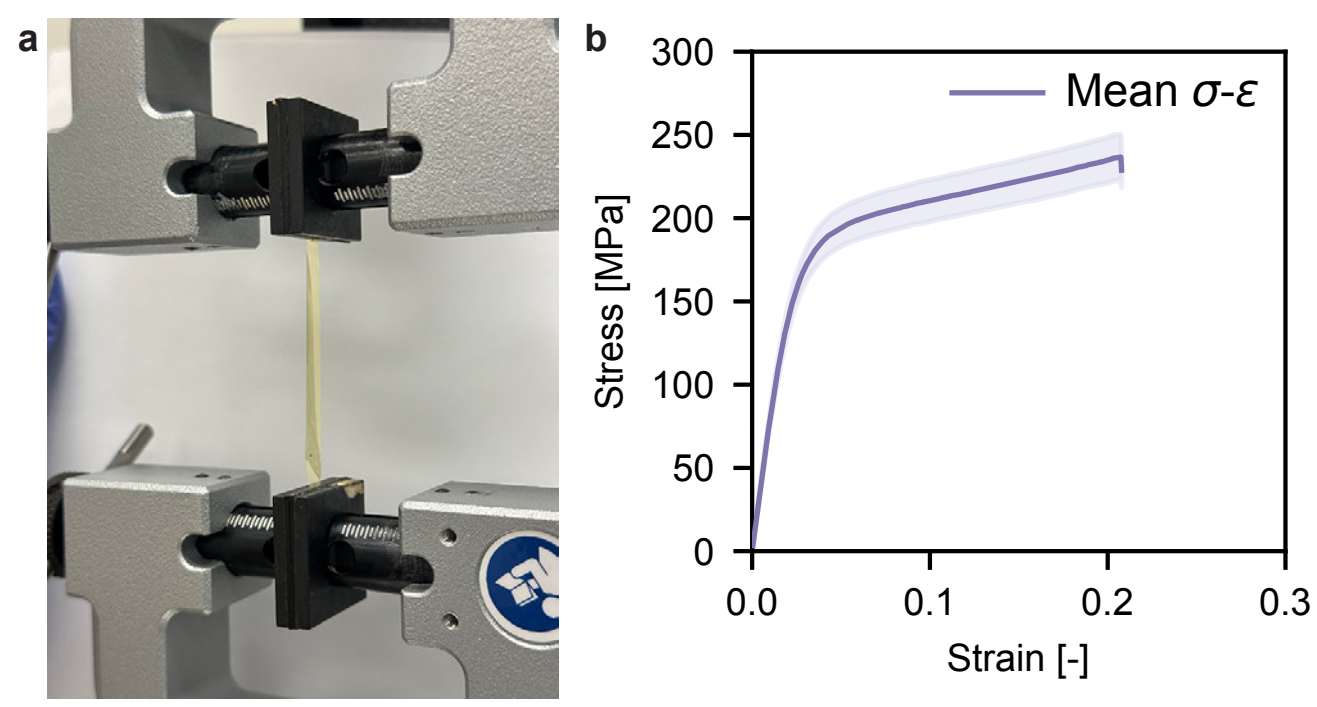

Supplementary Figure 1: **Material characterization of polyimide under uniaxial tension.** a) Photograph of the tensile testing setup with a polyimide strip specimen clamped between the grips. b) Stress-strain response of polyimide, where the solid line denotes the mean value and the shaded region indicates the variation across n=3 specimens. Source data are provided as a Source Data file.

Supplementary Note 2: Geometric design

2.1 Unit cell microstructure

The microstructure geometry of the unit cell is inspired by previous works in literature [2]. Instead of the laser-cutting, our model is constructed specifically for microscale fabrication via lithography, requiring precise definition of all slit outline parameters. As shown in Fig. 2a, we model a quarter unit cell and establish a rectangular coordinate system with the lower-left corner defined as the origin. Four kinked slits are embedded in the quarter unit cell in a pattern that exhibits 4-fold rotational symmetry. We describe a representative slit in the lower-left by first specifying the coordinates of three points along its centerline: points A, B, and D. The leading segment of the slit (AB) extends horizontally from the y-axis at a height of a, parallel to the x-axis, with a length of x. At point B, the trailing segment (BD) rotates by an angle θ from the leading segment AB. BD segment is oriented such that it points directly toward the kink point of the adjacent slit(B'). The distance between two neighboring kink points (BB') is denoted as l, which is defined as a

fraction of the quarter unit cell size L. For simplicity, we normalize the unit cell size by setting $L = 1$, and express all geometric quantities in terms of this dimensionless length. To determine the coordinates of points A and B, we introduce two geometric constraints of x and a:

$$x + a + l\cos\theta = 1 \tag{1}$$

$$a + l\sin\theta = x \tag{2}$$

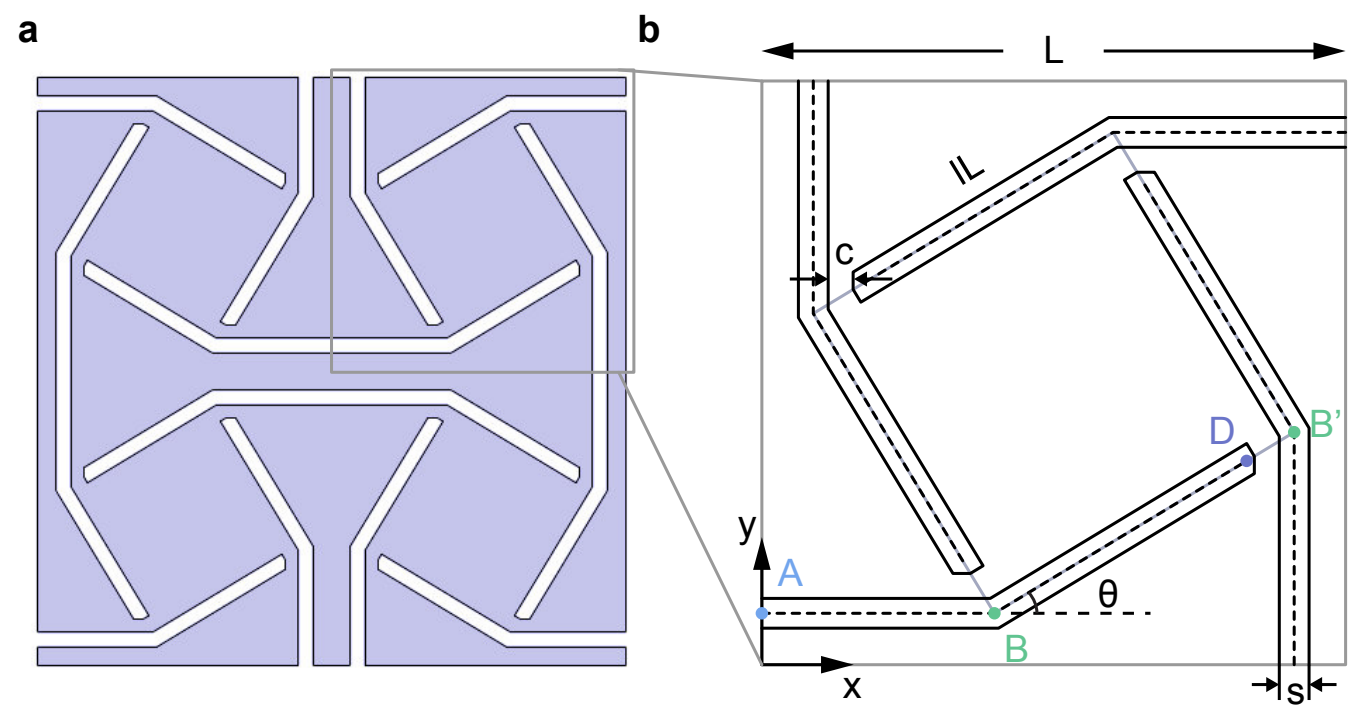

Supplementary Figure 2: **Parametric definition of unit cell microstructure.** a) Geometry of a representative unit cell with side length $2L$. b) Quarter unit cell illustrating the geometric parameters used in the design, including the half side length L, inner square edge length l, gap width s, hinge width c, and the tilting angle θ of the inner square.

Solving these equations yields:

$$a = \frac{1}{2} - \frac{l}{2}(\cos\theta + \sin\theta) \tag{3}$$

$$x = \frac{1}{2} - \frac{l}{2}(\cos\theta - \sin\theta) \tag{4}$$

Point D lies along the segment BB', and its coordinate is determined by the slit width s and the hinge width c, as labeled in Fig. 2b. Both c and s are defined empirically based on experimental optimization for the maskless lithography system (SF-100). These parameters remain constant throughout all subsequent simulations and analyses.

The coordinates of point A, B and D are as follow:

$$A = (0,\, a)$$

$$B = (x,\, a)$$

$$D = \left(x + l\cos\theta - c - \frac{s}{2} - \frac{s\sin\theta}{2},\; a + \left(l\cos\theta - c - \frac{s}{2} - \frac{s\sin\theta}{2}\right)\tan\theta\right)$$

The control points defining the outline of the slit are then obtained by shifting the coordinates of points A, B, and D by $s/2$ in the direction perpendicular to the slit centerline. To construct the four kinked slits within a quarter unit cell, the initial slit geometry is rotated by 90° successively. By subsequently mirroring the quarter unit cell across both the horizontal and vertical axes, a complete bistable unit cell is formed.

2.2 Conformal map and stereographic projection

Conformal map has the property of preserving angles and isotropic scaling: the small area around each point stretches or shrinks evenly in all directions. This allows us to flatten a 3D surface onto a plane without distorting the shapes near every material point.

Consider a map $f\colon \Omega \mapsto \mathbb{R}^3$, where $\Omega \subset \mathbb{R}^2$ and f maps this domain to a surface in $\mathbb{R}^3$. The directional derivative of f at $\boldsymbol{Q} \in \Omega$ relative to the direction $\mathbf{X}$ is given by $J\mathbf{X}$ where J is the Jacobian matrix of f at $\boldsymbol{Q}$. Intuitively, the Jacobian describes how the map f stretches the 2D vector $\mathbf{X}$. We say that f is conformal if, for any point $\boldsymbol{Q} \in \Omega$,

$$J\mathbf{X} \cdot J\mathbf{Y} = \lambda(\boldsymbol{Q})^2\, \mathbf{X} \cdot \mathbf{Y} \tag{5}$$

where $\lambda(\boldsymbol{Q}) > 0$ is the local scale factor of the mapping. Geometrically, this means that the map scales all vectors at $\boldsymbol{Q}$ by the same factor $\lambda(\boldsymbol{Q})$ regardless of their direction. This is the defining property of isotropic scaling. This relation also indicates that $\lambda(\boldsymbol{Q})$ can be determined from the mapped vectors $J\mathbf{X}$ and $J\mathbf{Y}$ in $\mathbb{R}^3$ and their pre-images $\mathbf{X}$ and $\mathbf{Y}$ in $\mathbb{R}^2$ [3].

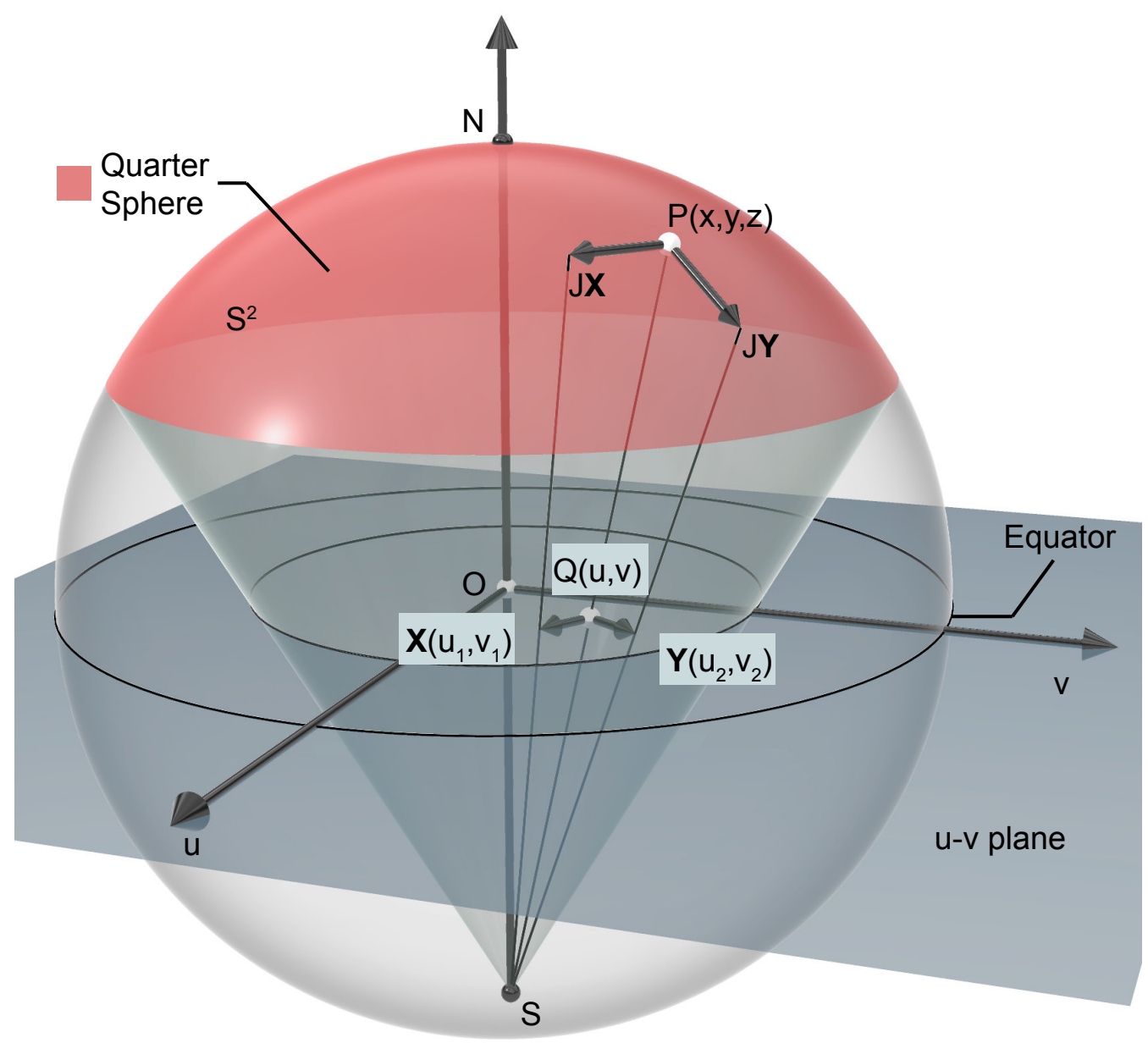

Supplementary Figure 3: **Stereographic projection of a unit sphere S^2 onto the uv-plane.** $\boldsymbol{P}$ is an arbitrary point on unit sphere. The vectors $J\mathbf{X}$ and $J\mathbf{Y}$ form a pair of tangent vectors at $\boldsymbol{P}$.

Stereographic projection provides a conformal map from the sphere to the plane, preserving local angles and mapping circles on the sphere to circles (or straight lines) in the planar domain [4]. Here, we use this mapping to explicitly derive the local scale factor λ for transforming a quarter sphere (i.e., a spherical cap with height equal to one quarter of the sphere diameter) into a planar precursor. As shown in Fig. 3, we consider the unit sphere S^2 in $\mathbb{R}^3$, as defined by the set of all points (x, y, z) satisfying $x^2 + y^2 + z^2 = 1$. The south pole is the point $\boldsymbol{S} = (0, 0, -1)$, and we let $S^2 \setminus \{\boldsymbol{S}\}$ represent the sphere excluding the south pole. In stereographic projection, each point $\boldsymbol{P} \in S^2 \setminus \{\boldsymbol{S}\}$ is mapped onto the uv-plane (i.e., the plane $z = 0$) by drawing a line from the south pole $\boldsymbol{S}$ through $\boldsymbol{P}$ and identifying its intersection with the plane at $\boldsymbol{Q}$. For the quarter sphere (colored in red in Fig. 3), points lying on the same circle of latitude (e.g., the rim of the dome) are projected onto concentric circles centered at the origin in the uv-plane, thereby defining the boundary of the planar domain corresponding to the quarter-sphere projection.

Using a simple Cartesian coordinate transformation, the projection between $\boldsymbol{Q}(u, v)$ and $\boldsymbol{P}(x, y, z)$ can be expressed as:

$$(u, v) = \left(\frac{x}{1-z}, \frac{y}{1-z} \right) \tag{6}$$

with the inverse,

$$(x, y, z) = \left(\frac{2u}{\xi}, \frac{2v}{\xi}, \frac{\xi - 2}{\xi} \right), \tag{7}$$

with $\xi = 1 + u^2 + v^2$.

The Jacobian matrix of the mapping is given by the partial derivatives of (x, y, z) with respect to (u, v), i.e.,

$$J = \begin{bmatrix} \frac{\partial x}{\partial u} & \frac{\partial x}{\partial v} \\ \frac{\partial y}{\partial u} & \frac{\partial y}{\partial v} \\ \frac{\partial z}{\partial u} & \frac{\partial z}{\partial v} \end{bmatrix} = \begin{bmatrix} \frac{2\xi - 4u^2}{\xi^2} & \frac{-4uv}{\xi^2} \\ \frac{-4uv}{\xi^2} & \frac{2\xi - 4v^2}{\xi^2} \\ \frac{4u}{\xi^2} & \frac{4v}{\xi^2} \end{bmatrix} \tag{8}$$

For a point $\boldsymbol{Q}(a, b)$ in the uv-plane, substituting $u = a$ and $v = b$ into Eq. (8) yields the Jacobian matrix at $\boldsymbol{Q}$:

$$J_{\boldsymbol{Q}} = \begin{bmatrix} \frac{2\gamma - 4a^2}{\gamma^2} & \frac{-4ab}{\gamma^2} \\ \frac{-4ab}{\gamma^2} & \frac{2\gamma - 4b^2}{\gamma^2} \\ \frac{4a}{\gamma^2} & \frac{4b}{\gamma^2} \end{bmatrix}, \quad \gamma = 1 + a^2 + b^2 \tag{9}$$

We consider any two in-plane vectors $\mathbf{X} = (u_1, v_1)$ and $\mathbf{Y} = (u_2, v_2)$ defined at $\boldsymbol{Q}$. The directional derivative of f at $\boldsymbol{Q} \in \Omega$ along the direction $\mathbf{X}(u_1, v_1)$ is given by:

$$J\mathbf{X} = \left[\frac{2\gamma - 4a^2}{\gamma^2} u_1 - \frac{4ab}{\gamma^2} v_1, \quad \frac{-4ab}{\gamma^2} u_1 + \frac{2\gamma - 4b^2}{\gamma^2} v_1, \quad \frac{4a}{\gamma^2} u_1 + \frac{4b}{\gamma^2} v_1 \right] \tag{10}$$

Similarly, the directional derivative of f at $\boldsymbol{Q}$ along the direction $\mathbf{Y}$ is given by:

$$J\mathbf{Y} = \left[\frac{2\gamma - 4a^2}{\gamma^2} u_2 - \frac{4ab}{\gamma^2} v_2, \quad \frac{-4ab}{\gamma^2} u_2 + \frac{2\gamma - 4b^2}{\gamma^2} v_2, \quad \frac{4a}{\gamma^2} u_2 + \frac{4b}{\gamma^2} v_2 \right] \tag{11}$$

Once simplified, the inner product of the two three-dimensional vectors $J\mathbf{X}$ and $J\mathbf{Y}$, after being mapped, is as follows:

$$J\mathbf{X} \cdot J\mathbf{Y} = \frac{4(u_1 u_2 + v_1 v_2)}{\gamma^2} \tag{12}$$

From Equation (5),

$$\lambda(\boldsymbol{Q}) = \sqrt{\frac{J\mathbf{X} \cdot J\mathbf{Y}}{\mathbf{X} \cdot \mathbf{Y}}} \tag{13}$$

Since $\mathbf{X} \cdot \mathbf{Y} = u_1 u_2 + v_1 v_2$, and substitute Equation (12) into Equation (13), we get

$$\lambda(\boldsymbol{Q}) = \frac{2}{\gamma} = \frac{2}{1 + a^2 + b^2} \tag{14}$$

which shows that the scale factor $\lambda(\boldsymbol{Q})$ depends only on the coordinate of the point $\boldsymbol{Q}$ and is independent of the chosen in-plane directions $\mathbf{X}$ and $\mathbf{Y}$. This reflects the conformal property of the mapping, whereby the neighborhood of a point is scaled isotropically in all directions. Consequently, this relation quantifies how stereographic projection locally scales planar vectors when mapping the domain onto the three-dimensional surface.

2.3 Discrete conformal mapping for arbitrary meshes

The method described in Supplementary Note 2.2 above computes a continuous field of the scale factor λ of canonical shapes such as a sphere. To analyze the local deformation of arbitrary 3D surfaces, we discretize the surface into a triangulated surface mesh and compute λ at each mesh element using the Boundary First Flattening (BFF) algorithm [5].

BFF is an optimization-based algorithm for computing conformal maps from 3D surfaces to the 2D plane. It defines a boundary first, once the boundary is known, the map can be easily extended over the rest of the domain. It preserves the angles of the original surface while flattening it, ensuring that local geometric features are faithfully represented. The algorithm solves for a 2D parameterization that minimizes angular distortion and aligns with conformal principles. Here, the boundary is flattened with minimal area distortion, allowing the shape to adjust naturally while maintaining local scaling accuracy.

2.4 Lithography pattern generation algorithm

The generative algorithm is implemented in Python and visualized using Rhinoceros3D and the Grasshopper plugin. First, the target 3D triangular surface mesh is created. BFF is used to calculate a discrete conformal map of the 3D mesh. This map is represented by a 2D mesh with the exact same connectivity as the 3D mesh.

A square grid is superimposed onto the 2D mesh, and a stretch value λ is inferred using weighted average from the mesh element that each square encompasses. The internal geometric parameters θ and l are then derived from the λ stretch. This is done with the help of Finite Element simulation of each parametric variation of the unit cell (described in Supplementary Note 3). From this computation, we collect the correspondence between θ, l and λ, where λ is the stretch at the second equilibrium state of the bistable unit cell. Since there may be multiple (θ, l) pairs that yield the same λ, we choose the one with the largest tangent stiffness at a stretch of λ. Once every unit cell microstructure is defined, the vertices along the edges of each square are merged with the corresponding vertices of the neighboring squares to ensure physical compatibility.

The pattern is generated in Rhinoceros3D as a set of geometric outlines and then exported as a DXF file. In this file, the regions intended to remain unexposed are assigned a black color fill, while the rest of the areas are left blank. This formatting allows the maskless lithography system to interpret the design correctly, exposing only the desired regions while leaving the filled areas unexposed.

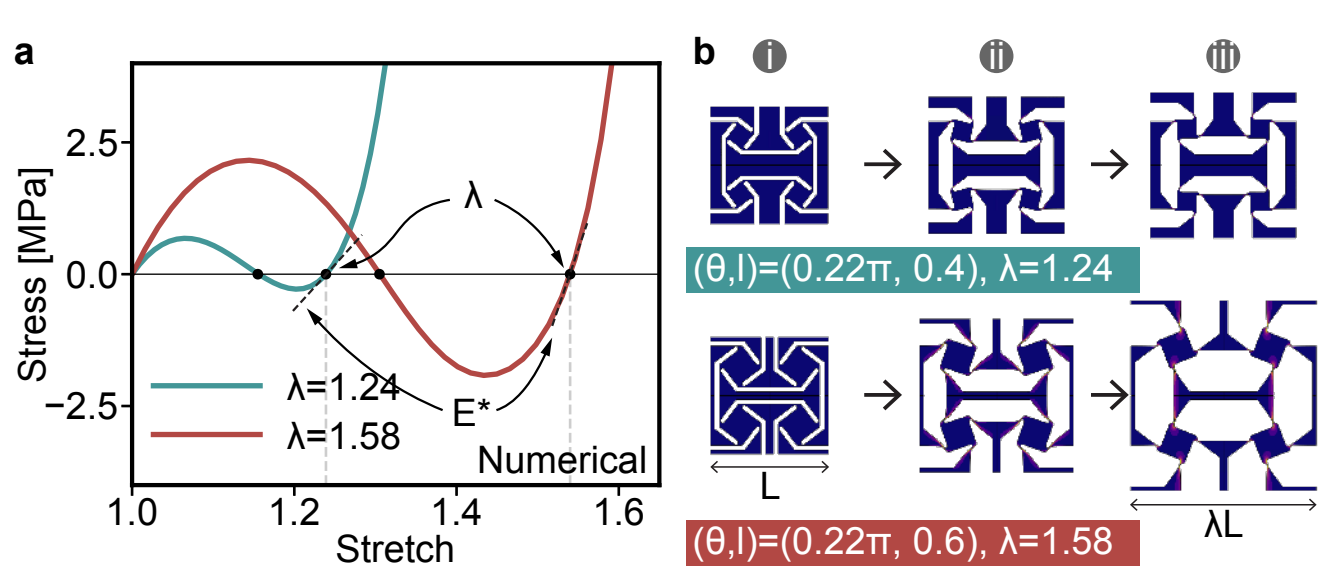

Supplementary Figure 4: **Finite Element (FE) simulation of unit cells.** a) Stress-strain plots of the two unit cells at two extreme ends of the λ spectrum. b) The corresponding isotropic deformation sequence of these two unit cells under periodic boundary stretching. Source data are provided as a Source Data file.

Supplementary Note 3: Finite Element Modeling

3.1 FEM of the unit cell mechanics

The unit cell geometry is generated parametrically in Abaqus using a Python script. The quarter unit cell is defined with the line segments connected with control points described in Supplementary Note 2.1. The geometry is meshed using S8R shell elements. A uniform mesh size is used in most regions, but the hinge-like features have locally refined seeding.

The polyimide material is modeled with the following parameters, Young's modulus E = 8200 MPa, Poisson's ratio $\nu = 0.34$, and density $1.54 \times 10^{-9}\,\mathrm{t\,mm^{-3}}$. A homogeneous shell section of uniform thickness $t = 6\,\mu\mathrm{m}$ is assigned. The mesh is mirrored rotationally three times to complete the assembly.

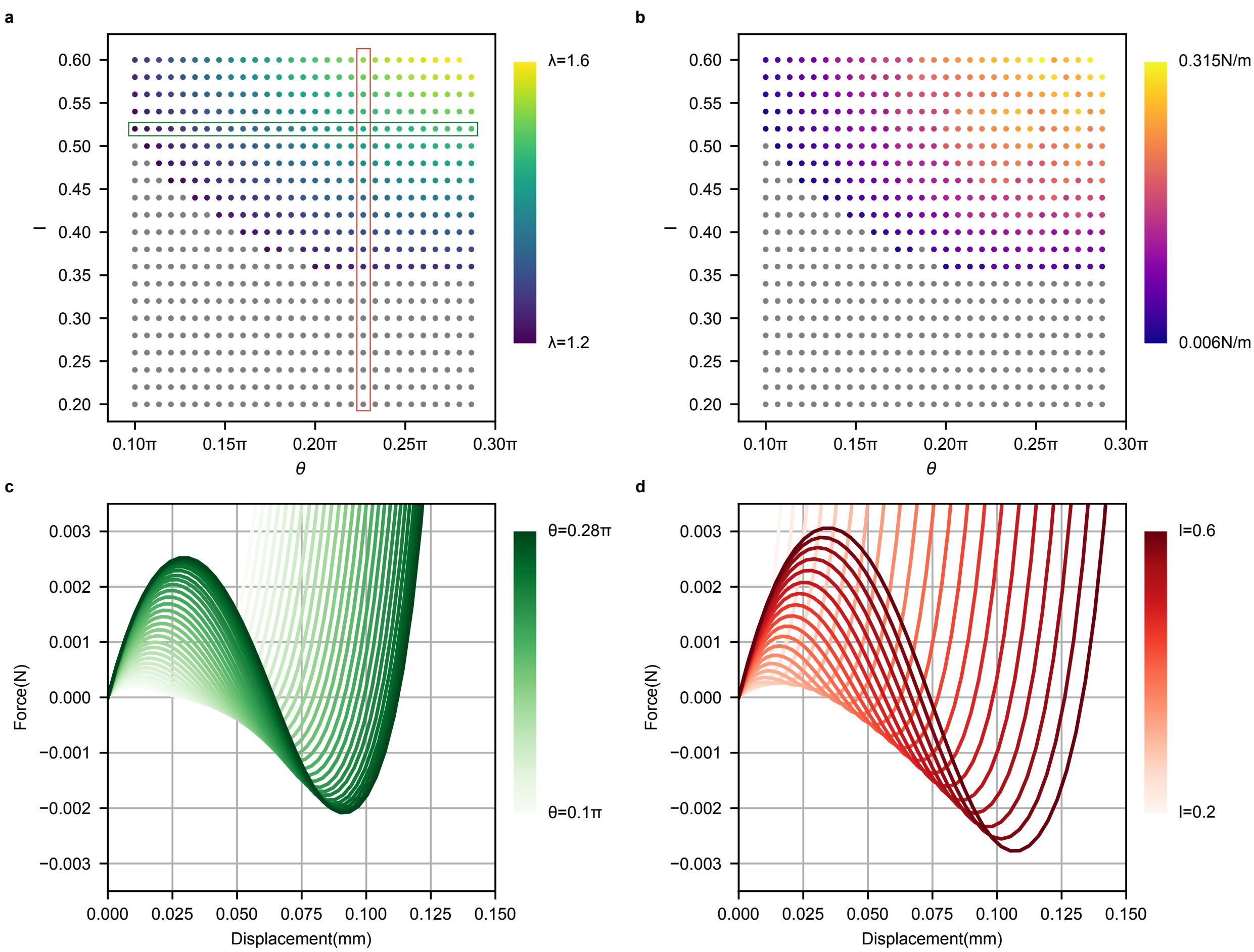

Supplementary Figure 5: **Parameter sweep of θ and l.** Two contour plots are generated from the simulation dataset: Contour map a) of stretch ratio λ, representing the expansion ratio at the second stable configuration; b) of tangent stiffness, defined as the slope of the stress-strain curve at the second stable configuration. Each data point corresponds to a single simulation. Force-displacement curves of unit cell subjected to uniaxial loading with parameters of c) $l = 0.52$, θ varies; and d) $\theta = 0.247\pi$, l varies. Source data are provided as a Source Data file.

Periodic boundary conditions (PBCs) are applied to the generated mesh. Mesh nodes along the left-right and top-bottom edges are identified and grouped in matched pairs. Equation of

constraints are then imposed to equate the displacements of each node pair, with relative motion controlled by four auxiliary reference points placed outside the unit cell domain. These reference points are later used as control handles for prescribed displacement loading. A node at the center of unit cell is pinned to eliminate global rigid body motions. A quasi-static, geometrically nonlinear analysis (Static Implicit) is performed to simulate uniaxial loading of the periodic tessellations. Displacement is prescribed at the reference points in y direction, the magnitude is 1.2 times the kinematically predicted second-equilibrium stretch.

We discuss the results from Finite Element (FE) analyses of two models featuring parametric combinations near the extreme ends of the λ spectrum, i.e., $(\theta, l) = (0.22\pi, 0.4)$ for $\lambda_{\text{min}} = 1.24$, and $(\theta, l) = (0.22\pi, 0.6)$ for $\lambda_{\text{max}} = 1.58$. The resulting stress-stretch curves of these unit cells exhibit classic bistability with two stable equilibria. The stretch at the second equilibrium equals to λ (Fig. 4a). The corresponding deformation exhibits isotropic expansion up to a prescribed $2\lambda L$ (Fig. 4b).

3.2 Parametric sweep through unit cell microstructures

A parametric study is conducted to evaluate the influence of unit cell geometry - specifically θ and l - on the mechanical behavior of the unit cell. For each combination of (θ, l), the unit cell is subjected to uniaxial loading, and the full stress–strain response is obtained. θ is swept from 0.1π to 0.28π and l is swept from 0.2 to $0.8/(\sin\theta + \cos\theta)$ with increments of 0.01π and 0.02 respectively.

From this, only the bistable parametric combinations are retained. This is defined by the presence of a second local strain energy minimum. We then calculate the tangent stiffness at this strain as it represents the stiffness of the unit cell once expanded. Both the isotropic expansion factor λ and the tangent stiffness at the second equilibrium state are plotted in Fig. 5a,b.

To isolate the effects of each geometric parameter, two representative sets of simulations are extracted with complete force-displacement curves: In the first set (Fig. 5c), θ is varied while l is fixed at 0.52. In the second set (Fig. 5d), l is varied while θ is fixed at 0.247π (or $\approx 45°$). In both, it is shown that by adjusting the parameters, we can tune the strain and maximize the tangent stiffness at the second equilibrium.

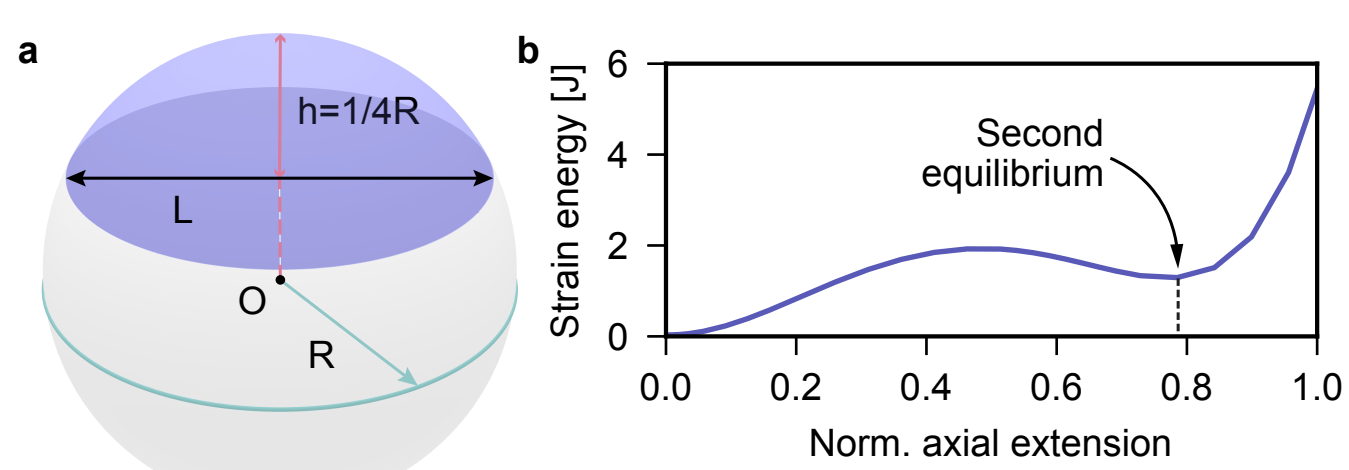

Supplementary Figure 6: **Geometry and deployment mechanics of the quarter spherical dome.** a) The target geometry is a section of a sphere of radius R. b) The strain energy versus degree of deployment. Source data are provided as a Source Data file.

3.3 Simulation of deployment and indentation of the dome

We simulate the deployment mechanics of the spherical cap (Fig. 6a) by locally expanding each unit cell in-plane to their respective second equilibrium state. The strain to reach this per unit cell has been calculated a priori above. This method of deployment simulation has no pre-knowledge of the actual target shape. Therefore, if the simulated deformed shape matches the 3D target geometry and is in a local strain energy minimum, we argue that the design is successful.

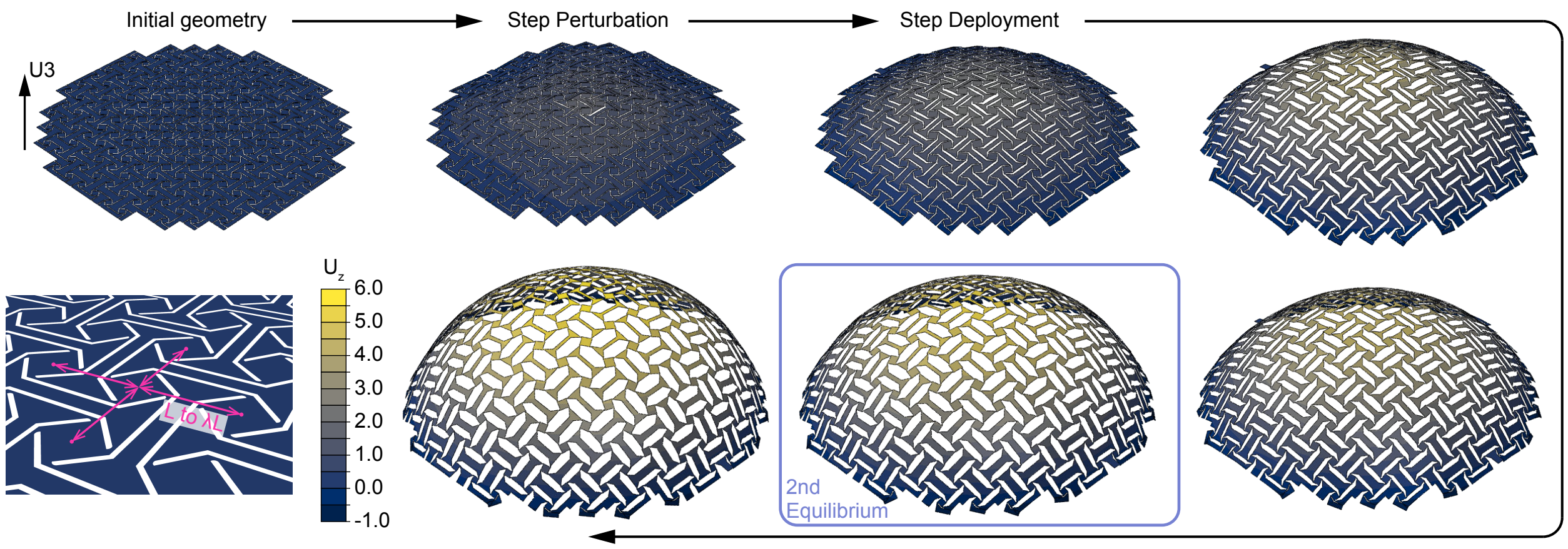

Supplementary Figure 7: **Finite Element prediction of the deformation sequence of deployment.**

Abaqus (Static Implicit, geometrically non-linear) is used. The initial geometry is generated using Rhinoceros3D and meshed using shell elements (S8R). To circumvent the instability inherent in each unit cell under force-controlled loading, we prescribe a local expansive displacement between every pair of neighboring unit cells from $2L$ to $2\lambda_i L$. λ_i is the average stretch of every neighboring pair of unit cells, i.e., each interior unit cell has four neighbors. This displacement is imposed as an axial connection element in Abaqus. To break symmetry about the $x-y$ plane, we introduce a slight perturbation in the z direction as the first loading step (Fig. 7).

The boundary conditions are set so that the four edges of the specimen connected to the polyimide membrane are confined in the z direction. Due to symmetry, the center point of the dome is fixed from moving in x and y.

The resulting simulation shows the correct deployed geometry as shown in Fig. 3 of the manuscript. The total strain energy per loading step (as a fraction between 0 and 1 of λ) shows a local minimum approximately 78% of the axial extension between unit cells (Fig. 6b). This is matched with experiments. Since the design algorithm does not account for the sign of the mean curvature, for more complex target geometries where some parts of the shape are convex and some are concave, the perturbation must be introduced with respect to the sign of the mean curvature.

We continue the deployment simulation using the FE model at the second equilibrium state to predict the behavior of the quarter dome under indentation. A hemispherical indenter ($E = 200\,\text{GPa}$) is generated atop the deployed dome. A downward displacement is applied to the sphere against the deployed dome up to 60% of the original height. Surface to surface non-penetrating contact condition is implemented. Fig. 8 shows the resulting von Mises stress state of the dome. The indenter is removed for better visualization.

Supplementary Note 4: Fabrication protocol

All fabrication steps are carried out in a cleanroom environment. We use silicon wafers as substrates for deposition (100 mm diameter, P-type, (100) orientation, 1-100 Ω cm resistivity, thickness 525 ± 25 µm; WAFERPRO LLC). The wafer is first treated with UV ozone for 15 min to grow a 500 nm thick layer of silicon dioxide (SiO_2) on the surface.

Silanization is performed by spin-coating the wafer with a 0.01% volume fraction solution of (3-Aminopropyl)triethoxysilane (APTES) at 4000 rpm. This surface treatment improves the adhesion

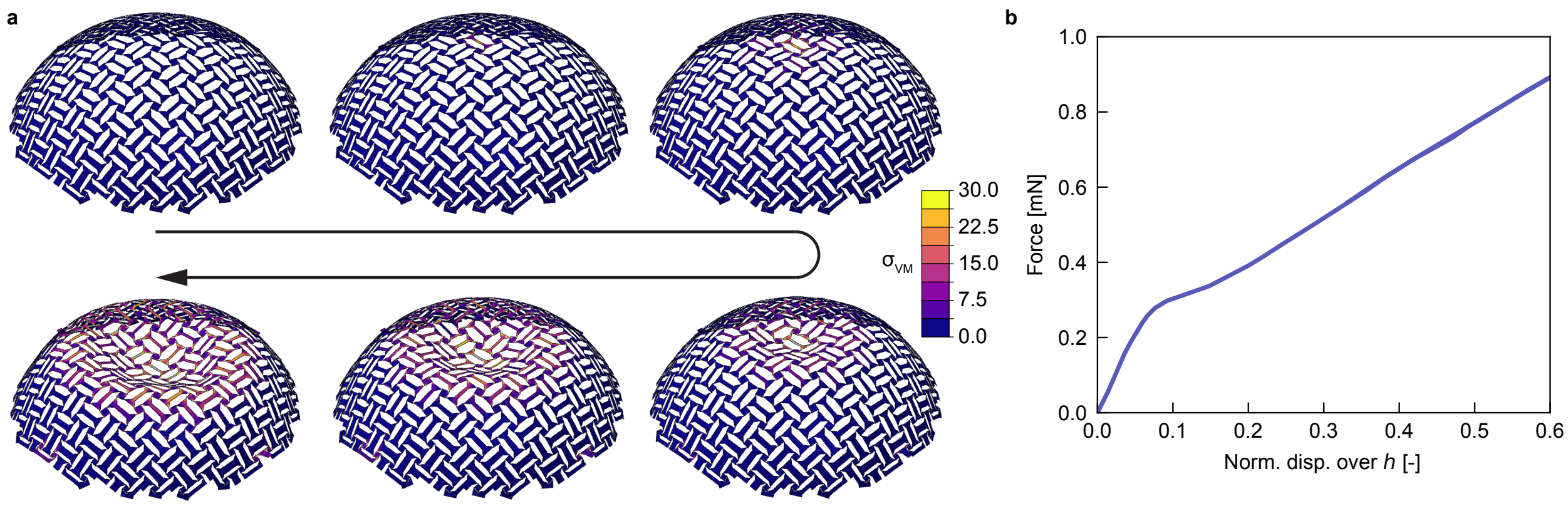

Supplementary Figure 8: **Finite Element prediction of the indentation of the deployed dome.** a) Simulated deformation sequence of the deployed dome during indentation loading, with the color map indicating the von Mises stress distribution. b) Simulated force–displacement response obtained from the indentation simulation, where the displacement is normalized by the dome height. Source data are provided as a Source Data file.

between the wafer and subsequent polyimide layers.

Following silanization, a 6 µm thick polyimide layer is deposited by spin-coating at 2000 rpm for 60 s. The wafer is then subjected to a multi-step soft- and hard-baking protocol: 90 °C for 90 s, 150 °C for another 90 s, followed by a temperature ramp to 300 °C at a rate of 5 °C min^{-1}. The temperature is held at 300 °C for 30 min, then cooled down to room temperature at the same rate. A second polyimide layer is deposited using the same procedure, resulting in a bilayer polyimide film.

Subsequently, a 12 µm thick sacrificial layer of AZ 12XT-20PL positive photoresist is spin-coated with a two-step spin profile: 500 rpm for 30 s followed by 2000 rpm for 60 s. The wafer is then baked at 100 °C for 3 min. Patterning is performed using a maskless lithography system (Bruker, formerly Rave NP, SF-100 Lightning). This system allows the use of digital masks directly exported as DXF files from Rhinoceros3D, enabling rapid design iteration and parameter adjustment.

After exposure, the wafer is baked at 90 °C for 1 min and developed in AZ 300MIF developer for 3 min to remove the exposed photoresist. The polyimide layer is then selectively etched using oxygen and SF_6 plasma in a reactive-ion etching system (RIE-180) for 40 min. During this step, approximately 75% of the top polyimide layer thickness is removed.

Before the top polyimide layer is completely etched through, it is gently peeled from the bottom polyimide layer at the edge. This step is done to avoid stress accumulation at the hinge regions within the unit cells. The peeled off polyimide film is placed on a spare wafer with the same side facing up and subjected to additional RIE for 15 min. The sample film is then flipped upside down and etched for another 3 min, ensuring full penetration through the slits on polyimide film without mechanically damaging the 2D structures. Finally, the sample is immersed in acetone for 5 min to fully dissolve any remaining photoresist, completing the fabrication process. This entire fabrication protocol requires approximately two days to complete. The method does not involve the use of strong acids or hazardous chemical baths.

4.1 Fabrication Evaluation

Two geometric parameters are particularly critical to the mechanical performance of the fabricated samples: the slit width s and the hinge width c, as defined in Supplementary Note 2.1. Deviations

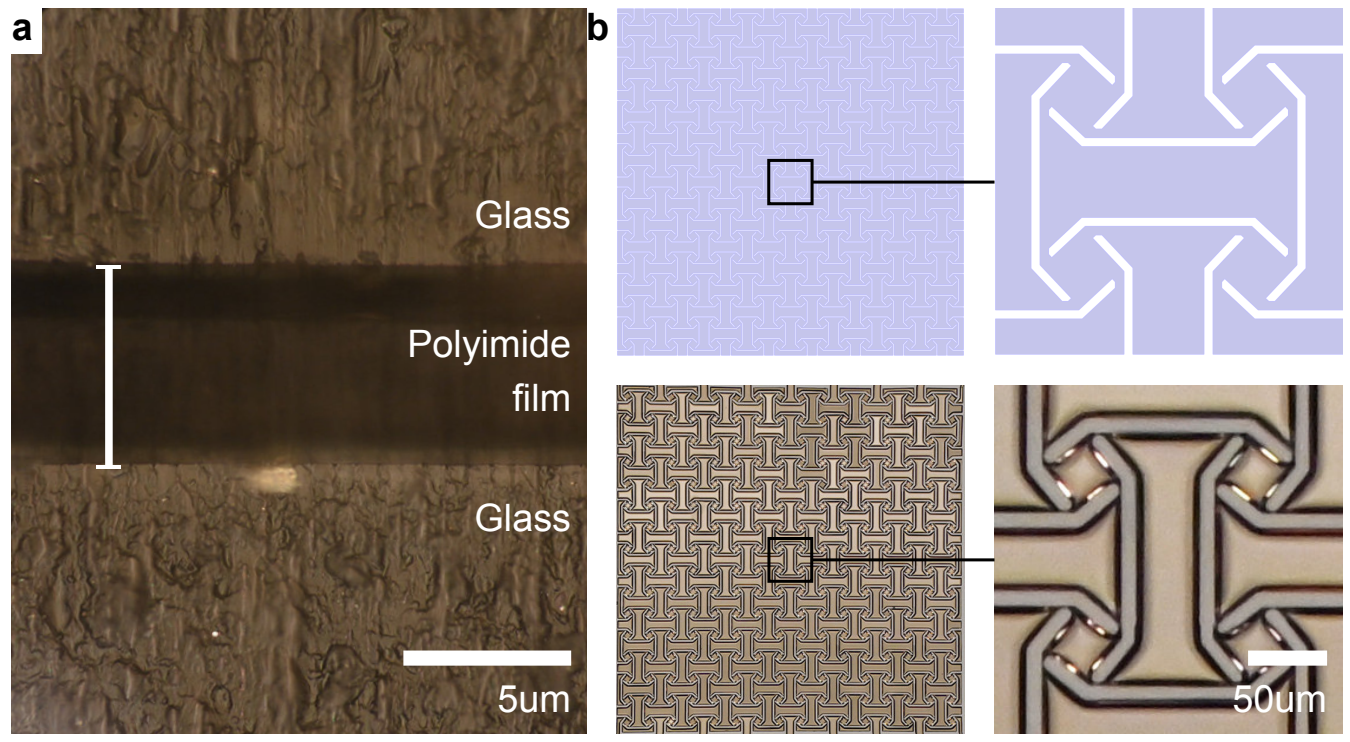

Supplementary Figure 9: **Dimensional accuracy of the fabricated specimens.** a) Microscopy image of polyimide thickness measurement. The Polyimide film is sandwiched between two glass slides. b) In-plane accuracy showing rounding of the corners and over etching of the hinges.

in these dimensions directly affect the bistable response and deployment behavior of the unit cells.

The polyimide film thickness is evaluated using an optical cross-section method. The free-standing film is placed between two glass slides, gently pressed to ensure close contact, and imaged under a 50× objective. A stage micrometer is used for pixel-to-length calibration. A baseline measurement of two glass slides without film is subtracted to eliminate systematic error. The measured polyimide thickness is 6.08(66) µm, consistent with the spin-coating protocol (Fig. 9a). The photoresist thickness is measured using a Filmetrics Profilm3D optical profilometer and found to be 12.41(16) µm (mean ± s.d., $n = 3$), consistent with the two-step spin profile described in Supplementary Note 4.

Deviations between designed and fabricated feature sizes arise from two primary sources. First, photoresist mask erosion and the partially isotropic component of fluorine-based RIE produce sloped sidewalls, resulting in a reverse-tapered cross-sectional profile that is narrower at the top and wider at the bottom. Second, for narrow openings, restricted transport of reactive species and ion flux leads to aspect-ratio-dependent etching (RIE lag), reducing the etch rate at the bottom of narrow features. Together, these effects systematically modify the dimensions of the slit width s and hinge width c. On average, s measures approximately 4 µm at the top layer and 8 µm at the bottom layer, while c measures approximately 45 µm and 60 µm at the top and bottom layers respectively.

To compensate for these systematic deviations, an inverse pattern correction is applied in the photomask design. The target values of s and c in the CAD file are set to 30 µm and 20 µm respectively, such that the fabricated structures achieve the intended dimensions of approximately 10 µm for s and 40 µm for c after processing. Furthermore, lithography and etching limitations prevent sharp corners from being faithfully reproduced; the fabricated slit tips exhibit rounding compared to the CAD design (Fig. 9b). To maintain consistency with experiments, the Finite Element models use the as-fabricated geometry.

Supplementary Note 5: Tension of the periodic structures

To characterize the mechanical behavior of the periodic samples (typical size ≈ 2 mm), a commercial tensile testing machine is found to be either cost-prohibitive or lacking the necessary sensitivity and compatibility for micro-scale displacement and force resolution, as well as real-time optical observation. A custom-built micro-tensile testing system is developed as shown in Fig. 10a.

The system consists of the load transducer mounted on a motorized stage via a 3D-printed

adapter. A fixed boundary is established using a right-angle bracket (AB90H, Thorlabs), secured with an M6 bolt on the opposite end. The bracket is position-adjustable to ensure proper alignment, enabling axial loading without off-axis forces.

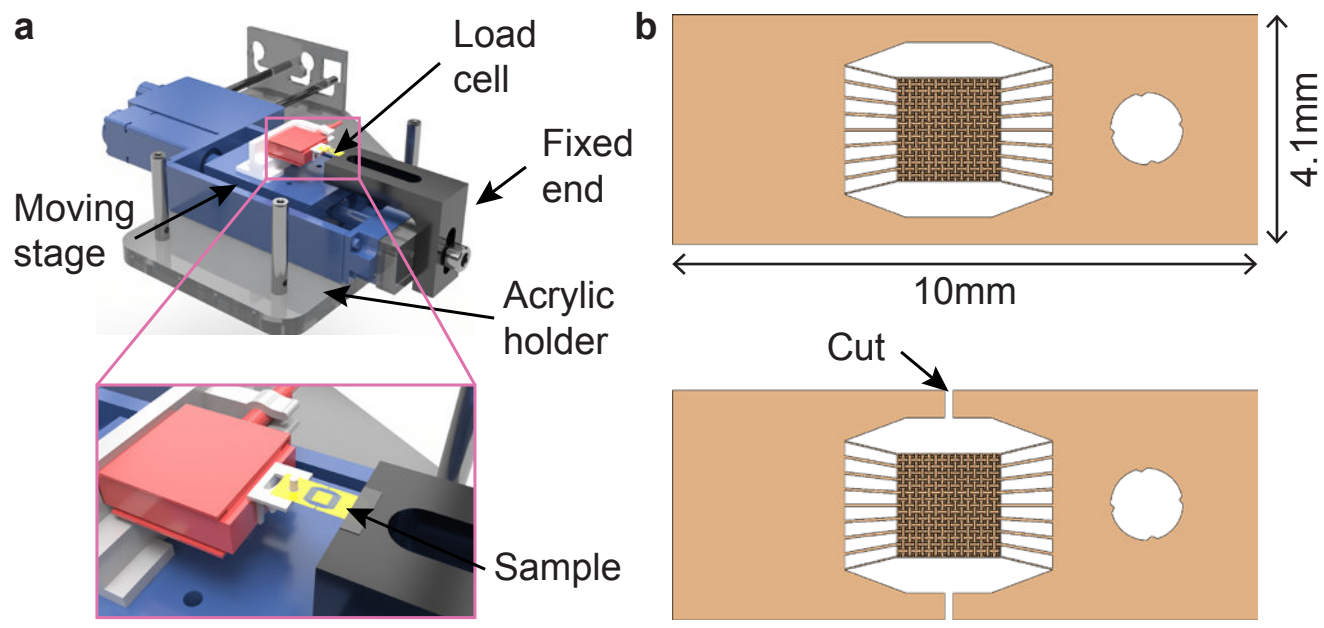

Supplementary Figure 10: **Mechanical testing setup and specimen shape.** a) The specimen is attached to a load transducer which is connected to a motorized micro-linear stage. b) Shape of the specimen as fabricated, and cut prior to testing.

The entire assembly is mounted onto a laser-cut acrylic base plate and supported vertically with optical posts. This configuration allows the entire system to be inverted and positioned directly over the observation area of an inverted optical microscope (Nikon Eclipse MA200 with DS-Fi3 Camera), enabling simultaneous force-displacement measurement and high-resolution imaging. The samples are subjected to a constant displacement rate of $5\,\mu m\,s^{-1}$ under ambient temperature.

A key advantage of this system lies in the separation of the mechanical testing and imaging modules, which allows for the entire setup to be transported and placed under different optical systems. This contrasts with in situ SEM-based testing stages, which are fixed in place and not easily adaptable for optical microscopy. The use of a 10 g capacity S-Beam Jr.® load cell (Futek) provides the necessary force sensitivity for meso-scale soft materials that are too large for nano-indentation systems and too small for standard mechanical testers. This effectively fills the gap between macro- and micro-scale mechanical characterization platforms.

The control interface integrates both the motion control of the Zaber linear stage and real-time data acquisition from the load cell. The graphical user interface includes functions such as overload protection, displacement and loading rate control, real-time data visualization, and data export. This flexible control architecture allows precise control of the testing process and facilitates repeatable experimental conditions.

The 8×8 unit cell sample is fabricated as shown in Fig. 10b. To approximate periodic boundary conditions during tensile testing, slanted arms connect the substrate to the unit cells along the leftmost and rightmost columns of the 8×8 unit cell array. These arms extend laterally beyond the square sample and are designed to accommodate in-plane deformation without constraining motion in the vertical (y) direction. To prevent damage during handling and mounting, the sample is initially fabricated with connecting bridges along the top and bottom edges to avoid pre-stretching the sample (Fig. 10b).

The samples (Fig. 10b) are placed on to the load transducer first. The linear stage then moves the specimens towards the fixed end, which is then affixed with tape once in position. Finally, the connecting bridges are cut, allowing stress to be applied to the specimen.

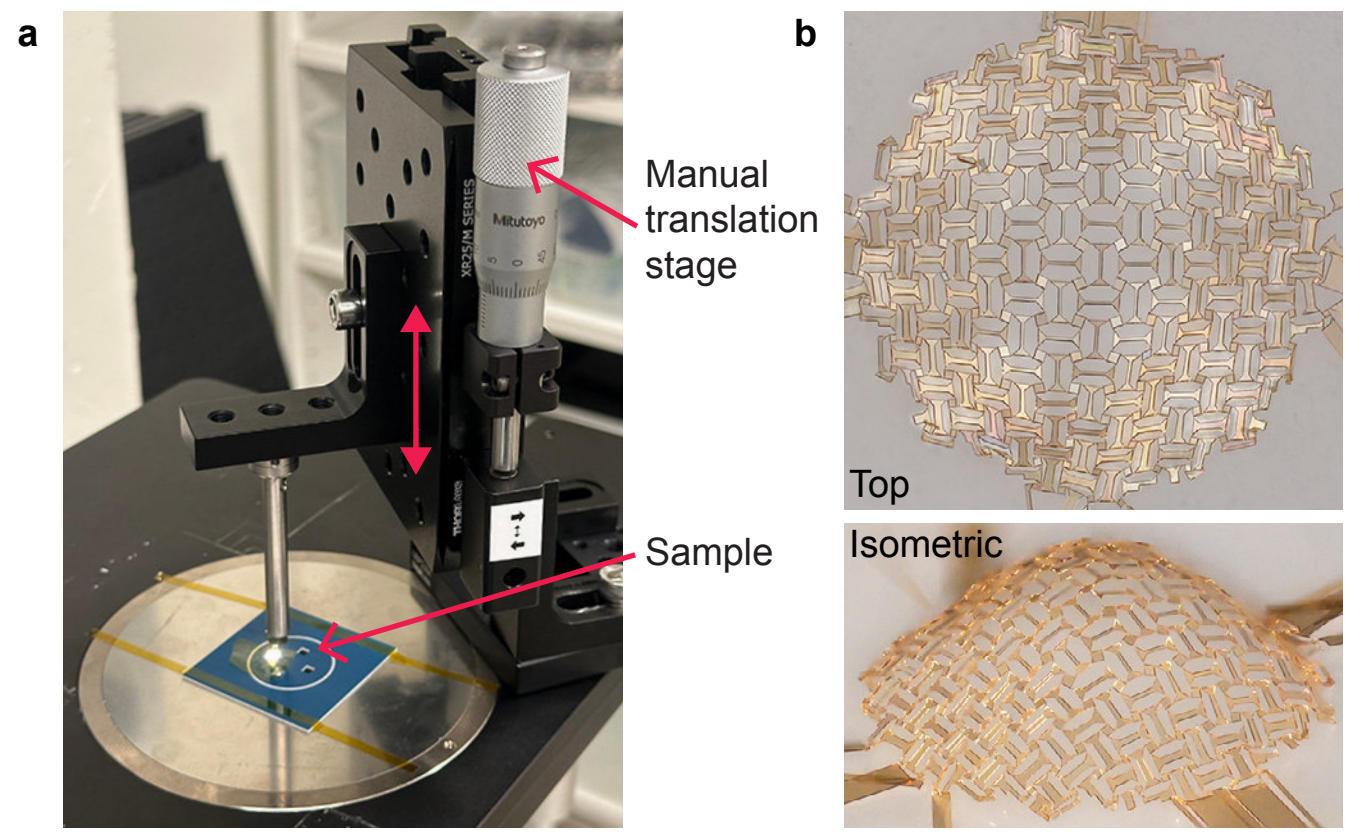

Supplementary Figure 11: **Mechanical deployment of the spherical cap.** a) Setup of dome deployment using a manual linear precision stage. b) Top and isometric view of free-standing deployed dome.

Supplementary Note 6: Spherical cap deployment and indentation

The deployment of the 3D dome structure is achieved by axially indenting the center using a 0.8 mm diameter spherical-shaped head. To facilitate in situ imaging during deployment, the movement of the indenter is oriented horizontally toward the microscope objective. As shown in Fig. 11a, the sample is flatly mounted on the observation stage (i.e., the movable platform above the microscope), while a linear translation stage is also fixed onto the same platform. The moving direction of the translation stage is aligned toward the microscope lens. During deployment, images are captured at every 0.1 mm increment of translation stage movement to record the deformation progression (see Supplementary Movie 2). The deployed dome is shown in Fig. 11b.

After full deployment, the dome structure is transferred onto the micro-mechanical testing stage. A 3D-printed indenter, designed as a cylindrical shaft with a hemispherical tip, is mounted onto the load transducer and advanced toward the dome to perform a single loading and unloading cycle. The indentation is conducted at a constant displacement rate of $0.01\,\mathrm{mm\,s^{-1}}$. The imaging setup is identical to that used for the two-dimensional sample mechanical tests, enabling synchronized acquisition of force-displacement data and optical visualization.

To reconstruct the deployed geometry in 3D, a series of images are taken at different focal planes, with the focal plane translated by 200 µm between consecutive images. A depth map is extracted from the image stack by identifying, for each pixel, the focal plane at which it appears sharpest — this focal plane position is assigned as the relative depth of that pixel, generating a 3D surface reconstruction of the deployed geometry. The vertex-wise deviation δ is visualized in Fig. 12. The central region shows close agreement with the target, while deviations up to $0.14R$ are observed at the rim, consistent with boundary-induced constraints on free expansion.

To demonstrate that plastic deformation is not the primary driver of shape deployment, we fabricate a two-dimensional sample of the same overall size but features a tessellation of identical unit cells for comparison. As shown in Fig. 13, using the same deployment method, the deployment of this uniform pattern does not form a dome but rather, a flat surface confined by boundary fixed points [6].

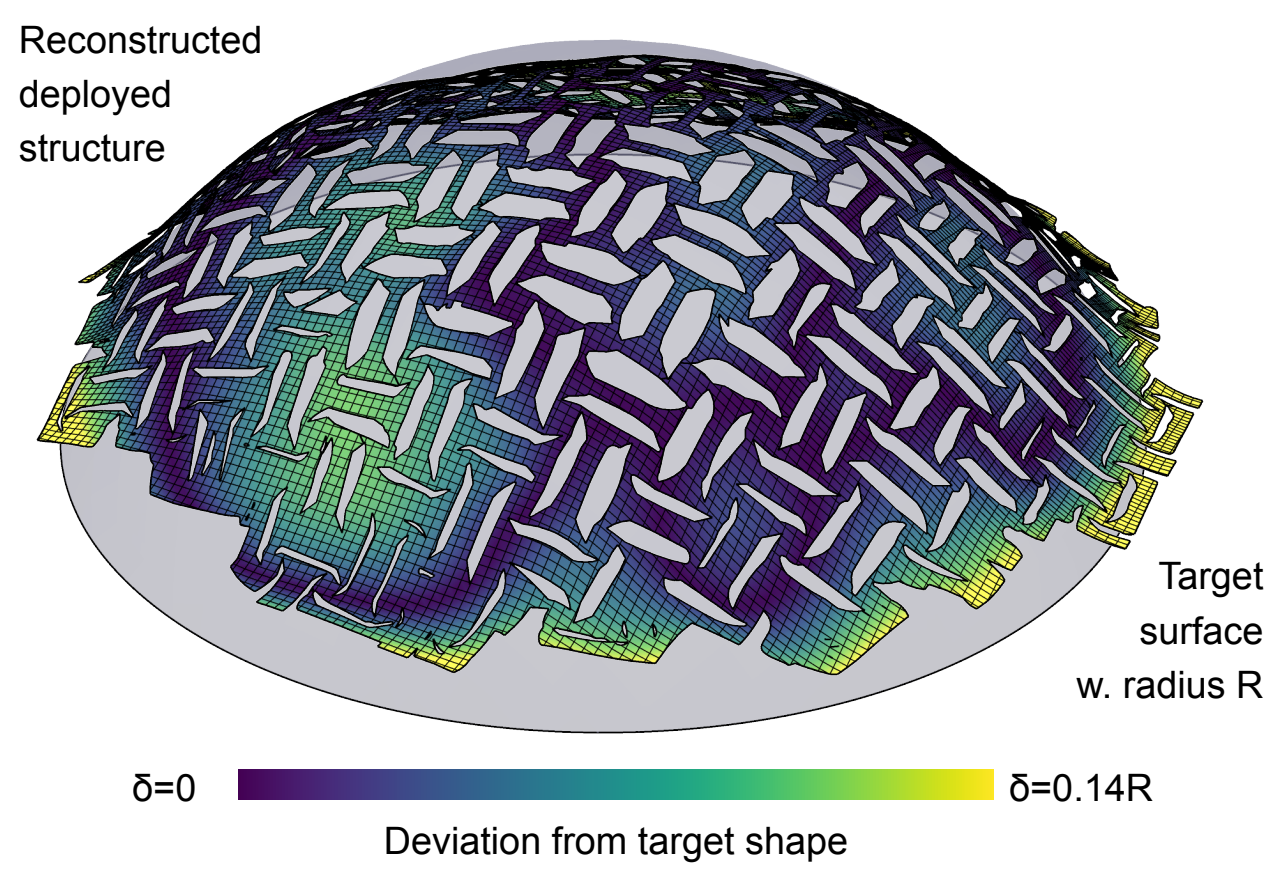

Supplementary Figure 12: **Geometric accuracy of the deployed dome structure.** The 3D reconstructed surface of a representative deployed dome (colored mesh) is superimposed onto the target spherical cap of radius R. The colormap indicates the pointwise deviation δ between the reconstructed and target surfaces, normalized by R.

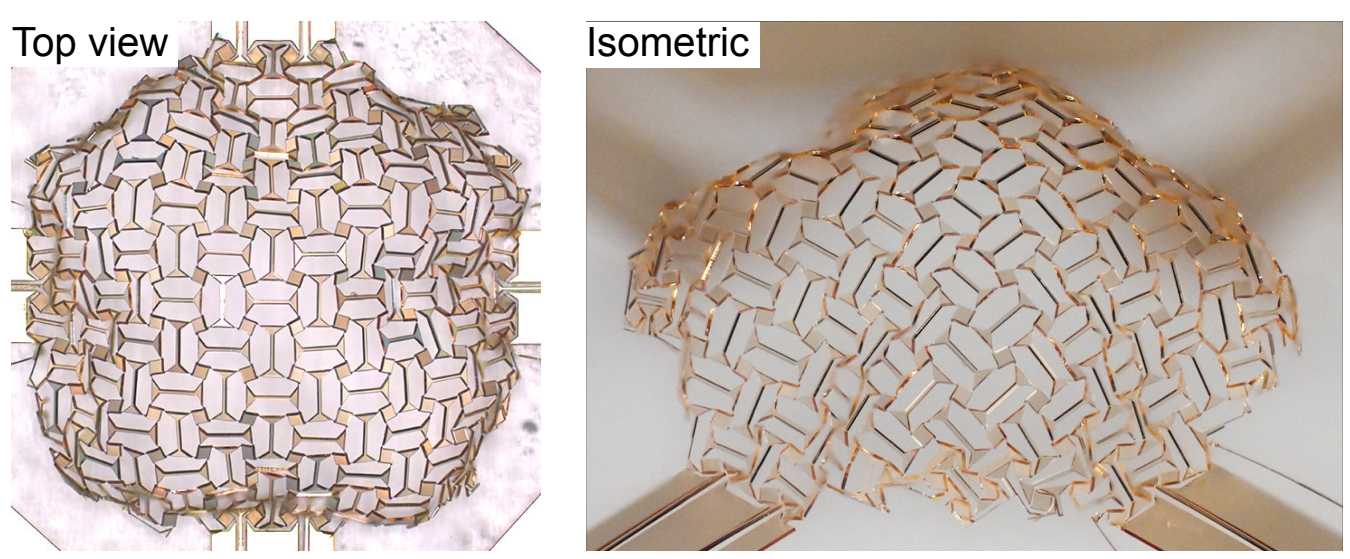

Supplementary Figure 13: **Deployment of a circular disk featuring a periodic tessellation.** Without the heterogeneity of the unit cell tessellation, a spherical shape is not achieved using the same mechanical indentation.

Supplementary Note 7: Design of a cornea device

As a representative application, we demonstrate the deployment of a cornea-shaped geometry that is shown in Fig. 4 of the manuscript. The target geometry approximates the anterior surface of a human cornea, modeled as a doubly curved dome sitting atop a simplified eye geometry (Fig. 14a). The conformal map of this target surface yields a spatially heterogeneous λ field ranging from 1.2 at the periphery to 1.6 at the center (Fig. 14b), reflecting the higher Gaussian curvature near the apex. The corresponding 2D precursor is fabricated on-wafer using the lithography protocol described in Supplementary Note 4 (Fig. 14c). After release from the substrate (Fig. 14d), the precursor is mechanically deployed onto a 3D-printed eye model from which the cornea has been removed, exposing the flat iris (Fig. 14e). The deployed structure conforms to the target cornea geometry and is mechanically stable without a supporting substrate. An inset reveals the spatially varying unit cell microstructure in the deployed state.

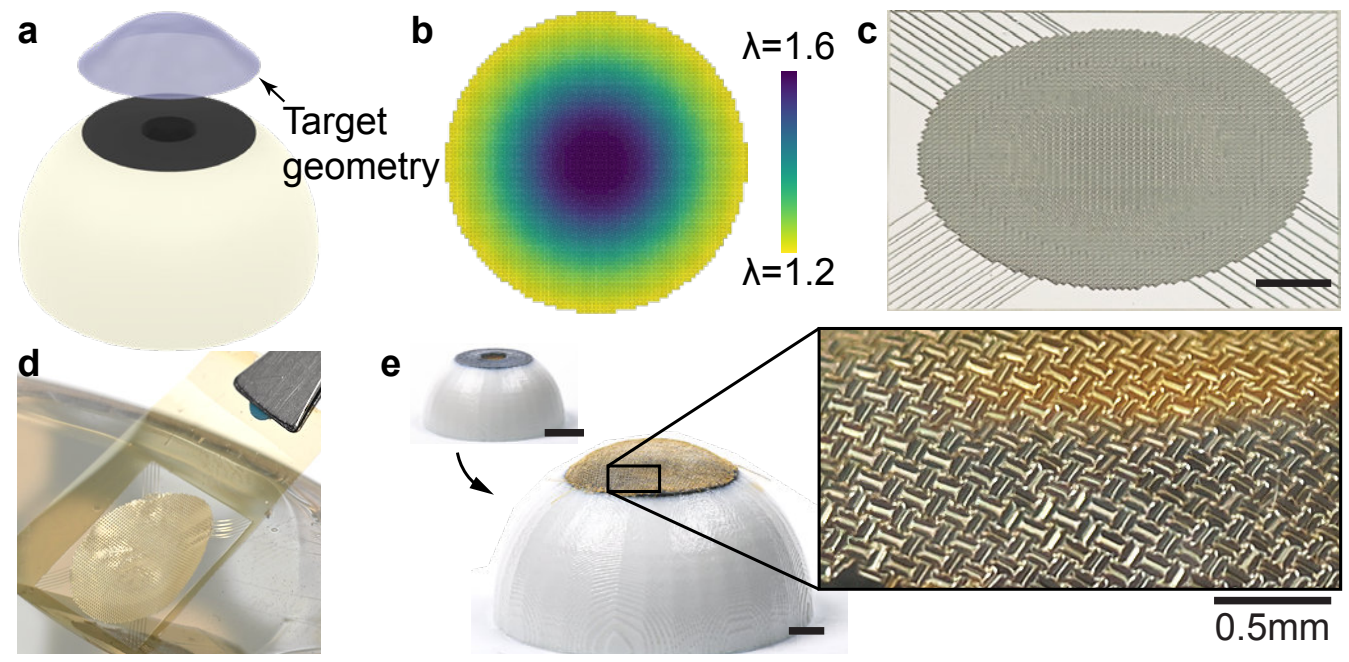

Supplementary Figure 14: **Cornea device design and fabrication.** a) Target cornea geometry shown atop a simplified eye model. b) Conformal map of the target surface onto the 2D plane, with the color spectrum indicating the local isotropic expansion factor λ per mesh face. c) Microscopy image of the fabricated 2D precursor on the wafer. d) Release of the patterned polyimide film from the substrate. e) Deployed cornea structure placed on a 3D-printed eye model with the cornea removed. Inset: magnified view of the deployed microstructure showing spatially varying unit cells. Scale bars, 5 mm.

Supplementary Note 8: Reflector design

The optical measurement setup is designed to observe the reflection behavior of the paraboloidal structure as shown in Fig. 15. A collimated laser (PL202, Thorlabs) with a beam diameter of 3 mm is directed onto the reflective surface of the deployed paraboloid. The position of the reflector sample is adjusted using two manual linear stages (Thorlabs). All components are mounted on a single optical breadboard.

To capture the reflected pattern, a 300×300 mm white imaging plane is placed in between the laser and the paraboloidal reflector, at a distance $D = 40$ mm from the reflector. A 3 mm diameter bore is laser-cut at the center of the board to allow the laser beam to pass through and reach the reflector while minimizing scattering.

The rays reflect from the reflector surface, converge at the designed focal length before diverging onto the imaging plane, forming a visible pattern. This pattern is captured using a digital camera (Nikon D780 with an 85 mm lens) positioned perpendicular to the board. All measurements are conducted in a darkroom environment with constant laser power. Camera settings are kept identical across all trials to maintain consistent lighting conditions for comparative analysis. This setup enables precise measurement of the reflected spot size and intensity distribution, which directly correlate with the curvature and surface quality of the fabricated reflector.

8.1 Intensity calculation

We derive the expected intensity distribution on a planar observation screen for a finite-sized paraboloidal reflector under collimated illumination using geometric optics and energy conservation.

Geometry. We consider a paraboloid of revolution with vertex at $z = 0$, opening in the $+z$ direction, described in cylindrical coordinates (ρ, ϕ, z) by

$$z(\rho) = \frac{\rho^2}{4f}, \tag{15}$$

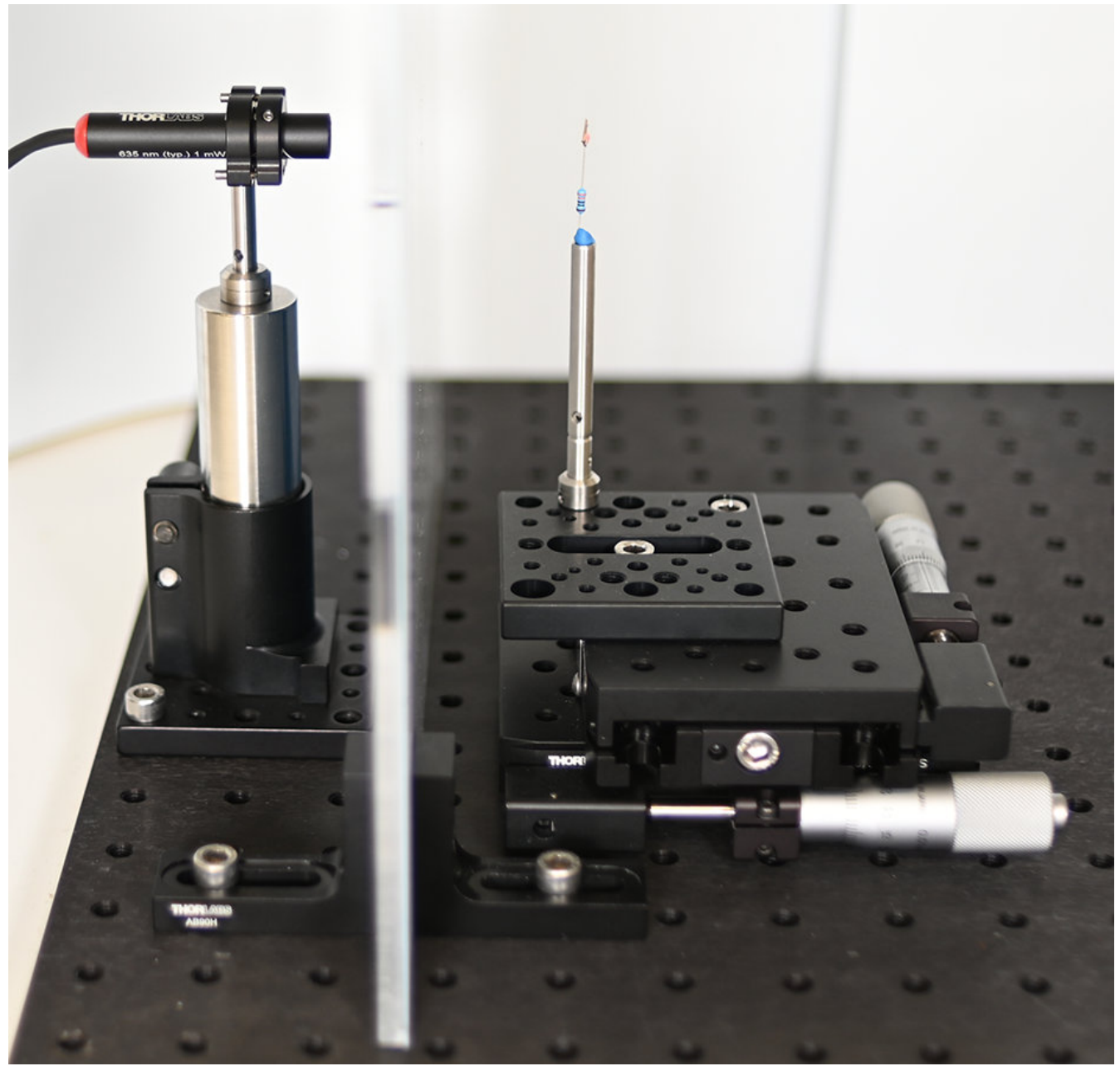

Supplementary Figure 15: **Setup of the reflector experiments.** A visible light laser is positioned to the left of the imaging plane. The deployed paraboloidal reflector is on the right. A small hole on the imaging plane allows the laser to reach the reflector which then reflects the laser onto the plane.

where f is the focal length. The reflector is finite, with aperture radius r and depth $H = z(r)$, which fixes the focal length as

$$f = \frac{r^2}{4H}. \tag{16}$$

A uniform, collimated beam of incident irradiance I_0 propagates along the optical axis in the $-z$ direction and illuminates the entire aperture. The reflector is assumed to be perfectly specular and lossless.

An observation plane perpendicular to the optical axis is placed at $z = D$, with $D > f$, i.e., downstream of the focal point so that the reflected rays have crossed and diverged.

Ray mapping. Due to axial symmetry, each ray is uniquely labeled by its radial coordinate ρ on the reflector. After specular reflection, the ray intersects the observation plane at a radial coordinate R, giving a one-to-one mapping

$$R = R(\rho). \tag{17}$$

For a paraboloidal reflector under on-axis collimated illumination, this mapping can be written in closed form as

$$R(\rho) = \frac{2(D - f)\,(\rho/2f)}{1 - (\rho/2f)^2}. \tag{18}$$

Because the reflector is finite, rays only originate from $0 \le \rho \le r$. Consequently, the reflected pattern on the observation plane has finite support,

$$0 \le R \le R_{\max}, \tag{19}$$

where $R_{\max} = R(\rho = r)$ is the radius reached by the ray reflected from the rim of the reflector. No reflected light exists for $R > R_{\max}$.

Energy conservation and intensity distribution. The incident illumination is uniform, so the optical power contained in an annular element of the reflector between ρ and $\rho + d\rho$ is

$$dP = I_0\, 2\pi\rho\, d\rho. \tag{20}$$

After reflection, this same power is distributed over an annulus on the observation plane between R and $R + dR$, with area $2\pi R\, dR$. By definition of the irradiance $I(R)$ on the observation plane,

$$dP = I(R)\, 2\pi R\, dR. \tag{21}$$

Equating the two expressions and using the ray mapping $R(\rho)$ yields

$$I(R) = I_0\, \frac{\rho}{R} \left| \frac{d\rho}{dR} \right|. \tag{22}$$

Carrying out this differentiation for the paraboloidal geometry gives the closed-form expression

$$I(R) = I_0\, \frac{f^2}{(D-f)^2}\, \frac{\left[1 - u(R)^2\right]^3}{1 + u(R)^2}, \tag{23}$$

where

$$u(R) = \frac{-A + \sqrt{A^2 + 4R^2}}{2R}, \qquad A = 2(D - f). \tag{24}$$

Equation (23) is valid for $0 \leq R \leq R_{\max}$ and $I(R) = 0$ for $R > R_{\max}$.

Power conservation. Integrating the irradiance over the observation plane recovers the total incident power,

$$\int_0^{R_{\max}} I(R)\, 2\pi R\, dR = I_0\, \pi r^2, \tag{25}$$

confirming energy conservation for a finite aperture and a lossless reflector.

Relation to numerical ray tracing. In the numerical model used in Fig. 5, each reflective square element of the deployed structure is treated as a locally flat mirror, and one ray per element is traced according to the law of reflection. Each ray carries equal optical power, so the simulated intensity distribution is obtained by counting ray intersections on the observation plane and normalizing by the area of concentric annuli.

Supplementary Note 9: Scaling of bending stiffness

As compared to macroscale deployable structures, the effect of gravity is lessened in smaller specimens. We provide a simple analysis of this effect. The bending stiffness of a unit cell under gravity can be expressed as EI, where E is the Young's modulus of the material, and I is the second moment of area of the unit cell's cross-section. For a rectangular cross-section with width b and height h, the second moment of area is given by $I = \frac{1}{12}bh^3$. If the unit cell is scaled down uniformly in all three dimensions by a factor of $k \in (0, 1)$, then the new second moment of area becomes,

$$I' = \frac{1}{12} b'(h')^3 = \frac{1}{12}(kb)(kh)^3 = k^4 I, \tag{26}$$

and the new bending stiffness is then $EI' = k^4 EI$. The self-weight per unit length under gravity scales with cross-sectional area:

$$w \sim \rho g b h \quad \Rightarrow \quad w' \sim \rho g k b \cdot k h = k^2 w \tag{27}$$

Assuming the unit cell behaves like a simply supported beam, the central deflection under self-weight is proportional to:

$$\delta \sim \frac{wL^4}{EI} \tag{28}$$

After scaling:

$$\delta' \sim \frac{(k^2 w)(k^4 L^4)}{k^4 EI} = k^2 \cdot \delta \tag{29}$$

When a unit cell is uniformly scaled down by a factor k, its bending stiffness decreases as k^4, but the self-weight per unit length decreases as k^2. As a result, the gravitational deflection also decreases as k^2. Therefore, although the unit cell becomes mechanically more compliant, its deflection under gravity is reduced, making the scaled-down structure relatively stiffer under self-weight.

Supplementary References

[1] Zihao Dong, Qipei He, Dawei Shen, Zheng Gong, Deyuan Zhang, Wenqiang Zhang, Takahito Ono, and Yonggang Jiang. Microfabrication of functional polyimide films and microstructures for flexible mems applications. *Microsystems & Nanoengineering*, 9(1):31, 2023.

[2] Ahmad Rafsanjani and Damiano Pasini. Bistable auxetic mechanical metamaterials inspired by ancient geometric motifs. *Extreme Mechanics Letters*, 9:291–296, 2016.

[3] Yue Wang, Yingying Ren, and Tian Chen. From Kirigami to Hydrogels: A Tutorial on Designing Conformally Transformable Surfaces. *Journal of Applied Mechanics*, 90(044801), 2022.

[4] Wikipedia contributors. Stereographic projection — Wikipedia, the free encyclopedia, 2024. [Online; accessed 17-February-2026].

[5] Rohan Sawhney and Keenan Crane. Boundary First Flattening. *ACM Trans. Graph.*, 37(1):5:1–5:14, 2017.

[6] Changyeob Baek, Andrew O Sageman-Furnas, Mohammad K Jawed, and Pedro M Reis. Form finding in elastic gridshells. *Proceedings of the National Academy of Sciences*, 115(1):75–80, 2018.
