# Supplementary material for: Deployable 3D architectures from wafer-fabricated precursors
Source: Nat Commun. 2026 Jul 25;17:9047. doi: 10.1038/s41467-026-76052-y (PMC13507144; doi:10.1038/s41467-026-76052-y)
Supplement: Supplementary file 2 [file 41467_2026_76052_MOESM2_ESM.pdf]

nature portfolio

Peer Review File

Deployable 3D architectures from wafer-fabricated precursors

Corresponding Author: Dr Tian Chen

This file contains all reviewer reports in order by version, followed by all author rebuttals in order by version.

Version 0:

Reviewer comments:

Reviewer #1

(Remarks to the Author)
Deployable 3D architectures from wafer-fabricated precursors

In this manuscript, the authors present a method to transform wafer-fabricated, two-dimensional precursors into free-standing three-dimensional architectures through a mechanical stimulus. This is achieved by combining conformal mapping with multi-step lithographic fabrication and a bistable auxetic unit-cell design featuring a tunable second equilibrium strain.

Overall, the study appears original and of clear interest to the readership of Nature Communications. The methodology is well explained, and both the experimental demonstrations and numerical simulations appear sound, supported by the supplementary materials. However, I have several suggestions for revision to further strengthen the manuscript:

1. The authors mention in the introduction that 3D printing via two-photon polymerization offers geometric freedom but is severely limited in its ability to integrate dissimilar materials and components. However, the references cited in this context are relatively old. Recent advances in two-photon polymerization are well presented in the perspective article https://onlinelibrary.wiley.com/doi/10.1002/lpor.202301312 and should be considered by the authors. This article, among other aspects, presents studies on the transformation of 3D structures onto various complex substrates. The original methodology is described in https://doi.org/10.1002/adfm.202214409 and has been further employed for the realization of metasurfaces on non-planar substrates (https://www.degruyterbrill.com/document/doi/10.1515/nanoph-2022-0667/html), which can be combined with https://advanced.onlinelibrary.wiley.com/doi/10.1002/admt.202402137 and multimaterial printing approaches such as https://onlinelibrary.wiley.com/doi/full/10.1002/smtd.202401809. The authors should improve their discussion of the novelty of their method in comparison with the current state of the art in two-photon polymerization

2. The fabrication process is described in the supplementary file but could be further supported by data characterizing film thicknesses and the precision of the lithography process. In this context, the authors should investigate deviations between the designed and fabricated feature sizes.

3. To further demonstrate the originality of the presented manufacturing approach, the authors should investigate the repeatability and accuracy of the deployment process using the dome structure, including its dependence on the mechanical properties of the structure.

4. The dome structures presented in Fig. 3 and Video 2 do not fully recover after indentation. The authors should present damage characteristics of the structure after indentation and discuss potential damage mechanisms in relation to the deployment approach, taking into account the aspects mentioned above.

5. In the final section of the manuscript, the authors investigate the use of the deployed 3D structures as paraboloidal reflectors. However, in the current presentation it is difficult to understand the correlation between the experimental results and the simulations. The authors should explain and discuss the discrepancies between the calculated pattern and the experimental validation. Additionally, it remains unclear how the authors determined the reported value of 25% of the total reflected intensity for each case in their experiments.

6. The quality of the figures should be improved to better support the written text. In many cases the visual information is

barely recognizable. This appears to be due to unnecessary details that do not add value to the understanding of the manuscript (e.g., Fig. 1). A thorough revision of the figures is recommended.

(Remarks on code availability)

Reviewer #2

(Remarks to the Author)
This work proposes a wafer-fabricated deployable 3D architectures. A 2D precursor composed of bistable auxetic units is designed from a target conformal stretch field, doubly-curved shapes. The manuscript presents a deployed geometry and an optical demonstration based on a paraboloidal reflector. The concept is interesting, but this manuscript does not meet the journal's standards the manuscript.

Overall, the figures lack polish; their quality is below the standard expected for this journal.

-The abstract is too long. It should be revised to concisely state what the paper discusses. In particular, the first 2–3 sentences appear unnecessary.

- The Introduction does not clearly state the scope or questions the manuscript intends to address.
- Is the paper focused on a manufacturing (wafer-fabricated process) or on a structural design/properties enabled by the kirigami architecture?

- The literature review is insufficient for the topics in this manuscript.
- Two-photon polymerization (TPP) is mentioned early, but I think that there is no relationship between "wafer-fabrication" in this manuscript and the TPP.

-The information in Fig. S3 is very unclear. While the Jacobian matrix and the formulas seem to follow a parabolic-equation form, Equations (5), (6), (7), and (8) are not presented in a way that allows easy verification.

-Overall, the figures are too small and have poor readability. In Fig. 1, the conceptual explanation is insufficient, making it very difficult to understand the idea from the figure alone; appropriate explanatory text should be added.
-In Fig. 2(b), the conditions under which the reported color spectrum was obtained are not specified; likewise, the manuscript seems not to state under what conditions the discrete conformal map was computed.
-In Fig. 1(c), the concept of multi-step lithography is not conveyed at all.
-In Fig. 1(e), it is unclear why two hemispherical images are shown; these appear to be deployed patterns, but if so, the definition of the unit design is not evident.
-Additionally, in Fig. 1, Fig. 2, and Fig. 4s, panels (a–d) use black backgrounds, whereas the remaining figures use white backgrounds.
-In Fig. 2(d), the units for stretch appear to be missing.
In short, the figure styles are not unified, and adherence to the journal's presentation standards is questionable.

-Fig. 2(a) shows the auxetic pattern studied in this work. However, many auxetic patterns are possible, and the manuscript does not adequately justify why this particular pattern was chosen.

- for Figures S1 and S8, figures contain two sub-figures (a) and (b), but the captions does not any descriptions of these subfigures.

- For Section S2.4, the manuscript states that the generative algorithm was implemented in Python and visualized using Rhino with the Grasshopper plugin.
- Please specify the software versions and, for Rhino in particular, the relevant conditions used for visualization.
- For the finite-element simulations, which version of Abaqus was used?

- Fig. 4 demonstrates an impressive variety of deployed 3D structures. Nevertheless, the paper ultimately appears to target spherical geometries for application to the cornea. In that light, Fig. 4 does not seem to support the core application. It would be more appropriate to present structures that faithfully represent the spherical eye shape.
-Also, "Target 3D shape" would be better phrased as "Target 3D design."

- The manuscript presents a "paraboloidal reflector" as an application of the fabricated structures. The necessity of using the proposed structure specifically for a paraboloidal reflector is not established.
- The experiments provided are insufficient to substantiate the unique advantages of the proposed design.

- The title of this manuscript is included "wafer-fabricated," the work reads as manufacturing-centric. If the primary claim lies in wafer-scale fabrication, the manuscript should include the various evaluation about the quality of fabrication (accuracy, fabrication yield, and durability).

(Remarks on code availability)
This work proposes a wafer-fabricated deployable 3D architectures. A 2D precursor composed of bistable auxetic units is designed from a target conformal stretch field, doubly-curved shapes. The manuscript presents a deployed geometry and an optical demonstration based on a paraboloidal reflector. The concept is interesting, but this manuscript does not meet the journal's standards the manuscript.

Version 1:

Reviewer comments:

Reviewer #1

(Remarks to the Author)
The authors have addressed the reviewers' comments comprehensively, and the manuscript has improved accordingly. I recommend its publication in the present form.

(Remarks on code availability)

Reviewer #2

(Remarks to the Author)
The first version of the manuscript had several weaknesses and many unclear points. However, in this revised version, the authors have responded sincerely and thoroughly to all of my comments. I have carefully reviewed the revised manuscript. The first version of manuscript did not fully meet the journal's standard, but, now I think that the authors have adequately addressed the major concerns. In my opinion, the manuscript is now suitable for publication in Nature Communications. Therefore, I recommend acceptance.

(Remarks on code availability)

Open Access This Peer Review File is licensed under a Creative Commons Attribution 4.0 International License, which permits use, sharing, adaptation, distribution and reproduction in any medium or format, as long as you give appropriate credit to the original author(s) and the source, provide a link to the Creative Commons license, and indicate if changes were made.
In cases where reviewers are anonymous, credit should be given to 'Anonymous Referee' and the source.
The images or other third party material in this Peer Review File are included in the article's Creative Commons license, unless indicated otherwise in a credit line to the material. If material is not included in the article's Creative Commons license and your intended use is not permitted by statutory regulation or exceeds the permitted use, you will need to obtain permission directly from the copyright holder.
To view a copy of this license, visit https://creativecommons.org/licenses/by/4.0/

Response to the reviewers' comments of manuscript:

Deployable 3D architectures from wafer-fabricated precursors
by
Yue Wang, Kelvin Shum, Yuyang Song, Tian Chen

Please refer to the "diff. version" appended with our resubmission for a detailed account of all the changes, corrections and additions to the revised version of the manuscript. The revised/new text is highlighted in **red**.

In the following, we address each of the Reviewers' comments/suggestions. The numbering of the comments (e.g., R1C1, R1C2, ..., R2C1, R2C2,...) is added by us to aid in cross-referencing our responses. The rebuttals are blue whereas the changes to the manuscript that we have replicated in the rebuttal are highlighted in **red**.

Reviewer 1

In this manuscript, the authors present a method to transform wafer-fabricated, two-dimensional precursors into free-standing three-dimensional architectures through a mechanical stimulus. This is achieved by combining conformal mapping with multi-step lithographic fabrication and a bistable auxetic unit-cell design featuring a tunable second equilibrium strain.

Overall, the study appears original and of clear interest to the readership of Nature Communications. The methodology is well explained, and both the experimental demonstrations and numerical simulations appear sound, supported by the supplementary materials. However, I have several suggestions for revision to further strengthen the manuscript:

We thank the reviewer for the positive and thoughtful assessment of our manuscript. We appreciate the recognition of the originality of the proposed approach, the clarity of the methodology, and the consistency between the experimental demonstrations and numerical simulations. We are grateful for the reviewer's constructive suggestions, which have helped us to further clarify the scope, positioning, and limitations of the work. We address each comment in detail below and believe that the resulting revisions have strengthened the manuscript.

R1C1: The authors mention in the introduction that 3D printing via two-photon polymerization offers geometric freedom but is severely limited in its ability to integrate dissimilar materials and components. However, the references cited in this context are relatively old. Recent advances in two-photon polymerization are well presented in the perspective article https://onlinelibrary.wiley.com/doi/10.1002/lpor.202301312 and should be considered by the authors. This article, among other aspects, presents studies on the transformation of 3D structures onto various complex

substrates. The original methodology is described in https://doi.org/10.1002/adfm.202214409 and has been further employed for the realization of metasurfaces on non-planar substrates (Highly conformable terahertz metasurface absorbers via two-photon polymerization on polymeric ultra-thin films), which can be combined with https://advanced.onlinelibrary.wiley.com/doi/10.1002/admt.202402137 and multimaterial printing approaches such as https://onlinelibrary.wiley.com/doi/full/10.1002/smtd.202401809. The authors should improve their discussion of the novelty of their method in comparison with the current state of the art in two-photon polymerization.

We thank the reviewer for highlighting recent advances in multiphoton lithography (MPL) and two-photon polymerization. We agree that these approaches have significantly expanded the capabilities of direct three-dimensional microfabrication, including fabrication on non-planar substrates, transfer-based strategies, and emerging multimaterial demonstrations. We have revised the introduction to acknowledge these developments and added the suggested references.

Crucially, we view MPL and the approach presented here as complementary routes toward the shared long-term goal of digital electronics in three dimensions. MPL advances this goal through serial, voxel-level geometric freedom and localized material patterning. Our approach advances an alternative route: mechanically programmed, bistable deployment from standard planar wafer fabrication — where parallel processing, yield, and heterogeneous material integration are already mature. We treat deployment as an integral step of the manufacturing process; the planar precursor is an intermediate product and the deployed 3D structure is the final device.

We have revised the introduction to frame this distinction clearly; serial, resist-based 3D writing versus parallel, wafer-scale planar fabrication followed by mechanically programmed deployment; while emphasizing complementarity rather than competition. The revised text and new references are reproduced below.

The following are the texts we have included in the introduction:

Current efforts to realize three-dimensional electronic and photonic systems follow two distinct fabrication paradigms: direct three-dimensional microfabrication, and planar wafer fabrication followed by transformation or assembly into three-dimensional forms [4-6]. Recent advances in multiphoton lithography (MPL), including two-photon polymerization, have significantly expanded the capabilities of the former, enabling freeform 3D architectures, writing on non-planar substrates, and a range of hybrid and transfer-based integration strategies [7-10].

With the shared long-term goal of digital electronics in 3D, MPL advances this objective by offering geometric freedom and localized material patterning through serial, voxel-by-voxel writing [11]. This serial nature, together with the reliance on material-specific photoresists and sequential processing steps, represents a fundamental distinction from wafer-scale fabrication and can pose challenges for large-area scalability, throughput, and heterogeneous integration within established semiconductor workflows [12,13].

Here we ask: can standard planar wafer fabrication be used to manufacture precursors that deploy uniquely, stably, and accurately to prescribed doubly-curved 3D geometries without a supporting substrate? We address this question by combining conformal mapping, bistable auxetic microstructure design, and multi-step lithography into a unified fabrication and deployment pipeline. This approach leverages existing planar wafer fabrication, where parallel processing, yield, and heterogeneous material integration are already mature, and combines it with mechanically programmed, bistable deployment to achieve prescribed three-dimensional architectures. We treat the 2D-to-3D deployment as an integral step of the manufacturing process: the planar precursor fabricated on the wafer is an intermediate product, and the deployed 3D structure is the final manufactured device [14-16].

References:

[4] Song, Z. et al. Kirigami-based stretchable lithium-ion batteries. Scientific reports 5, 10988 (2015).
[5] Guo, X. et al. Controlled mechanical assembly of complex 3D mesostruc- tures and strain sensors by tensile buckling. npj Flexible Electronics 2, 14 (2018).
[6] Zhang, X., Zhang, X., Duan, Y., Zhang, L. & Ni, X. All-optical geometric image transformations enabled by ultrathin metasurfaces. Nature communications 14, 8374 (2023).
[7] den Hoed, F. M. et al. Facile handling of 3d two-photon polymerized microstructures by ultra-conformable freestanding polymeric membranes. Advanced Functional Materials 33, 2214409 (2023).
[8] Zyla, G. & Farsari, M. Frontiers of laser-based 3d printing: a perspective on multi-photon lithography. Laser & Photonics Reviews 18, 2301312 (2024).
[9] Zyla, G. et al. Film-based multi- photon lithography for efficient printing of electromagnetic surface structures. Advanced Materials Technologies 10, 2402137 (2025).
[10] Arriaga-D´avila, J. et al. From sin- gle to multi-material 3d printing of glass-ceramics for micro-optics. Small Methods 2401809 (2025).
[11] Gu, S. et al. 3d nanolithography with metalens arrays and spatially adaptive illumination. Nature 648, 591–599 (2025).
[12] Kawata, S., Sun, H.-B., Tanaka, T. & Takada, K. Finer features for functional microdevices. Nature 412, 697–698 (2001).
[13] Gansel, J. K. et al. Gold helix photonic metamaterial as broadband circular polarizer. Science 325, 1513–1515 (2009).
[14] Xu, S. et al. Assembly of micro/nanomaterials into complex, three- dimensional architectures by com- pressive buckling. Science 347, 154– 159 (2015).
[15] Choi, G. P. T., Dudte, L. H. & Mahadevan, L. Programming shape using kirigami tessellations. Nature Materials 18, 999–1004 (2019).
[16] Sim, K. et al. Three-dimensional curvy electronics created using conformal additive stamp printing. Nature Electronics 2, 471–479 (2019).

R1C2: The fabrication process is described in the supplementary file but could be further supported by data characterizing film thicknesses and the precision of the lithography process. In this context, the authors should investigate deviations between the designed and fabricated feature sizes.

We thank the reviewer for this suggestion. We have added a dedicated subsection (SI Sec. 4.1) to characterize the fabrication process quantitatively. Specifically, we report the measured thicknesses of both the polyimide film and the photoresist layer, and compare the designed versus fabricated feature sizes of the geometric parameters s (slit width) and c (hinge width).

The polyimide film thickness is measured using an optical cross-section method, in which the freestanding film is sandwiched between two glass slides and imaged under a 50x objective. The measured thickness is $6.08 \pm 0.66\ \mu m$, consistent with the spin-coating protocol. The photoresist thickness is measured using a Filmetrics Profilm3D profilometer and found to be $12.41 \pm 0.16\ \mu m$ (mean $\pm$ s.d., $n = 3$).

Regarding feature size accuracy, deviations between designed and fabricated dimensions arise from two primary sources: photoresist mask erosion and the partially isotropic component of fluorine-based RIE, which together produce sloped sidewalls and a reverse-tapered cross-sectional profile, *i.e.*, narrower at the top and wider at the bottom. In addition, aspect-ratio-dependent etching (RIE lag) further modifies narrow features. As a result, the slit width s measures approximately 4 μm at the top and 8 μm at the bottom, while the hinge width c measures approximately 45 μm and 60 μm at the top and bottom respectively.

To compensate for these systematic deviations, we apply an inverse pattern correction in the photomask design: the target values of s and c in the CAD file are set to 30 μm and 20 μm respectively, such that the fabricated structures achieve the intended values of approximately 10 μm for s and 40 μm for c. Additionally, because lithography and etching limitations prevent sharp corners from being faithfully reproduced, the slit tips exhibit rounding in the fabricated structures. The finite element simulations are modeled using the as-fabricated geometry to maintain consistency between simulation and experiment. These additions are reflected in the revised SI Sec. 4.1 and the corresponding figure (below).

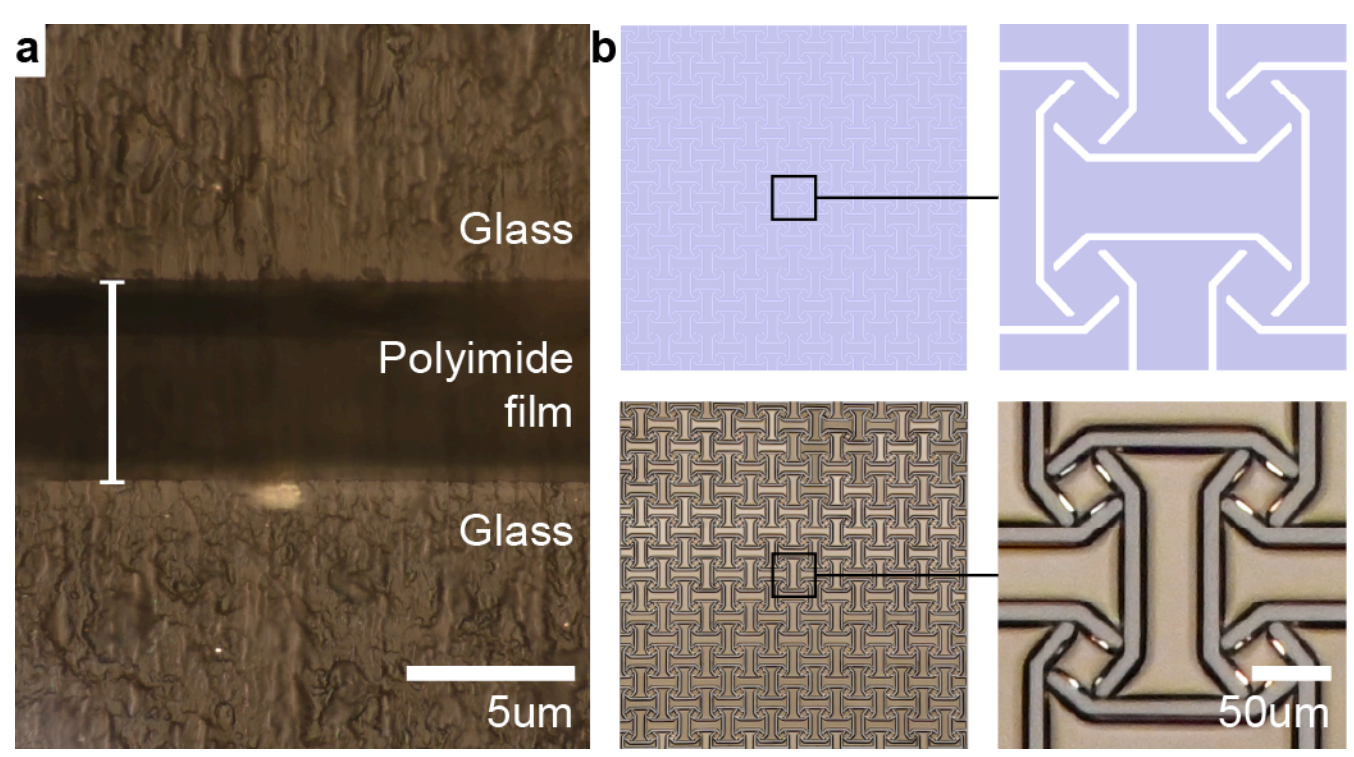

Figure R1 [SI Fig. 8] **Dimensional accuracy of the fabricated specimens.** a) Microscopy image of polyimide thickness measurement. The polyimide film is sandwiched between two glass slides. b) In-plane accuracy showing rounding of the corners, and over etching of the hinge.

We have updated SI Sec. 4.1 as follows,

Two geometric parameters are particularly critical to the mechanical performance of the fabricated samples: the slit width s and the hinge width c, as defined in SI Sec. 2.1. Deviations in these dimensions directly affect the bistable response and deployment behavior of the unit cells.

The polyimide film thickness is evaluated using an optical cross-section method. The freestanding film is placed between two glass slides, gently pressed to ensure close contact, and imaged under a 50x objective. A stage micrometer is used for pixel-to-length calibration. A baseline measurement of two glass slides without film is subtracted to eliminate systematic error. The measured polyimide thickness is $6.08 \pm 0.66\ \mu m$, consistent with the spin-coating protocol (Fig. 8a). The photoresist thickness is measured using a Filmetrics Profilm3D optical profilometer and found to be $12.41 \pm 0.16\ \mu m$ (mean $\pm$ s.d., $n = 3$), consistent with the two-step spin profile described in SI Sec. 4.
Deviations between designed and fabricated feature sizes arise from two primary sources. First, photoresist mask erosion and the partially isotropic component of fluorine-based RIE produce sloped sidewalls, resulting in a reverse-tapered cross-sectional profile that is narrower at the top and wider at the bottom. Second, for narrow openings, restricted transport of reactive species and ion flux leads to aspect-ratio-dependent etching (RIE lag), reducing the etch rate at the bottom of narrow features. Together, these effects systematically modify the dimensions of the slit width s and hinge width c. On average, s measures approximately $4\ \mu m$ at the top layer and $8 \mu m$ at the bottom layer, while c measures approximately $45\ \mu m$ and $60\ \mu m$ at the top and bottom layers respectively.

To compensate for these systematic deviations, an inverse pattern correction is applied in the photomask design. The target values of s and c in the CAD file are set to $30\ \mu m$ and $20\ \mu m$ respectively, such that the fabricated structures achieve the intended dimensions of approximately $10\ \mu m$ for s and $40\ \mu m$ for c after processing. Furthermore, lithography and etching limitations prevent sharp corners from being faithfully reproduced; the fabricated slit tips exhibit rounding compared to the CAD design (Fig. 8b). To maintain consistency with experiments, the finite element models use the as-fabricated geometry.

R1C3: To further demonstrate the originality of the presented manufacturing approach, the authors should investigate the repeatability and accuracy of the deployment process using the dome structure, including its dependence on the mechanical properties of the structure.

We thank the reviewer for this valuable suggestion. We agree that demonstrating the repeatability and geometric accuracy of the deployed dome is important to further support the originality and robustness of the proposed manufacturing approach. A unique aspect of our system is that the deployment process is enabled by mechanical bistability. The bistable transition cannot be reversed without introducing damage, so a direct repeatability assessment requires the fabrication and deployment of multiple nominally identical samples rather than repeated deployment cycles on a single specimen.

To address this comment, we have fabricated and deployed three dome precursors following the same multi-step wafer fabrication procedure described in the manuscript. The dome height and lateral span are measured for each sample from calibrated side-view photographs taken under a fixed camera configuration. The measured dome heights are 0.671±0.018mm and lateral spans are 2.29±0.13mm (mean ± s.d.), against target values of 0.675mm and 2.7mm respectively. The low standard deviations confirm consistent and repeatable deployment behavior across all samples. The systematic underestimation of the span (15%) is consistent across all samples and is attributed to the boundary conditions of the physical structure, which constrain free expansion at the edge points rather than to variability in material properties or fabrication quality.

To quantify the geometric accuracy of the deployed shape, we performed a 3D surface reconstruction of a representative deployed dome using focal-plane stacking and compared it pointwise to the target hemispherical geometry. The vertex-wise deviation δ between the reconstructed and target surfaces is visualized as a colormap in SI Fig. 11. The central region of the deployed structure shows close agreement with the target geometry ($\delta \approx 0$), while the largest deviations ($\delta \leq 0.14R$) are concentrated at the rim, consistent with the boundary-induced constraints on free expansion. This spatial pattern of deviation is observed consistently across all deployed samples.

The deployment accuracy and repeatability are therefore primarily governed by the geometric design and boundary conditions rather than by variability in material properties across samples. The consistency of the rim deviation across all samples confirms that the deployment process is robust and reproducible, and that deviations from the target geometry are systematic and predictable rather than stochastic.

These results, including the colormap deviation figure and the statistical summary of dome height across samples, have been added to the revised Supplementary Information.

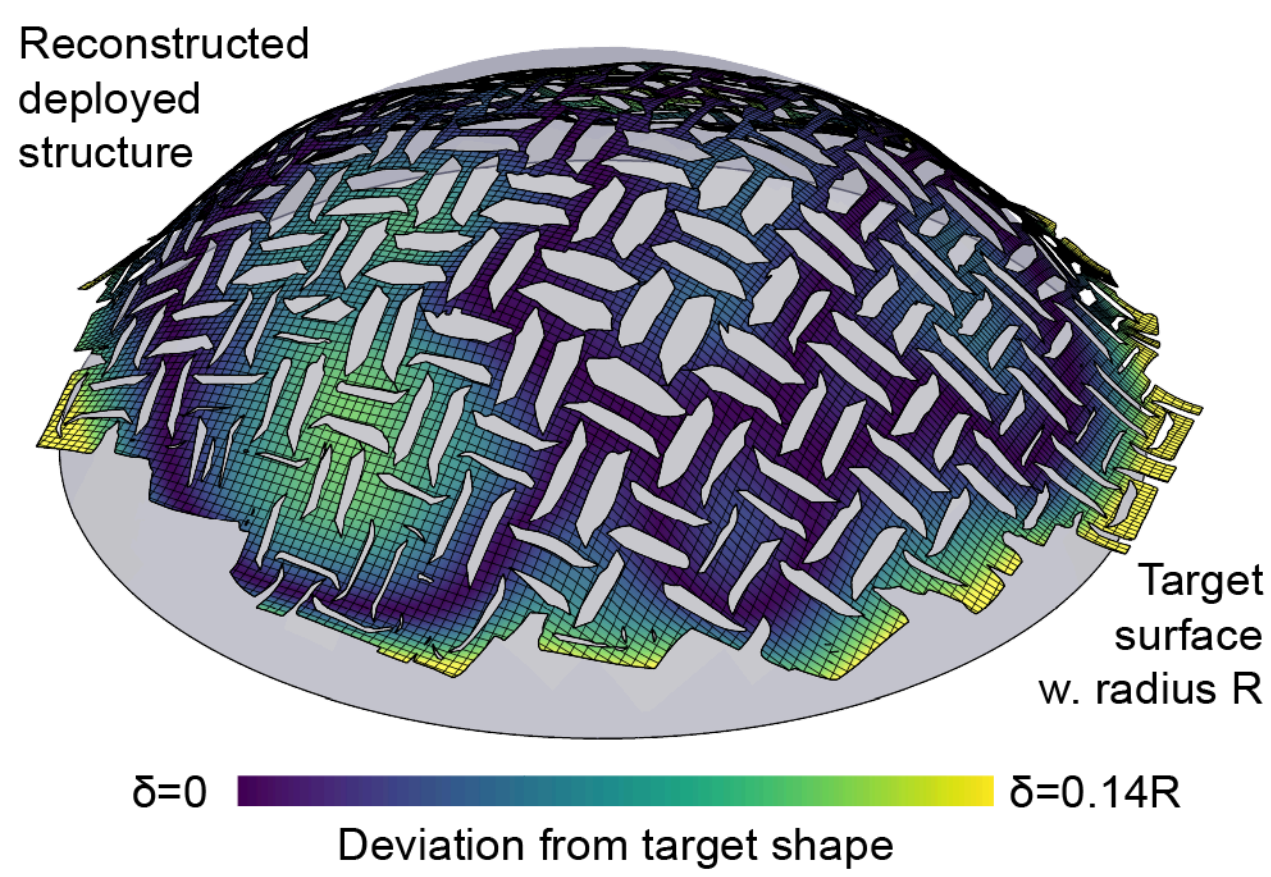

Figure R2. [SI Fig. 11] **Geometric accuracy of the deployed dome structure.** The 3D reconstructed surface of a representative deployed dome (colored mesh) is superimposed onto the target hemispherical surface of radius R. The colormap indicates the pointwise deviation δ between the reconstructed and target surfaces, normalized by R.

This is added to the SI:

To reconstruct the deployed geometry in 3D, a series of images are taken at different focal planes, with the focal plane translated by$200\mu m$ between consecutive images. A depth map is extracted from the image stack by identifying, for each pixel, the focal plane at which it appears sharpest --- this focal plane position is assigned as the relative depth of that pixel, generating a 3D surface reconstruction of the deployed geometry. The vertex-wise deviation δ is visualized in Fig. 11. The central region shows close agreement with the target, while deviations up to 0.14R are observed at the rim, consistent with boundary-induced constraints on free expansion.

This is modified in the manuscript:

To assess the repeatability of the deployment process, three dome structures are fabricated and deployed following the same procedure. The measured dome heights are 0.671±0.018mm and lateral spans are

2.29±0.13mm (mean±s.d.) respectively. This confirms consistent and repeatable deployment behavior across samples. The systematic underestimation of the span (target: 2.70mm, under estimated by 15%) is attributed to the boundary conditions of the physical structure, which constrain free expansion at the edge points. To quantify geometric accuracy, a 3D surface reconstruction of a representative deployed dome is generated using focal-plane stacking (see SI Sec. 6). The pointwise geometric deviation between the reconstructed surface and the target hemisphere is mapped in SI Fig. 11, confirming that deviations are concentrated at the rim ($\delta \leq 0.14R$), with the central region closely matching the target geometry.

R1C4: The dome structures presented in Fig. 3 and Video 2 do not fully recover after indentation. The authors should present damage characteristics of the structure after indentation and discuss potential damage mechanisms in relation to the deployment approach, taking into account the aspects mentioned above.

We thank the reviewer for this observation. The incomplete shape recovery observed after indentation is primarily attributed to localized damage within the hinge regions of the unit-cell microstructure after significant indentation (at 60% of the original height), rather than to the deployment mechanism itself. Specifically, the effective hinge thickness and width in the fabricated samples are smaller than their nominal design values due to lithographic resolution limits and etching non-uniformities. This geometric deviation leads to stress concentrations under indentation, resulting in plasticity in the hinges. Post-indentation microscopic inspection further reveals localized hinge buckling. These local instabilities reduce the effective energy barrier between the two stable states of the bistable auxetic unit cells, thereby hindering complete shape recovery upon unloading.

Importantly, this damage does not originate from the deployment process itself. Deployment follows a prescribed, predominantly in-plane deformation pathway designed to bring each unit cell to its second stable equilibrium. In contrast, indentation introduces off-path, localized bending and compressive stresses that are not present during deployment. The resulting degradation is therefore local rather than global, and does not indicate catastrophic failure or loss of structural integrity of the deployed architecture. Future iterations will co-optimize hinge geometry and material selection to improve post-deployment mechanical robustness.

We have included the following in the manuscript:

We note that the dome does not fully recover its original shape after significant indentation (Fig. 3d). This partial irreversibility is attributed to localized damage in the hinge regions of the unit-cell microstructure under off-path loading conditions. Specifically, indentation introduces localized bending and compressive stresses that are absent during deployment, leading to plastic yielding and buckling of hinge elements. Importantly, this behavior does not originate from the deployment process itself, which follows a prescribed deformation pathway to the second stable equilibrium.

R1C5: In the final section of the manuscript, the authors investigate the use of the deployed 3D structures as paraboloidal reflectors. However, in the current presentation it is difficult to understand the correlation between the experimental results and the simulations. The authors should explain and discuss the discrepancies between the calculated pattern and the experimental validation. Additionally, it remains

unclear how the authors determined the reported value of 25% of the total reflected intensity for each case in their experiments.

We thank the reviewer for this suggestion. We have revised the paraboloidal reflector section to clarify the relationship between simulations and experiments and to explain the sources of discrepancy.

To address the reviewer's concern of predicting the reflected patterns, we employ two complementary geometric optics models. First, we treat the reflector as a smooth continuous parabolic surface and analytically derive the intensity profile as a function of radial distance from the center. This continuous analysis confirms that the reflected intensity decays rapidly away from the center, explaining why the experimentally measured spatial extent of the reflected light appears smaller than the predicted geometric boundary. Second, recognizing that the deployed structure contains many voids, we numerically trace one ray per rotating square within each bistable unit cell, where each square is assumed to be locally flat and perfectly reflective. Each red dot in (manuscript's new) Fig. 5d therefore represents the intersection of a single reflected ray with the imaging plane. This discrete ray tracing captures the characteristic shape and spatial distribution of the reflected pattern, including the diagonal features arising from the square unit-cell architecture. Details of both models are now provided in SI Sec. 7.1.

Regarding the R25% metric previously reported, we have removed it from the revised manuscript. A direct quantitative comparison of reflected intensity distributions is complicated by the fact that the discrete model accounts only for reflections from the rotating squares, whereas the fabricated reflector includes additional reflective surfaces between unit cells, making intensity-based normalization inconsistent between simulation and experiment. The comparison is therefore intended to validate curvature control and focal behavior rather than exact point-wise intensity agreement, and we have clarified this explicitly in the revised manuscript.

We have included the following in the manuscript:

To predict the reflected patterns, we employ a ray-based geometric optics model using the known parameters f, r and D. First, we consider the reflector as a smooth parabolic surface, and analytically derive the intensity profile as a function of the radial distance from the center. This continuous analysis confirms that the reflected intensity decays rapidly away from the center, explaining why the experimentally measured spatial extent of the reflected light appears smaller than the predicted geometric boundary. Recognizing that the deployed structure contains many voids, we additionally model the reflections from only the rotating squares within each bistable unit cell. Each square is assumed to be locally flat, and one ray per square is traced according to the local surface normal and projected onto the imaging plane. Each red dot in Fig. 5d therefore represents the intersection of a single reflected ray with the imaging plane. This discrete ray tracing captures the characteristic shape and spatial distribution of the reflected pattern, including the diagonal features arising from the square unit-cell architecture. As expected for paraboloidal geometries, reflected rays converge at the designed focal length before diverging toward the imaging plane (see SI Sec. 7.1 for details).

In both continuous and discrete numerical approximations, the predicted ray distributions show a strong accumulation of reflected rays near the center for all three focal lengths, with the intensity decaying rapidly outward. From the discrete ray tracing, the predicted boundary radii of the illuminated regions on

the imaging plane are R=69.5mm, 147mm, and 381mm respectively. Experimentally, reflected patterns are recorded under identical illumination conditions for all samples (Fig. 5e). The measured spatial extent of the reflected light is smaller than these predicted boundaries, consistent with the rapid intensity decay confirmed by the continuous model --- little measurable light reaches the geometric boundary of the pattern. For the shortest focal length case f=0.45r, the illuminated region exceeds the size of the imaging plane.

R1C6: The quality of the figures should be improved to better support the written text. In many cases the visual information is barely recognizable. This appears to be due to unnecessary details that do not add value to the understanding of the manuscript (e.g., Fig. 1). A thorough revision of the figures is recommended.

We have thoroughly revised all the figures in the manuscript. In particular, Fig. 1 has been completely redesigned. The previous version mixed photographs, renders, and schematics with insufficient conceptual explanation. The new Fig. 1 uses the annulus geometry as a representative example and presents the complete pipeline as a clean conceptual figure with six panels: a) target geometry, b) conformal mapping with λ colorfield and unit cell grid, c) parametrized unit cell design, d) maskless lithography schematic, e) release from substrate, and f) deployment to 3D. The figure explains the pipeline without requiring the caption. The previous Fig. 1 showing physical photographs has been moved to the Supplementary Information.

Figures 2 to 5 have also been revised. Panels have been expanded, annotations added, and visual clarity improved so that each figure communicates its content independently of the caption. The revised figures are included in the resubmitted manuscript. In addition, all figures have been reformatted to comply with Nature Communications figure guidelines regarding font size, panel dimensions, and labeling conventions.

The revised Fig. 1 is reproduced below,

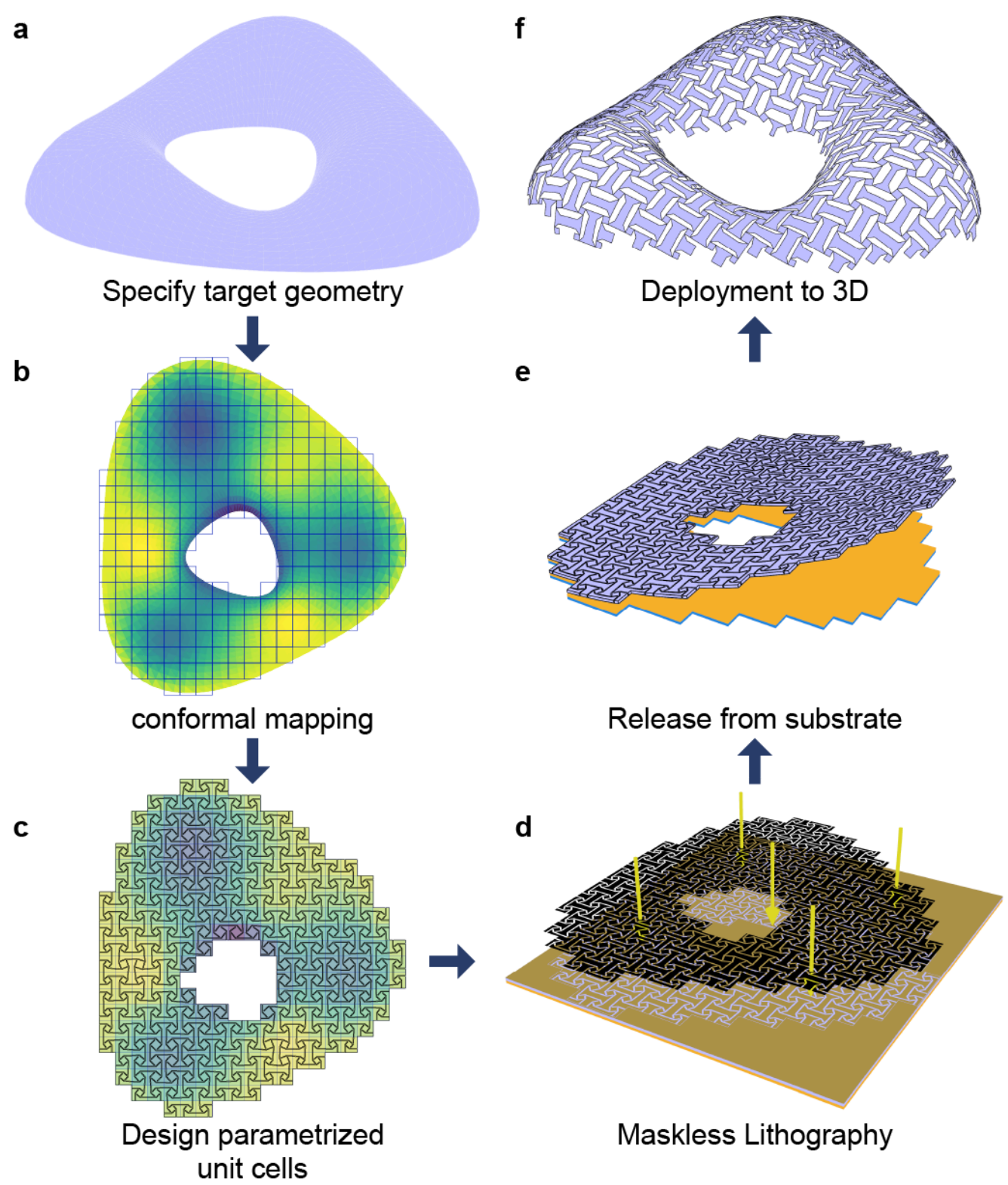

Figure R3. [Fig. 1 of the manuscript)] **Pipeline for the manufacturing of deployable 3D mesoscale structures.** a) A target 3D geometry is specified as a surface mesh. We use the example of an annulus with both positive and negative Gaussian curvature. b) The mesh is conformally flattened to the 2D plane; the color spectrum indicates the local isotropic expansion factor λ per mesh face, and a regular square grid is superimposed. c) The geometric parameters of each unit cell are tuned so that its second stable equilibrium occurs at the required local expansion λ. This yields a spatially heterogeneous 2D precursor pattern. d) The precursor pattern is transferred to a polyimide film via maskless lithography and reactive-ion etching. e) The patterned film is mechanically released from the wafer substrate. f) The free-standing precursor is deployed through mechanical indentation into the prescribed 3D geometry, where it is stabilized by the bistable nature of its constituent unit cells.

Reviewer #2 (Remarks to the Author):

This work proposes a wafer-fabricated deployable 3D architectures. A 2D precursor composed of bistable auxetic units is designed from a target conformal stretch field, doubly-curved shapes. The manuscript presents a deployed geometry and an optical demonstration based on a paraboloidal reflector. The concept is interesting, but this manuscript does not meet the journal's standards the manuscript.

Overall, the figures lack polish; their quality is below the standard expected for this journal.

We thank the reviewer for their assessment and for the detailed comments that follow. These comments have helped us strengthen the presentation and clarify the scope and contributions of the work.

The central result of this manuscript is that free-standing, mechanically stable, doubly-curved 3D structures with prescribed Gaussian curvature can be produced from standard wafer fabrication — a capability not previously demonstrated. We believe this result is of broad interest to the readership of Nature Communications, as also recognized by Reviewer 1. In the revised manuscript, we have made this contribution explicit from the outset, clarified the framing of the work as a manufacturing paper in which deployment is an integral fabrication step, substantially expanded the literature review, added quantitative fabrication and deployment characterization, and thoroughly redesigned all figures to meet the journal's presentation standards.

We address the reviewer's comments below.

R2C1: The abstract is too long. It should be revised to concisely state what the paper discusses. In particular, the first 2–3 sentences appear unnecessary.

We thank the reviewer for this suggestion. We agree that the original abstract was too long and contained introductory material that is better suited to the main text. Following the reviewer's recommendation, we have revised the abstract to concisely state the key contributions of the work. Specifically, we have removed the opening sentences motivating the importance of 3D architectures for human-interacting electronics, as well as the discussion of limitations of current shape morphing strategies, and replaced them with a direct statement of the proposed approach. The revised abstract reads as follows:

We demonstrate that standard wafer fabrication can produce free-standing, mechanically stable, doubly-curved 3D structures with prescribed Gaussian curvature --- a capability not previously achieved through semiconductor manufacturing. The stability of the deployed structures is realized through the bistable nature of their constituent auxetic unit cells. To impose prescribed Gaussian curvatures through deployment, we conformally flatten the target 3D mesh onto the 2D plane and locally tune the microstructure of each unit cell such that its second stable equilibrium occurs at the required isotropic expansion. The resulting precursor features a spatially heterogeneous tessellation. This generative method is validated on a spherical cap deployed from a flat disk through indentation, with deployment accuracy and structural stability confirmed numerically and experimentally. The method is further applied to a range of complex 3D shapes with both positive and negative curvatures. As a functional demonstration, paraboloidal reflectors with tunable focal lengths are fabricated and deployed, with reflected patterns

agreeing with geometric optics predictions. This work paves the way from flexible electronics towards *deployable electronics*, broadening the spectrum of realizable 3D semiconductor devices.

R2C2: The Introduction does not clearly state the scope or questions the manuscript intends to address.

We thank the reviewer for this observation. We agree that the original introduction did not clearly state the scope and central questions of the manuscript upfront. We have revised the introduction to address this. Specifically, we have added a clear scope statement early in the introduction, immediately after the discussion of the two fabrication paradigms, that explicitly poses the central question the manuscript addresses and summarizes the approach taken to answer it:

Here we ask: can standard planar wafer fabrication be used to manufacture precursors that deploy uniquely, stably, and accurately to prescribed doubly-curved 3D geometries without a supporting substrate? We address this question by combining conformal mapping, bistable auxetic microstructure design, and multi-step lithography into a unified fabrication and deployment pipeline. This approach leverages existing planar wafer fabrication, where parallel processing, yield, and heterogeneous material integration are already mature, and combines it with mechanically programmed, bistable deployment to achieve prescribed three-dimensional architectures. We treat the 2D-to-3D deployment as an integral step of the manufacturing process: the planar precursor fabricated on the wafer is an intermediate product, and the deployed 3D structure is the final manufactured device [14-16].

R2C3: Is the paper focused on a manufacturing (wafer-fabricated process) or on a structural design/properties enabled by the kirigami architecture?

We thank the reviewer for this important question. We would like to clarify that this manuscript is primarily a manufacturing paper. The central result is that free-standing, mechanically stable, doubly-curved 3D structures with prescribed Gaussian curvature can be produced from standard wafer fabrication. This is a capability that has not been previously demonstrated.

Crucially, we argue that the 2D-to-3D deployment step is an integral part of the manufacturing process rather than a separate structural demonstration. Since the end goal is a 3D device, the deployment is the final manufacturing step that yields the target geometry. The planar precursor fabricated on the wafer is an intermediate product; the deployed 3D structure is the final manufactured device. The conformal mapping algorithm, bistable unit-cell design, and release protocol are presented as enabling components of this unified fabrication pipeline.

We have revised the introduction to make this framing explicit from the outset, emphasizing that the 2D-to-3D transformation is part of the manufacturing process and that the paper's contribution is the demonstration of this complete pipeline.

The central result of this work is that free-standing, mechanically stable, doubly-curved 3D structures with prescribed Gaussian curvature can be produced from standard wafer fabrication. We treat the 2D-to-3D deployment as an integral step of the manufacturing process: the planar precursor fabricated on the wafer is an intermediate product, and the deployed 3D structure is the final manufactured device. We show 1) that masked lithography can fabricate bistable auxetic precursor structures with spatially

heterogeneous aperiodic patterns algorithmically designed to target specific 3D shapes, 2) that these wafer-fabricated precursors can be released from the substrate and mechanically deployed uniquely and stably to the target 3D geometry without a supporting substrate, and 3) that the resulting free-standing 3D structures are mechanically stable and geometrically accurate, as confirmed through numerical simulation and experiment.

R2C4: The literature review is insufficient for the topics in this manuscript.
Two-photon polymerization (TPP) is mentioned early, but I think that there is no relationship between "wafer-fabrication" in this manuscript and the TPP.

We thank the reviewer for this comment. TPP and wafer-scale fabrication are fundamentally different processes and their relationship requires clear framing. TPP is included in the introduction solely to provide context for the broader landscape of existing approaches to direct 3D microfabrication, as the most prominent alternative route to achieving 3D microstructures. The comparison is intentional: by positioning the two paradigms side by side, we highlight that our approach offers a complementary and distinct pathway to free-standing 3D architectures that is fully compatible with existing semiconductor manufacturing workflows.

We have substantially expanded the literature review in the revised manuscript. In response to **R1C1**, we have added new references covering recent advances in MPL and two-photon polymerization [7-13], recent demonstrations of planar-to-3D fabrication paradigms [4-6], and the fundamental distinctions between serial resist-based writing and parallel wafer-scale processing. We have additionally expanded the discussion of bistable auxetic kirigami patterns in the literature, adding references to recent work on multistable kirigami metamaterials and shape morphing. Together these additions provide a more comprehensive review of the relevant literature across all three topics central to this manuscript: direct 3D microfabrication, deployable bistable structures, and wafer-scale fabrication of flexible architectures.

The revised introduction is as follows,

Current efforts to realize three-dimensional electronic and photonic systems follow two distinct fabrication paradigms: direct three-dimensional microfabrication, and planar wafer fabrication followed by transformation or assembly into three-dimensional forms [4-6]. Recent advances in multiphoton lithography (MPL), including two-photon polymerization, have significantly expanded the capabilities of the former, enabling freeform 3D architectures, writing on non-planar substrates, and a range of hybrid and transfer-based integration strategies [7-10].

With the shared long-term goal of digital electronics in 3D, MPL advances this objective by offering geometric freedom and localized material patterning through serial, voxel-by-voxel writing [11]. This serial nature, together with the reliance on material-specific photoresists and sequential processing steps, represents a fundamental distinction from wafer-scale fabrication and can pose challenges for large-area scalability, throughput, and heterogeneous integration within established semiconductor workflows [12,13].

References:

[4] Song, Z. et al. Kirigami-based stretchable lithium-ion batteries. Scientific reports 5, 10988 (2015).
[5] Guo, X. et al. Controlled mechanical assembly of complex 3D mesostruc- tures and strain sensors by tensile buckling. npj Flexible Electronics 2, 14 (2018).
[6] Zhang, X., Zhang, X., Duan, Y., Zhang, L. & Ni, X. All-optical geometric image transformations enabled by ultrathin metasurfaces. Nature communications 14, 8374 (2023).
[7] den Hoed, F. M. et al. Facile handling of 3d two-photon polymerized microstructures by ultra-conformable freestanding polymeric membranes. Advanced Functional Materials 33, 2214409 (2023).
[8] Zyla, G. & Farsari, M. Frontiers of laser-based 3d printing: a perspective on multi-photon lithography. Laser & Photonics Reviews 18, 2301312 (2024).
[9] Zyla, G. et al. Film-based multi- photon lithography for efficient printing of electromagnetic surface structures. Advanced Materials Technologies 10, 2402137 (2025).
[10] Arriaga-D´avila, J. et al. From sin- gle to multi-material 3d printing of glass-ceramics for micro-optics. Small Methods 2401809 (2025).
[11] Gu, S. et al. 3d nanolithography with metalens arrays and spatially adaptive illumination. Nature 648, 591–599 (2025).
[12] Kawata, S., Sun, H.-B., Tanaka, T. & Takada, K. Finer features for functional microdevices. Nature 412, 697–698 (2001).
[13] Gansel, J. K. et al. Gold helix photonic metamaterial as broadband circular polarizer. Science 325, 1513–1515 (2009).

R2C5: The information in Fig. S3 is very unclear. While the Jacobian matrix and the formulas seem to follow a parabolic-equation form, Equations (5), (6), (7), and (8) are not presented in a way that allows easy verification.

We thank the reviewer for pointing out the lack of clarity in SI Fig. S3 and the associated equations. We agree that the presentation of the stereographic projection and the Jacobian calculation needed improvement. In the revised SI, we have clarified the definition and direction of the mapping, explicitly distinguished the forward projection (SI Eq. 6) and inverse projection (SI Eq. 7), and specified that the Jacobian matrix (SI Eq. 8) is evaluated with respect to (u, v) at a given point Q. Additional explanatory text has been added to guide the reader through each step of the derivation leading to the scale factor $\lambda(Q)$ in SI Eq. 9. SI Fig. S3 has been updated to more clearly label the relevant points, coordinates, and tangent vectors J**X** and J**Y**.

We have replicated our revised SI Sec. 2.2 Conformal map and stereographic projection for the updated analysis. This is replicated below where the new text is displayed in red,

2.2 Conformal map and stereographic projection

A conformal map preserves local angles, meaning the neighborhood of each point is scaled isotropically; it stretches or shrinks evenly in all directions without shear distortion. This allows us to flatten a 3D surface onto a plane while preserving local angles at every material point, at the cost of changing areas.

Consider a map $f: \Omega \rightarrow \mathbb{R}^3$, where $\Omega \subset \mathbb{R}^2$ and f maps this domain to a surface in $\mathbb{R}^3$. For a point $\mathbf{Q} \in \Omega$ and a vector $\mathbf{X}$ at $\mathbf{Q}$, the pushforward of $\mathbf{X}$ under f is given by $J\mathbf{X}$, where J is the Jacobian

matrix of f at $\mathbf{Q}$. Intuitively, $J\mathbf{X}$ describes how the map f transforms the 2D vector $\mathbf{X}$ into a tangent vector on the surface in $\mathbb{R}^3$. We say that f is conformal if, for any point $\mathbf{Q} \in \Omega$,

$$J\mathbf{X} \cdot J\mathbf{Y} = \lambda(\mathbf{Q})^2 \mathbf{X} \cdot \mathbf{Y}$$

where $\lambda(\mathbf{Q}) > 0$ is the local scale factor of the mapping. Geometrically, this means that the map scales all vectors at $\mathbf{Q}$ by the same factor $\lambda(\mathbf{Q})$ regardless of their direction. This is the defining property of isotropic scaling. This relation also indicates that $\lambda(\mathbf{Q})$ can be determined from the mapped vectors $J\mathbf{X}$ and $J\mathbf{Y}$ in $\mathbb{R}^3$ and their pre-images $\mathbf{X}$ and $\mathbf{Y}$ in $\mathbb{R}^2$ [3].

Stereographic projection provides a conformal map from the sphere to the plane, preserving local angles and mapping circles on the sphere to circles (or straight lines) in the planar domain [4]. Here, we use this mapping to explicitly derive the local scale factor λ for transforming a quarter sphere (*i.e.*, a spherical cap with height equal to one quarter of the sphere diameter) into a planar precursor. As shown in Fig. S3, we consider the unit sphere S^2 in $\mathbb{R}^3$, as defined by the set of all points (x, y, z) satisfying $x^2 + y^2 + z^2 = 1$. The south pole is the point $\mathbf{S} = (0, 0, -1)$, and we let $S^2\backslash\{\mathbf{S}\}$ represent the sphere excluding the south pole. In stereographic projection, each point $\mathbf{P} \in S^2\backslash\{\mathbf{S}\}$ is mapped onto the uv-plane (i.e., the plane $z = 0$) by drawing a line from the south pole $\mathbf{S}$ through $\mathbf{P}$ and identifying its intersection with the plane at $\mathbf{Q}$. For the quarter sphere (colored in red in Fig. S3), points lying on the same circle of latitude (e.g., the rim of the dome) are projected onto concentric circles centered at the origin in the uv-plane, thereby defining the boundary of the planar domain corresponding to the quarter-sphere projection.

Using a simple Cartesian coordinate transformation, the projection between $\mathbf{Q}(u, v)$ and $\mathbf{P}(x, y, z)$ can be expressed as:

$$(u, v) = \left(\frac{x}{1-z}, \frac{y}{1-z}\right)$$

And

$$(x, y, z) = \left(\frac{2u}{\xi}, \frac{2v}{\xi}, \frac{\xi-2}{\xi}\right),$$

Where

$$\xi = 1 + u^2 + v^2.$$

The Jacobian matrix of the mapping is given by the partial derivatives of (x, y, z) with respect to (u, v), *i.e.*,

$$J = \begin{bmatrix} \frac{\partial x}{\partial u} & \frac{\partial x}{\partial v} \\ \frac{\partial y}{\partial u} & \frac{\partial y}{\partial v} \\ \frac{\partial z}{\partial u} & \frac{\partial z}{\partial v} \end{bmatrix} = \begin{bmatrix} \frac{2\xi - 4u^2}{\xi^2} & \frac{-4uv}{\xi^2} \\ \frac{-4uv}{\xi^2} & \frac{2\xi - 4v^2}{\xi^2} \\ \frac{4u}{\xi^2} & \frac{4v}{\xi^2} \end{bmatrix}$$

For a point $\mathbf{Q}(a, b)$ in the uv-plane, substituting $u = a$ and $v = b$ into Eq. (8) yields the Jacobian matrix at $\mathbf{Q}$:

$$J_Q = \begin{bmatrix} \frac{2\gamma - 4a^2}{\gamma^2} & \frac{-4ab}{\gamma^2} \\ \frac{-4ab}{\gamma^2} & \frac{2\gamma - 4b^2}{\gamma^2} \\ \frac{4a}{\gamma^2} & \frac{4b}{\gamma^2} \end{bmatrix}, \quad \gamma = 1 + a^2 + b^2$$

We consider any two in-plane vectors $\mathbf{X} = (u_1, v_1)$ and $\mathbf{Y} = (u_2, v_2)$ defined at $\mathbf{Q}$. The directional derivative of f at $\mathbf{Q} \in \Omega$ along the direction $\mathbf{X} = (u_1, v_1)$ is given by:

$$J\mathbf{X} = \left[\frac{2\gamma - 4a^2}{\gamma^2}u_1 - \frac{2ab}{\gamma^2}v_1, -\frac{4ab}{\gamma^2}u_1 + \frac{2\gamma - 4b^2}{\gamma^2}v_1, \frac{4a}{\gamma^2}u_1 + \frac{4b}{\gamma^2}v_1\right]$$

Similarly, the directional derivative of f at $\mathbf{Q}$ along the direction $\mathbf{Y}$ is given by:

$$J\mathbf{Y} = \left[\frac{2\gamma - 4a^2}{\gamma^2}u_2 - \frac{2ab}{\gamma^2}v_2, -\frac{4ab}{\gamma^2}u_2 + \frac{2\gamma - 4b^2}{\gamma^2}v_2, \frac{4a}{\gamma^2}u_2 + \frac{4b}{\gamma^2}v_2\right]$$

Once simplified, the inner product of the two three-dimensional vectors JX and JY, after being mapped, is as follows,

$$J\mathbf{X} \cdot J\mathbf{Y} = \frac{4(u_1u_2 + v_1v_2)}{\gamma^2}$$

From Equation (5),

$$\lambda(\mathbf{Q}) = \sqrt{\frac{J\mathbf{X} \cdot J\mathbf{Y}}{\mathbf{X} \cdot \mathbf{Y}}}$$

Since $\mathbf{X} \cdot \mathbf{Y} = u_1u_2 + v_1v_2$, and substitute Equation (12) into Equation (13), we get

$$\lambda(\mathbf{Q}) = \frac{2}{\gamma} = \frac{2}{1 + a^2 + b^2}$$

which shows that the scale factor $\lambda(\mathbf{Q})$ depends only on the coordinate of the point $\mathbf{Q}$ and is independent of the chosen in-plane directions $\mathbf{X}$ and $\mathbf{Y}$. This reflects the conformal property of the mapping, whereby the neighborhood of a point is scaled isotropically in all directions. Consequently, this relation quantifies how stereographic projection locally scales planar vectors when mapping the domain onto the three-dimensional surface.

R2C6: Overall, the figures are too small and have poor readability. In Fig. 1, the conceptual explanation is insufficient, making it very difficult to understand the idea from the figure alone; appropriate explanatory text should be added.

We thank the reviewer for this feedback. We have completely redesigned Fig. 1 to address these concerns. The new figure uses the annulus geometry as a representative example and presents the complete manufacturing pipeline as a fully conceptual schematic with six panels: a) target geometry, b) conformal mapping with λ colorfield and unit cell grid, c) parametrized unit cell design, d) maskless lithography schematic, e) release from substrate, and f) deployment to 3D. Each panel is accompanied by a descriptive label that makes the pipeline self-explanatory without requiring the caption. The previous Fig. 1 showing physical photographs has been moved to the Supplementary Information.

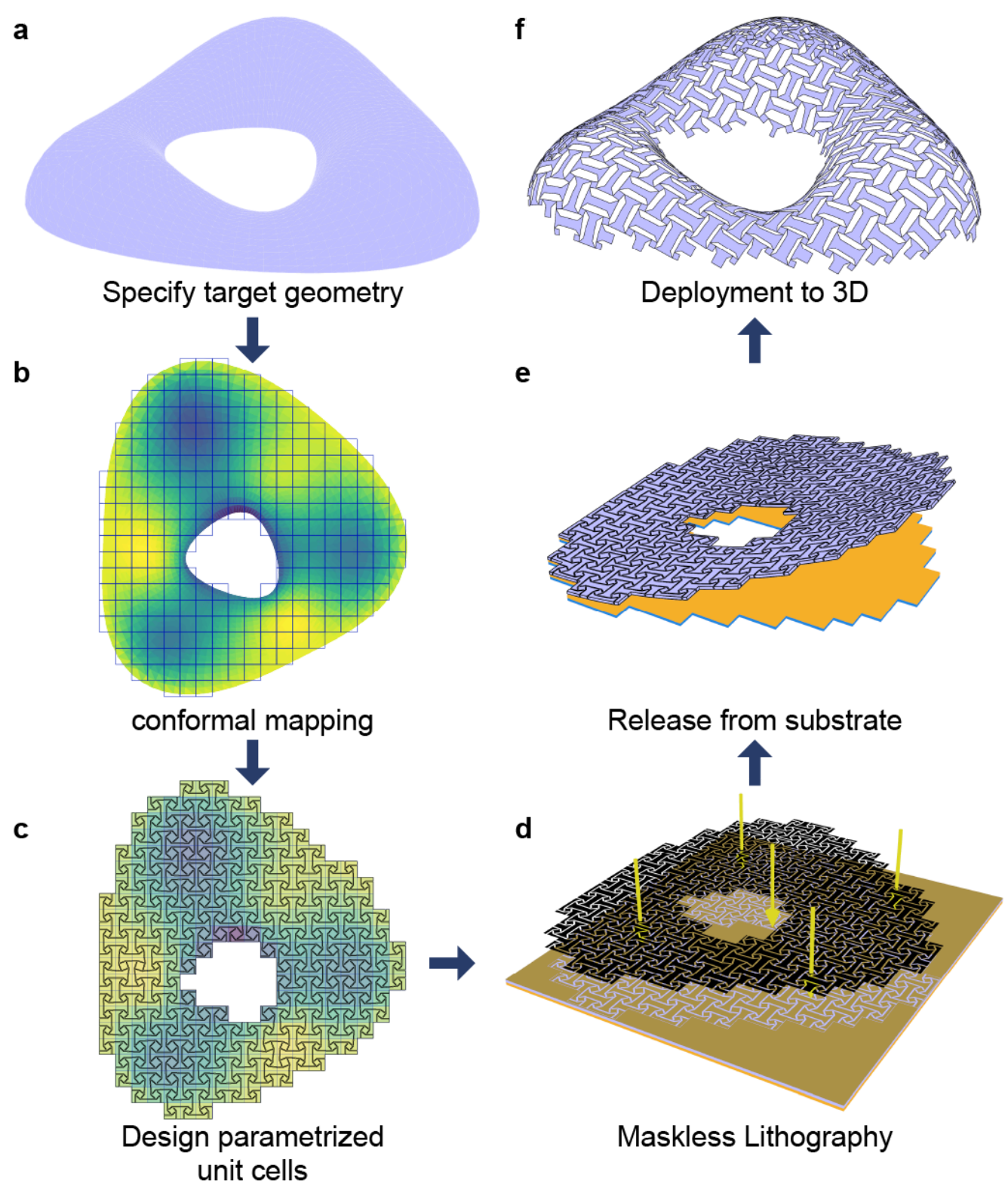

Figure R4. [Fig. 1 of the manuscript] **Pipeline for the manufacturing of deployable 3D mesoscale structures.** a) A target 3D geometry is specified as a surface mesh. We use the example of an annulus with both positive and negative Gaussian curvature. b) The mesh is conformally flattened to the 2D plane; the color spectrum indicates the local isotropic expansion factor λ per mesh face, and a regular square grid is superimposed. c) The geometric parameters of each unit cell per square are tuned such that its second stable equilibrium occurs at the required local expansion λ, yielding a spatially heterogeneous 2D precursor pattern. d) The precursor pattern is transferred to a polyimide film via maskless lithography and reactive-ion etching. e) The patterned film is mechanically released from the wafer substrate. f) The free-standing precursor is deployed through mechanical indentation into the prescribed 3D geometry, where it is stabilized by the bistable nature of its constituent unit cells.

R2C7: In Fig. 2(b), the conditions under which the reported color spectrum was obtained are not specified; likewise, the manuscript seems not to state under what conditions the discrete conformal map was computed.

We thank the reviewer for this observation. We agree that we need to state more explicitly the conditions under which the color spectrum and conformal map are computed. In Fig. 2, the stretch values at the second equilibrium for each of the two unit cells are directly computed from the finite element parametric sweep. No conformal mapping is involved. For Fig. 3a/b, the color spectrum represents the local scale factor λ computed from the discrete conformal map of the target 3D geometry. We have added the explicit formula to the main text:

Regarding the conditions under which the conformal map is computed: for the hemisphere specifically, stereographic projection provides an analytical conformal map, the derivation of which is given in SI Sec. 2.2. For general target geometries given as surface meshes, the Boundary First Flattening (BFF) algorithm is used, which minimizes angular distortion while flattening the 3D mesh onto the 2D plane. Once the mesh is flattened, λ is computed per mesh face using the formula above. We have clarified this in the revised main text and SI Sec. 2.3.

While stereographic projection may be used to calculate a continuous λ field for such a canonical geometry [48] (see SI Sec. 2.2), we use Boundary First Flattening (BFF) [41] for generality in processing arbitrary discrete geometries (see SI Sec. 2.3).

BFF computes conformal maps of input meshes by minimizing angular distortion while flattening from 3D to 2D. The local scale factor λ at each mesh face is computed as the areal change of each pair of corresponding mesh faces, *i.e.*, $\lambda = \sqrt{A_{3D}/A_{2D}}$, where A_{3D} and A_{2D} are the areas of the corresponding mesh faces in 3D and 2D respectively (Fig.3a,ii).

R2C8: In Fig. 1(c), the concept of multi-step lithography is not conveyed at all. In Fig. 1(e), it is unclear why two hemispherical images are shown; these appear to be deployed patterns, but if so, the definition of the unit design is not evident.

We thank the reviewer for these observations. We have completely redesigned Fig 1 in the revised manuscript. The previous panel (c) showed a microscopy photograph of the fabricated 2D precursor, which did not convey the multi-step lithography process. In the new Fig. 1, panel (d) shows a clear schematic of the maskless lithography step, depicting the UV exposure of the patterned polyimide film on the wafer substrate.
The previous panel (e) showed the deployed cornea structure placed on a 3D printed eye model, with a small inset of the eye model without the cornea. This caused confusion as the reviewer notes. In the new Fig. 1, panel (f) shows a render of the deployed annulus structure in 3D. The deployed structure is labeled and its relationship to the 2D precursor in panel (e) is made explicit. Please see R2C7 for the revised figure.

R2C9: Additionally, in Fig. 1, Fig. 2, and Fig. 4s, panels (a–d) use black backgrounds, whereas the remaining figures use white backgrounds.

We have thoroughly revised all figures in the manuscript. We have ensured adherence to Nature Communications figure guidelines: all font sizes are 2.5mm, all figure widths are set to 88mm or 180mm, all fonts are sans-serif, and all panel labels are bold. Figure styles are now unified across all panels. All panels now use consistent white backgrounds.

R2C10: In Fig. 2(d), the units for stretch appear to be missing.
In short, the figure styles are not unified, and adherence to the journal's presentation standards is questionable.

We thank the reviewer for this observation. The stretch λ plotted in Fig. 2d is dimensionless, as it represents the ratio of deformed to undeformed length. We have added an explicit axis label noting that stretch is dimensionless.

We agree that the figure styles were not unified across the manuscript. As noted in our responses to R1C6 and R2C9, we have revised all manuscript and SI figures to adhere to Nature Communications figure guidelines. Specifically, we have: (1) set all figure widths to either 88mm or 180mm as required; (2) changed all fonts to sans-serif with a height of 2.5mm; and (3) made all panel labels bold. These changes have been applied consistently across all figures.

R2C11: Fig. 2(a) shows the auxetic pattern studied in this work. However, many auxetic patterns are possible, and the manuscript does not adequately justify why this particular pattern was chosen.

We thank the reviewer for this question. The rotating square bistable auxetic pattern used in this work is chosen because it simultaneously satisfies three properties that are necessary for the conformal deployment approach: (1) auxeticity with a constant Poisson's ratio of $\nu = -1$ between the rest configuration and the second equilibrium configuration; (2) mechanical bistability with a tunable second equilibrium strain; and (3) isotropic expansion over the entire strain range. These three properties are jointly required by the conformal deployment framework, and the rotating square pattern satisfies all of them by construction.

We note that the choice of this specific pattern is not fundamental to the method, any unit cell satisfying these three properties could in principle be substituted. Indeed, recent work has explored alternative bistable auxetic geometries including triangular rotating unit cells and hyperbolic tessellations. However, isotropic expansion is a critical requirement for the conformal mapping approach: conformal maps preserve angles but not areas, and the scale factor λ at each point represents a purely isotropic areal scaling. A unit cell that expands anisotropically would introduce shear distortion incompatible with the conformal map framework. The rotating square pattern satisfies this requirement by design, and its geometric parameters θ and l provide a continuous and monotonic tuning of λ needed to accommodate the curvature variation of the target surfaces. We have added a brief discussion of this point to the manuscript.

This design is chosen because it simultaneously satisfies three properties required by the conformal deployment framework: (1) a Poisson's ratio of $\nu = -1$ between the rest and second equilibrium configurations, (2) mechanical bistability with a tunable second equilibrium strain through the rotating square inclination θ and edge length l, and (3) isotropic expansion over the entire strain range [26, 35, 42,

43]. Isotropic expansion is critical because conformal maps prescribe a purely isotropic areal scaling λ at each point; a unit cell that expands anisotropically would introduce shear distortion incompatible with this framework.

R2C12: for Figures S1 and S8, figures contain two sub-figures (a) and (b), but the captions does not any descriptions of these subfigures.

We thank the reviewer for pointing this out and apologize for the oversight. We have revised the captions to clearly describe panels (a) and (b) for each figure. We have also reviewed the captions throughout the manuscript to ensure a consistent format. The following are the revised captions for the two figures respectively:

Figure S1: **Material characterization of polyimide under uniaxial tension.** a) Photograph of the tensile testing setup with a polyimide strip specimen clamped between the grips. b) Stress- strain response of polyimide, where the solid line denotes the mean value and the shaded region indicates the variation across three specimens.

Figure S8: **Finite element prediction of the indentation of the deployed dome.** a) Simulated deformation sequence of the deployed dome during indentation loading, with the color map indicating the von Mises stress distribution. b) Simulated force–displacement response obtained from the indentation simulation, where the displacement is normalized by the dome height.

R2C13: For Section S2.4, the manuscript states that the generative algorithm was implemented in Python and visualized using Rhino with the Grasshopper plugin.
Please specify the software versions and, for Rhino in particular, the relevant conditions used for visualization.
For the finite-element simulations, which version of Abaqus was used?

We thank the reviewer for this suggestion. We have added the software versions and a code availability statement to the revised SI. All lithography pattern generation is implemented in Python (CPython 3.9.10) and visualized using Rhinoceros 3D (Version 8, 8.27.26019) with Grasshopper (Build 1.0.0008). Rhino and Grasshopper are used for geometry visualization and DXF export to the maskless lithography system; no geometric modification or numerical computation is performed within the CAD environment. Finite element simulations are carried out using Abaqus/Standard (Dassault Systemes, version 2022). The Grasshopper definition files and Python scripts used for lithography pattern generation are available at github.com/UH-AIM.

R2C14: Fig. 4 demonstrates an impressive variety of deployed 3D structures. Nevertheless, the paper ultimately appears to target spherical geometries for application to the cornea. In that light, Fig. 4 does not seem to support the core application. It would be more appropriate to present structures that faithfully represent the spherical eye shape.
Also, “Target 3D shape” would be better phrased as “Target 3D design.”

We thank the reviewer for this comment and the suggested terminology change; we have replaced "target 3D shape" with "target 3D design" throughout the manuscript.

We would like to clarify that the goal of this paper is not to fabricate a cornea specifically, but rather to demonstrate that standard wafer fabrication can produce free-standing 3D structures with arbitrary prescribed Gaussian curvature. We have since replaced Fig. 1 of the main manuscript entirely to ensure this point is clear.

The cornea is one representative application among many. Fig. 4 is intended to demonstrate exactly this generality: the five structures span a range of topologies (Euler characteristic 0 and 1), curvature distributions (positive, negative, and mixed), and boundary conditions (closed and open). This breadth of demonstrated geometries is central to the paper's contribution.

Regarding geometric fidelity, we note that the cornea target geometry is not a simple sphere. It is defined directly from the anatomical cornea surface profile, and the conformal map and unit cell parameters are computed for this specific geometry. The deployed structure is superimposed onto a 3D-printed eye model from which the cornea has been removed, and the deployed shape conforms to the target surface. This demonstration, along with the quantitative geometric accuracy assessment of the dome, confirms that the method faithfully reproduces the prescribed geometry. This discussion has been moved to the SI Sec. 7 and reproduced below,

As a representative application, we demonstrate the deployment of a cornea-shaped geometry that is shown in Fig. 4a of the manuscript. The target geometry approximates the anterior surface of a human cornea, modeled as a doubly curved dome sitting atop a simplified eye geometry (Fig. S14a). The conformal map of this target surface yields a spatially heterogeneous λ field ranging from 1.2 at the periphery to 1.7 at the center (Fig. S14b), reflecting the higher Gaussian curvature near the apex. The corresponding 2D precursor is fabricated on-wafer using the lithography protocol described in SI Sec. 4 (Fig. S14c). After release from the substrate (Fig. S14d), the precursor is mechanically deployed onto a 3D-printed eye model from which the cornea has been removed, exposing the flat iris (Fig. S14e). The deployed structure conforms to the target cornea geometry and is mechanically stable without a supporting substrate. An inset reveals the spatially varying unit cell microstructure in the deployed state.

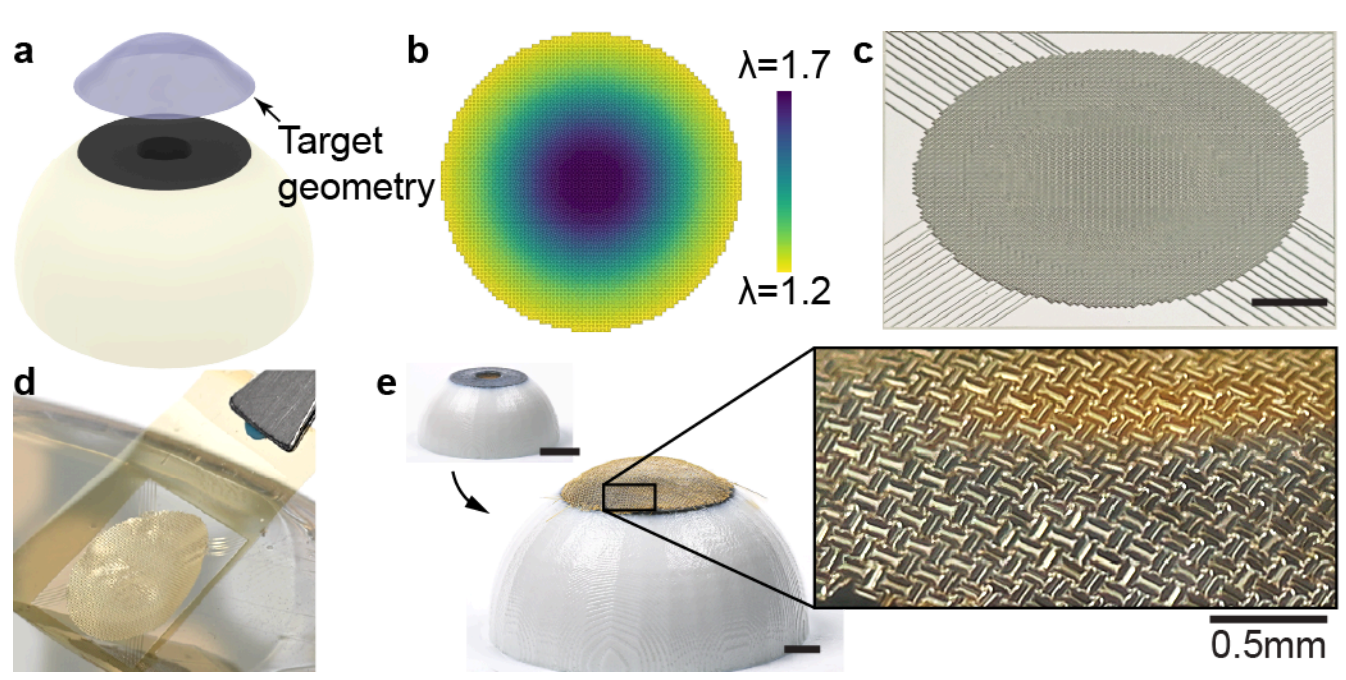

Figure R5. [Fig. S14] **Cornea device design and fabrication.** a) Target cornea geometry shown atop a simplified eye model. b) Conformal map of the target surface onto the 2D plane, with the color spectrum indicating the local isotropic expansion factor λ per mesh face. c) Microscopy image of the fabricated 2D

precursor on the wafer. d) Release of the patterned polyimide film from the substrate. e) Deployed cornea structure placed on a 3D-printed eye model with the cornea removed. Inset: magnified view of the deployed microstructure showing spatially varying unit cells. Scale bars, 5 mm.

R2C15: The manuscript presents a "paraboloidal reflector" as an application of the fabricated structures. The necessity of using the proposed structure specifically for a paraboloidal reflector is not established. The experiments provided are insufficient to substantiate the unique advantages of the proposed design.

We thank the reviewer for this comment. We agree that the paraboloidal reflector should not be interpreted as the primary application, nor as an attempt to develop an optimized optical device. Rather, it serves as a proof-of-concept to validate the geometric accuracy and curvature control of the deployed structures. Paraboloidal geometries provide a well-defined benchmark for this purpose: their optical response is highly sensitive to surface curvature deviations, and the reflection pattern under collimated illumination can be analytically predicted, allowing direct comparison with experiment. The agreement between predicted and observed patterns confirms that the deployed surfaces reproduce the designed curvature with reasonable fidelity.

At the same time, paraboloidal reflectors highlight a potential advantage of the proposed approach. Curved reflective surfaces are typically fabricated by direct 3D manufacturing. In contrast, our method begins with planar wafer fabrication, which readily supports multilayer processing and integration of additional functional materials such as sensing or electronic components.

We have revised the manuscript to clarify these points. The revised text is reproduced below.

We use parabolic reflectors as a representative geometry to demonstrate precise control over the curvature of the deployed 3D structures. Parabolic geometries provide a well-defined benchmark: their optical response is highly sensitive to surface curvature deviations, and the reflection pattern under collimated illumination can be analytically predicted. Furthermore, conventional fabrication of three-dimensional reflective surfaces often relies on high-precision machining, molding, or micro-scale 3D printing [56]. In contrast, our planar wafer-based fabrication strategy offers practical advantages for device integration, since wafer fabrication readily supports multilayer processing and the incorporation of additional functional materials such as sensing or electronic components.

R2C16: The title of this manuscript is included "wafer-fabricated," the work reads as manufacturing-centric. If the primary claim lies in wafer-scale fabrication, the manuscript should include the various evaluation about the quality of fabrication (accuracy, fabrication yield, and durability).

We thank the reviewer for this comment. We would like to clarify that the term "wafer-fabricated" in the title is intended to highlight that the free-standing, substrate-free 3D structures originate from a planar 2D precursor fabricated using standard wafer processes, rather than to position the work as a study of wafer fabrication itself. Nevertheless, we agree that the manuscript benefits from including evaluation of fabrication quality and process viability.

In response, we have added the following characterization data to the revised manuscript and SI:

Fabrication accuracy: We have added a dedicated subsection (SI Sec. 4.1) reporting measured film thicknesses, designed versus fabricated feature sizes of the critical slit width s and hinge width c, and the inverse pattern correction applied to compensate for systematic deviations (see R1C2).

Deployment repeatability: Three nominally identical dome structures are fabricated and deployed, yielding dome heights of 0.671 ± 0.018 mm and lateral spans of 2.29 ± 0.13 mm (mean ± s.d.), confirming consistent deployment behavior across samples (see R1C3).

Geometric accuracy: A 3D surface reconstruction of a representative deployed dome is compared pointwise to the target geometry, with deviations concentrated at the rim ($\delta \leq 0.14R$) and the central region closely matching the target (see R1C3).

Structural durability: The indentation experiment demonstrates the mechanical stability of the deployed structure under loading. We have discussed the damage mechanisms observed under off-path loading, specifically localized hinge plasticity and buckling, and clarified that these do not originate from the deployment process (see R1C4).

We would like to thank both reviewers for supporting the publication of our manuscript.
