# Supplementary material for: Deployable 3D architectures from wafer-fabricated precursors
Source: Nat Commun. 2026 Jul 25;17:9047. doi: 10.1038/s41467-026-76052-y (PMC13507144; doi:10.1038/s41467-026-76052-y)
Supplement: Supplementary file 3 [file 41467_2026_76052_MOESM3_ESM.pdf]

Description of Additional Supplementary Files

File Name: Supplementary Movie 1
Description: In-situ optical microscopy of an 8x8 periodic unit-cell tessellation under uniaxial tension.

File Name: Supplementary Movie 2
Description: Deployment and indentation of a hemispherical dome.

File Name: Supplementary Movie 3
Description: 360-degree rotation of the deployed 3D structures shown in Fig. 4.
